# The effects of exercise training and nutritional supplementation on taekwondo performance: a systematic review and meta-analysis

**DOI:** 10.3389/fnut.2025.1618612

**Published:** 2025-12-11

**Authors:** Chen Xu, Wenxin Zhang, Lin Luo

**Affiliations:** 1 School of Physical Education, Guizhou Normal University, Guiyang, China; 2 Key Laboratory of Brain Function and Brain Disease Prevention and Treatment of Guizhou Province, Guiyang, China

**Keywords:** taekwondo, training, supplementation, agility, repeated-kick, VO_2_max, meta-analysis

## Abstract

**Background:**

Taekwondo involves dynamic kicking and intermittent high-intensity efforts; the quantitative effects of training and supplementation on sport-specific outcomes remain unclear.

**Objective:**

To systematically quantify the effects of exercise training and nutritional supplementation on taekwondo-specific performance indicators—TSAT, FSKT (10-s and multiple-bout), CMJ, VO_2_max, and heart-rate indices (HRmean, HRmax, HRpeak)—and to explore potential moderators.

**Methods:**

A PRISMA-guided systematic review and random-effects meta-analysis (SMD, 95% CI) were conducted on randomized or quasi-experimental studies involving taekwondo athletes. Risk of bias was assessed using RoB 2.0. Primary outcomes included TSAT and FSKT performance; secondary outcomes included CMJ, VO_2_max, and HR indices.

**Results:**

Exercise training significantly improved TSAT (SMD = −0.82; 95% CI: −1.43 to −0.21), FSKT-10s (SMD = 0.82; 95% CI: 0.15–1.49), FSKT-mult (SMD = 0.95; 95% CI: 0.55–1.35), and VO_2_max (SMD = 1.54; 95% CI: 0.58–2.49); CMJ (SMD = 0.21; 95% CI: −0.02–0.45) and HRmax (SMD = −0.02; 95% CI: −0.48–0.44) showed no significant changes. Nutritional supplementation—especially caffeine—improved TSAT (SMD = −1.41; 95% CI: −2.24 to −0.57), FSKT-10s (SMD = 1.82; 95% CI: 1.08–2.57), FSKT-mult (SMD = 1.67; 95% CI: 0.72–2.62), and VO_2_max (SMD = 0.95; 95% CI: 0.60–1.31), with no effect on HR_mean (SMD = 0.10; 95% CI: −0.28–0.47) or HRpeak (SMD = 0.28; 95% CI: −0.46–1.02).

**Conclusion:**

Both exercise training and nutritional supplementation significantly improve agility, repeated-kick performance, and aerobic capacity in taekwondo athletes. Nevertheless, the findings should be generalized cautiously due to the observed heterogeneity. Future well-designed, adequately powered randomized controlled trials with standardized protocols are warranted.

**Systematic review registration:**

Identifier: CRD420251007058.

## 1 Introduction

Taekwondo is a dynamic Olympic combat sport characterized by rapid, high-precision kicking techniques and complex tactical execution (1–3). In contrast to boxing, judo, or wrestling, taekwondo performance primarily depends on high-speed, high-accuracy kicks aimed at specific scoring zones to gain tactical superiority or achieve technical knockdowns (4, 5). Since its inclusion in the Olympic Games in 2000, taekwondo has expanded globally, with increasingly demanding competition formats that require athletes to sustain repeated explosive actions interspersed with brief recovery periods—an effort pattern that mirrors a high-intensity interval training (HIIT) profile (6).

These physiological demands necessitate superior aerobic and anaerobic capacities, neuromuscular power, agility, and flexibility to efficiently utilize the glycolytic energy system (2, 3). The sport’s kinematic profile—comprising rapid kicking sequences, intricate footwork, ballistic jumping attacks, and dynamic defensive maneuvers—requires specialized physiological adaptations (4, 5). To evaluate these multidimensional demands, several validated sport-specific and general performance tests are commonly used. The Taekwondo-Specific Agility Test (TSAT) and the Frequency-Speed Kick Test (FSKT) assess agility, technical accuracy, and fatigue resistance (6, 7), while the countermovement jump (CMJ) quantifies lower-limb explosive power, which is crucial for ballistic kicking performance (8, 9). Aerobic capacity indicators, including maximal oxygen uptake (VO₂max) and heart-rate indices (HR_mean, HR_max, HR_peak), further reflect cardiovascular efficiency and recovery ability (10–12).

This integrative assessment framework has been extensively validated and widely applied in taekwondo sport-science research to capture the foundational determinants of performance (13, 14). Nonetheless, taekwondo performance remains multifactorial, encompassing physiological, technical, tactical, and psychological dimensions (15). Over the past decade, research has increasingly investigated exercise-based and nutritional interventions targeting these performance components. Exercise training—including plyometric, resistance, and HIIT protocols—has been shown to enhance neuromuscular strength, agility, and endurance (16–20). Meanwhile, nutritional supplementation—such as caffeine, creatine, β-alanine, and vitamin D—has demonstrated potential to optimize energy metabolism, delay fatigue, and improve recovery (21–23). However, considerable interindividual variability in response to these interventions persists, influenced by sex, dosage, genetic factors, and training experience (24–28). Approximately 20–30% of athletes are reported to be “non-responders,” underscoring the complexity of tailoring interventions to specific physiological profiles (26, 29, 30).

Previous reviews have often aggregated findings across heterogeneous combat sports or focused on single interventions, limiting sport-specific interpretability for taekwondo. Methodological inconsistencies—including small sample sizes, short intervention durations, and heterogeneous assessment protocols—further constrain the generalization of findings (28, 31–33). Consequently, there remains a lack of comprehensive synthesis evaluating both exercise and nutritional strategies within a unified analytical framework.

To address this gap, the present systematic review and meta-analysis aimed to synthesize evidence on the effects of exercise training and nutritional supplementation on taekwondo-specific and general performance outcomes. The primary outcomes were TSAT, FSKT-10s, and FSKT-mult, which directly reflect sport-specific agility and repeated-kick performance. The secondary outcomes included CMJ, VO_2_max, and heart-rate indices (HR_mean, HR_max, HR_peak), representing broader physiological capacities. Furthermore, methodological and participant-related moderators were explored to identify potential sources of heterogeneity and to inform evidence-based performance enhancement in taekwondo athletes.

## 2 Methods

### 2.1 Protocol and registration

This systematic review and meta-analysis was conducted in accordance with the Preferred Reporting Items for Systematic Reviews and Meta-Analyses (PRISMA) 2020 guidelines. The study protocol was prospectively registered with the International Prospective Register of Systematic Reviews (PROSPERO) prior to data collection and literature retrieval (registration number: CRD420251007058).

### 2.2 Study design

This study adopted a systematic review and meta-analysis design aimed at comprehensively evaluating the effects of exercise and nutritional supplementation on physical performance in taekwondo athletes.

### 2.3 Data sources and search strategy

The literature search covered studies published from the inception of each database up to February 27, 2025. A comprehensive search was conducted in PubMed, Scopus, and Web of Science (WoS). Keywords related to the study objectives were applied to titles, abstracts, and full texts to maximize the retrieval of potentially eligible studies (detailed search strategies are provided in Appendix 1).

To enhance both accuracy and efficiency, the search syntax was customized for each database: [Title/Abstract] in PubMed, TITLE-ABS-KEY in Scopus, and TS (Topic) in WoS. Boolean operators (AND, OR, NOT) were employed to refine keyword combinations. Studies involving patient or clinical populations were excluded to ensure that only those focusing on taekwondo athletes were retained.

The search was confined to peer-reviewed English-language journal articles. Grey literature—including theses, dissertations, conference proceedings, and non–peer-reviewed reports—was not considered in order to maintain methodological consistency and ensure data transparency across studies.

### 2.4 Inclusion and exclusion criteria

#### 2.4.1 Inclusion criteria

To ensure methodological rigor and practical relevance, studies were eligible for inclusion if they met the following criteria (see Table 1).

**Table 1:** Inclusion criteria based on the PICOS framework.

PICOS	Content
P (Patients/Problem)	Healthy taekwondo athletes of all age groups
I (Intervention)	Exercise training and/or nutritional supplements
C (Comparison)	Placebo group or non-intervention (control) group
O (Outcomes)	Taekwondo-Specific Agility Test (TSAT), Frequency Speed of Kick Tests (FSKT), countermovement jump (CMJ), maximal oxygen uptake (VO₂max), and heart rate (HR) parameters
S (Study design)	Randomized controlled trials (RCTs) or randomized crossover trials, published in English

To ensure clarity in the interpretation of study outcomes, operational definitions and their corresponding performance capacities are detailed in Table 2. Primary outcomes were prespecified as taekwondo-specific performance measures (TSAT, FSKT-10s, FSKT-mult) whereas secondary outcomes were defined as general physical fitness indicators (CMJ, VO₂max) and cardiovascular responses (HR_mean, HR_max, HR_peak). This hierarchy reflects sport-specific relevance and anticipated responsiveness to interventions.

**Table 2:** Definitions of outcome measures and corresponding physical abilities.

Index	Definition	Physical ability assessed
TSAT (Taekwondo-Specific Agility Test)	Time taken to complete a standardized taekwondo combination (seconds)	Specific reaction speed and movement coordination
FSKT-10s (Frequency Speed of Kick Test-10s)	Continuous kicking speed test over 10 s	Technical endurance and speed retention
FSKT-mult (Frequency Speed of Kick Test-mult)	Consecutive sets of kicking speed tests	Technical endurance and speed retention
CMJ (Countermovement jump)	Vertical jump height using a countermovement (cm)	Explosive and elastic lower-limb strength
VO_2_max (Maximal oxygen consumption)	The maximum volume of oxygen consumed per unit of time (ml/kg/min)	Efficiency of the aerobic metabolic system
HR_mean (Mean heart rate)	Average heart rate recorded during training	Overall training load
HR_max (Maximum heart rate)	Highest heart rate achieved during maximal exertion	Maximal capacity of the cardiopulmonary system
HR_peak (Heart rate peak)	Instantaneous peak heart rate recorded during a session	Physiological response to peak exercise intensity

#### 2.4.2 Exclusion criteria

Studies meeting any of the following conditions were excluded (see Table 3).

**Table 3:** Exclusion criteria based on the PICOS framework.

PICOS	Content
P (Patients/Problem)	Participants who were not taekwondo athletes, or athletes with existing sports injuries or medical conditions
I (Intervention)	Interventions involving substances explicitly banned by the World Anti-Doping Agency (WADA)
C (Comparison)	Control groups that received exercise training or nutritional supplementation
O (Outcomes)	Studies that did not report any of the following outcomes: TSAT, FSKT, CMJ, VO₂max, or HR parameters
S (Study design)	Studies that were not RCTs or randomized crossover trials; conference abstracts, review articles, irrelevant literature, or non-English publications

### 2.5 Data extraction and processing procedures

To ensure the methodological rigor of this study, a double-blind literature screening procedure was adopted and carried out independently by two reviewers. In the initial screening phase, the two reviewers independently screened the titles and abstracts to identify studies that either met the inclusion criteria or required further evaluation. Studies that were potentially eligible or presented uncertainties were forwarded to the full-text review stage. Subsequently, the selected full texts were independently assessed by two reviewers (CX and WZ) to determine whether they met the inclusion criteria for the systematic review and meta-analysis. In the event of disagreement between the two reviewers (CX and WZ) during the screening process, consensus was sought through discussion; if consensus could not be reached, a third reviewer (LL) was consulted to make the final decision.

The data extraction process was also conducted independently by two reviewers (CX and WZ) using a pre-designed Excel extraction sheet to ensure standardized documentation. Extracted data included: first author, year of publication, study design, participant characteristics (e.g., age, body weight, training experience, and sex), sample size, intervention type (exercise modality and nutritional supplement), duration and frequency of intervention, and reported performance outcomes.

Outcome data were extracted from each study, including TSAT (seconds), FSKT (repetitions), CMJ (centimeters), VO_2_max (mL/kg/min), and HR (beats per minute, bpm), along with corresponding means and standard deviations (SD), standard errors (SE), or 95% confidence intervals (95% CI). If a study reported only SE without SD, SD was calculated using the formula SD = SE × $\sqrt{n}$ (for paired/crossover designs, within-subject correlations were accounted for where available).

For studies with incomplete outcome reporting or graphical-only data, the original authors were contacted via email to request raw data. All extracted data were cross-verified by both reviewers to ensure accuracy and reliability.

### 2.6 Quality assessment and risk of bias evaluation

The Cochrane Risk of Bias tool (RoB 2.0), as recommended by the Cochrane Collaboration, was used to assess the methodological quality of the included studies. The assessment was independently performed by two reviewers (CX and WZ). RoB 2.0 domains include: randomization process; deviations from intended interventions; missing outcome data; measurement of the outcome; selection of the reported result. Each domain was categorized as having a “low risk,” “high risk,” or “some concerns” regarding bias. For each outcome, an overall risk of bias judgment was made following the RoB 2.0 algorithm: “low risk” if all domains were judged as low risk, “high risk” if at least one domain was judged as high risk, and “some concerns” if at least one domain raised some concerns but no domain was at high risk. Any discrepancies were resolved through discussion, with a third reviewer (LL) consulted when necessary.

### 2.7 Data analysis methods

The meta-analysis was performed using Review Manager (RevMan) and Stata 16.0 statistical software. The meta-analysis prioritized primary outcomes (TSAT, FSKT-10s, FSKT-mult); secondary outcomes (CMJ, VO_2_max, HR indices) were synthesized to contextualize sport-specific effects. Given the variation in measurement tools and units across the included studies, standardized mean difference (SMD) and corresponding 95% confidence intervals (95% CI) were adopted as the effect size metrics to facilitate comparability. SMD was calculated by dividing the difference in means between the intervention and control groups by the pooled standard deviation. A significance threshold of *p* < 0.05 was applied. The magnitude of the SMD was interpreted according to Cohen’s classification—pre-specified in the analysis plan—as trivial (SMD < 0.2), small (0.2 ≤ SMD < 0.5), moderate (0.5 ≤ SMD < 0.8), and large (SMD ≥ 0.8).

For multi-arm studies, we addressed potential dependencies between effect sizes through the following approaches: (1) extracting independent comparisons where each intervention group was separately compared with the control group; (2) using shared control group methods with sample size adjustments when appropriate to avoid double-counting of participants; (3) conducting sensitivity analyses excluding multi-arm studies to verify the robustness of results. Random-effects models were prespecified for primary analyses; fixed-effects models were used only in sensitivity analyses when heterogeneity was low (*p* ≥ 0.05 and *I*^2^ < 50%).

Heterogeneity among studies was assessed using the *I*^2^ statistic and *τ*^2^. When *p* ≥ 0.05 and *I*^2^ < 50%, results were considered to show low heterogeneity; otherwise, substantial heterogeneity (*p* < 0.05 or *I*^2^ ≥ 50%) prompted subgroup/sensitivity analyses. Subgroup variables included intervention duration (≤1 week vs. >1 week), intervention type, sex, study design (RCT vs. crossover), weight category (≤65 kg vs. >65 kg), and training experience (≤5 years vs. >5 years). Subgroup analyses were pre-specified and interpreted cautiously. For subgroups with fewer than five studies (or fewer than 150 total participants), estimates were reported descriptively without emphasizing *p*-values, and such findings were regarded as hypothesis-generating rather than confirmatory.

Random-effects meta-regression (REML) was planned only for outcomes with at least 10 effect sizes, using prespecified moderators including intervention type, duration, sex, study design, weight class, and training experience. Because most outcomes involved fewer than 10 contributing studies and CMJ exhibited negligible heterogeneity (*I*^2^ ≈ 0%), meta-regression was not performed or was limited to exploratory analyses that were not intended for confirmatory inference.

To characterize between-study variability, *a priori* subgroup analyses, *τ*^2^ estimates, and 95% prediction intervals (PI) were applied. When multiple effects were reported within a single study, potential dependency was minimized through shared-control adjustments and sensitivity analyses. Consideration was also given to the use of robust variance estimation, but this approach was not implemented due to the limited number of available studies.

In addition, when at least 10 studies were included for a given outcome, publication bias was preliminarily assessed by visual inspection of funnel plots generated using Stata 16.0, and by Egger’s test where appropriate; for outcomes with fewer than 10 studies, small-study effects were interpreted qualitatively due to limited power. Sensitivity analysis was also conducted by sequentially removing individual studies to observe changes in the pooled effect size, thereby verifying the robustness and stability of the results and strengthening the evidence supporting the conclusions.

## 3 Results

### 3.1 Literature search results

A total of 993 relevant records were identified through a systematic search of three major electronic databases: Web of Science (WoS), PubMed, and Scopus. Specifically, 538 records were retrieved from WoS, 154 from PubMed, and 301 from Scopus. Prior to the initial screening, 376 duplicate records were removed, resulting in 617 unique records eligible for title and abstract screening.

During this initial screening stage, 535 records were excluded based on titles and abstracts due to irrelevance to the research topic, leaving 82 articles for full-text assessment.

After full-text review, 51 articles were excluded for the following reasons: 19 did not report relevant outcome measures; 4 had unavailable full texts; 22 did not meet the required study design criteria; 3 provided incomplete post-intervention data; and 3 were non-English publications.

**Figure 1:**
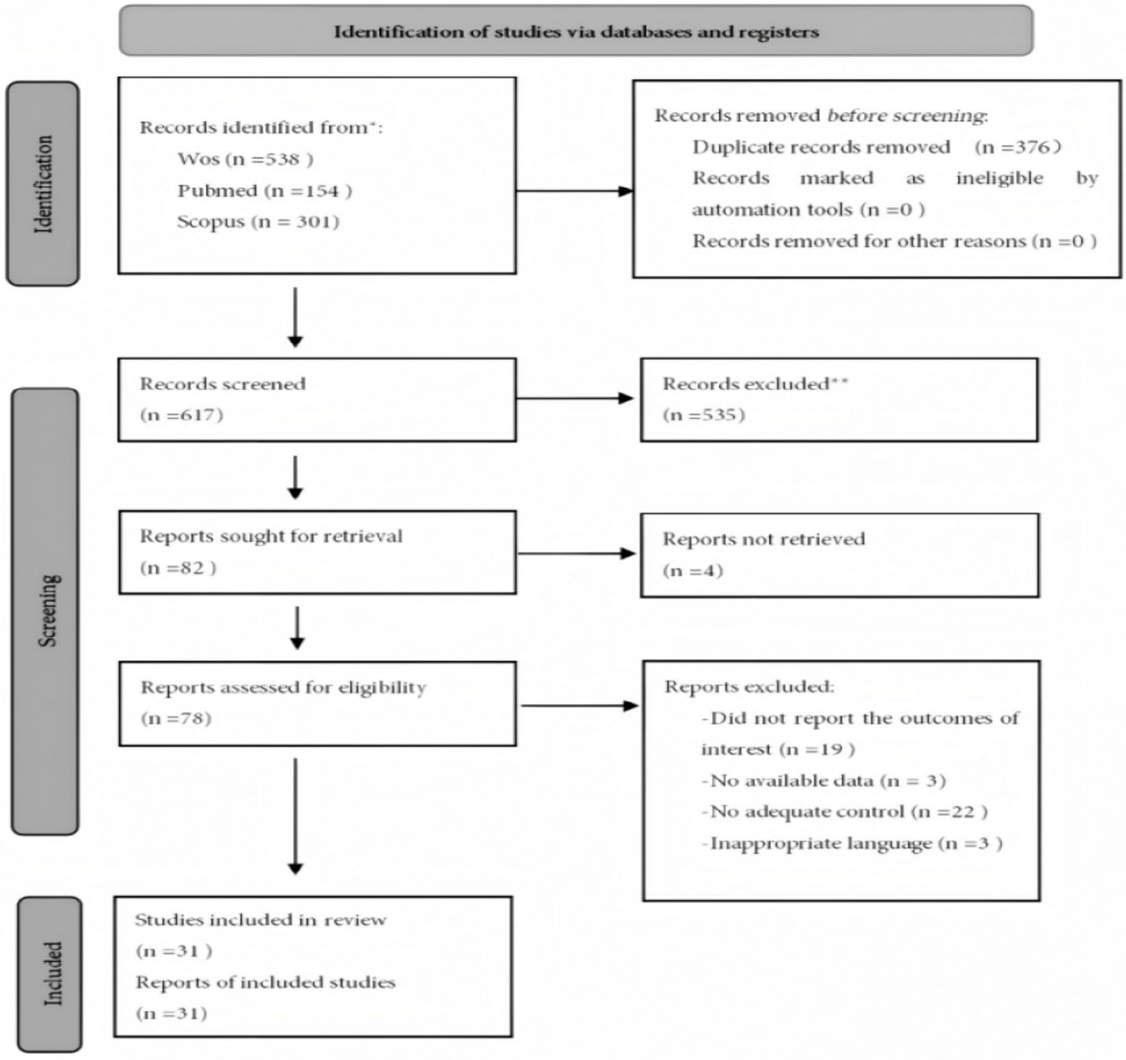
PRISMA flow diagram of the literature search and screening process.

Following a rigorous and systematic screening process, a total of 31 studies met the inclusion criteria and were included in the subsequent analyses. A detailed flow diagram of the literature selection process is presented in Figure 1.

### 3.2 Study characteristics and methodological quality assessment

#### 3.2.1 Study design

Among the 31 studies included in this meta-analysis, 16 were randomized controlled trials (RCTs) and 15 were randomized crossover trials. Of these, 3 studies employed a four-arm design, 5 employed a three-arm design, and the remaining 23 were two-arm designs (27, 34–63). Detailed study designs and control conditions are presented in Table 4.

**Table 4:** Intervention characteristics.

Number	Study	Sample (size, sex)	Age (years)	Duration	Outcome	Interventions
1	Aravena Tapia et al. (34)	12 (2 females, 10 males)	20.8 ± 5.3	4 weeks	FSKT-mult	Usual training, high-intensity interval training (HIIT)
2	Arjang et al. (35)	20	9–18	<1 week	TSAT	Usual training, proprioceptive neuromuscular facilitation (PNF)
3	Carazo-Vargas and Moncada-Jiménez (36)	12 (4 females, 8 males)	20.7 ± 3.1	13 weeks	CMJ	Usual training, reducing training volume
4	Chen et al. (37)	15 (males)	20.63 ± 1.18	<1 week	CMJ, HR_max, HR_mean, FSKT-mult	Usual training, vibration foam rolling (VR), double VR for the weaker leg (double VR)
5	Chiu et al. (38)	13	20.9 ± 1.0	<1 week	FSKT-mult	Usual training, carbohydrate mouth rinsing (CMR)
6	da Silva Santos et al. (39)	11	20.3 ± 5.2	<1 week	CMJ, FSKT-10s	Usual training, resistance training (RT), plyometric training (PT), resistance training and plyometric training (RT + PT)
7	Delleli et al. (42)	16 (males)	18.25 ± 0.75	1.5 months	TSAT, FSKT-10s, FSKT-mult	Usual training, caffeine ingestion
8	Delleli et al. (40)	16 (females)	17.69 ± 0.60	5 weeks	TSAT, FSKT-10s, FSKT-mult	Usual training, caffeine ingestion
9	Delleli et al. (41)	16 (females)	17–19	3 weeks	HR_peak, HR_mean	Usual training, caffeine ingestion
10	Gaamouri et al. (44)	23 (11 females, 12 males)	21.9 ± 1.2	6 weeks	HR_peak	Usual training, polyphenol supplementation
11	Gaamouri et al. (43)	22 (10 females, 12 males)	21.9 ± 1.2	6 weeks	FSKT-mult	Usual training, polyphenol supplementation
12	Hassan et al. (45)	30	18.82 ± 0.69	8 weeks	VO_2_ max	Usual training, high-intensity interval training with breathing mask (HIIT-M)
13	Jung et al. (46)	35 (14 females, 21 males)	20.1 ± 0.15	4 weeks	CMJ	Usual training, vitamin D supplementation
14	Kavcı et al. (47)	16	19.06 ± 0.96	<1 week	VO_2_ max	Usual training, nitrate supplements, L-arginine supplements, nitrate and L-arginine supplements
15	Koc et al. (48)	42 (males)	19.1 ± 1.76	8 weeks	VO_2_ max	Usual training, chronic inspiratory training group (CRG), acute inspiratory training group (ARG)
16	Lopes-Silva et al. (49)	10 (males)	21 ± 4	<1 week	HR_peak, HR_mean	Usual training, caffeine ingestion
17	Lopes-Silva et al. (50)	9 (males)	19.4 ± 2.2	<1 week	HR_mean	Usual training, sodium bicarbonate supplementation
18	Messaoudi et al. (51)	16 (males)	19.94 ± 1.12	<1 week	TSAT, FSKT-10s, FSKT-mult	Usual training, conditioning activity
19	Miraftabi et al. (27)	8 (males)	20 ± 4	<1 week	HR_peak, HR_mean	Usual training, nitrate (NO_3_^−^)-rich beetroot juice (BJ)
20	Ojeda-Aravena et al. (52)	16 (5 females, 11 males)	19.5 ± 4.6	4 weeks	CMJ, TSAT, FSKT-mult	Usual training, high-intensity interval training with specific techniques (HIITTS)
21	Ou et al. (53)	17 (males)	19.65 ± 1.41	<1 week	FSKT-mult	Usual training, ischemic preconditioning (IPC)
22	Ouergui et al. (55)	36 (9 females, 27 males)	16 ± 1	4 weeks	CMJ, TSAT	Usual training, repeated sprints training (RST), repeated high-intensity techniques training (RTT)
23	Ouergui et al. (57)	32 (males)	15 ± 1	8 weeks	CMJ, TSAT, VO_2_ max	Usual training, different area sizes
24	Ouergui et al. (54)	20 (10 females, 10 males)	17.5 ± 0.7	<1 week	TSAT, FSKT-10s, FSKT-mult	Usual training, caffeine ingestion, conditioning activity
25	Ouergui et al. (58)	27 (13 females, 14 males)	16 ± 1	<1 week	CMJ, TSAT, FSKT-10s, FSKT-mult	Usual training, plyometric training (PT), repeated high-intensity techniques training (RTT)
26	Ouergui et al. (56)	52 (26 females, 26 males)	17.75 ± 0.98	<1 week	TSAT, FSKT-10s, FSKT-mult	Usual training, caffeine ingestion
27	Riera et al. (59)	20 (males)	21.4 ± 6.3	30 days	HR_max	Usual training, nucleotide formulation
28	Santos et al. (60)	10 (males)	24.9 ± 7.3	<1 week	HR_peak, HR_mean	Usual training, caffeine ingestion
29	Seo et al. (61)	47 (males)	16.7 ± 0.84	4 weeks	HR_max, VO_2_ max	Usual training, high-intensity interval training (HIIT)
30	Song and Sheykhlouvand (62)	30 (males)	19.8 ± 1.3	6 weeks	CMJ, TSAT, VO_2_ max	Usual training, high-intensity interval training with specific techniques (HIITTS), high-intensity interval training in the form of repeated sprints (HIITRS)
31	Wang and Song (63)	90 (69 females, 21 males)	21.18 ± 1.67	2 months	VO_2_ max	Usual training, Fufang Ejiaojiang

#### 3.2.2 Participant characteristics

A total of 739 taekwondo athletes were included, with ages ranging from 15 to 24.9 years. Regarding sex distribution, 13 studies enrolled only male athletes (*n* = 272), 2 enrolled only female athletes (*n* = 32), and 11 included both male (*n* = 172) and female athletes (*n* = 173). The remaining 5 studies did not specify the sex of participants (*n* = 90).

#### 3.2.3 Intervention measures

Of the 31 included studies, 15 investigated exercise-only interventions, incorporating 18 different types of exercise strategies (see Appendix 2 for details). Another 15 studies involved nutrition-only interventions, utilizing 11 different types of supplements (see Appendix 3 for details). One study implemented both exercise and nutritional supplementation simultaneously.

#### 3.2.4 Outcome measures

All included studies reported at least one of the following primary outcome measures: TSAT, FSKT-10s, FSKT-mult, CMJ, VO_2_max, and HR parameters (HR_mean, HR_max, and HR_peak).

Among studies on exercise training, 9 reported TSAT, 7 reported FSKT-10s, 9 reported FSKT-mult, 14 reported CMJ, 7 reported VO_2_max, 2 reported HR_mean, 3 reported HR_max, and none reported HR_peak.

Among studies on nutritional supplementation, 4 reported TSAT, 4 reported FSKT-10s, 6 reported FSKT-mult, 1 reported CMJ, 4 reported VO_2_max, 5 reported HR_mean, 1 reported HR_max, and 5 reported HR_peak.

### 3.3 Risk of bias assessment

According to the RoB 2.0 risk of bias assessment, the overall methodological quality of the 31 randomized controlled trials included in this study was relatively high. Among the six core evaluation domains, the randomization process performed best, with approximately 90% of studies judged as low risk and only 10% rated as having some concerns. Missing outcome data and selective reporting were also well controlled, with more than 85% of studies classified as low risk. By contrast, the measurement of outcome domain was relatively weaker, with about 75% rated as low risk and 25% as having some concerns. For deviations from intended interventions, 80% of studies were rated low risk, 15% had some concerns, and 5% were rated high risk. Overall, 80% of the included studies were judged as having low risk of bias, 20% as having some concerns, and none as high risk.

At the study level, the vast majority of trials demonstrated low risk (green) across key methodological domains, indicating generally acceptable design and implementation quality. The primary methodological concerns were concentrated in the domains of outcome measurement and deviations from intended interventions, where several studies were rated as “unclear risk” (yellow) due to insufficient objectivity in measurement methods or incomplete blinding implementation. Notably, no study was rated as high risk (red) in any domain, which strengthens the credibility of the meta-analysis findings and reflects the strict inclusion criteria applied, effectively minimizing the entry of low-quality studies.

**Figure 2:**
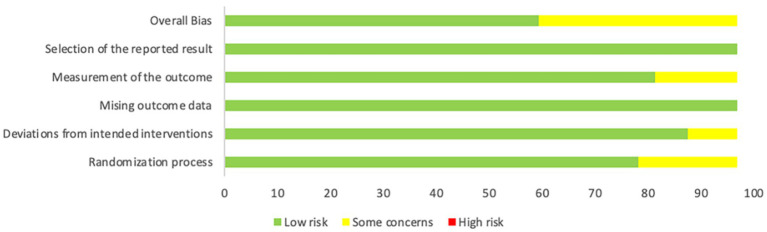
Overall distribution of risk of bias across included studies.

**Figure 3:**
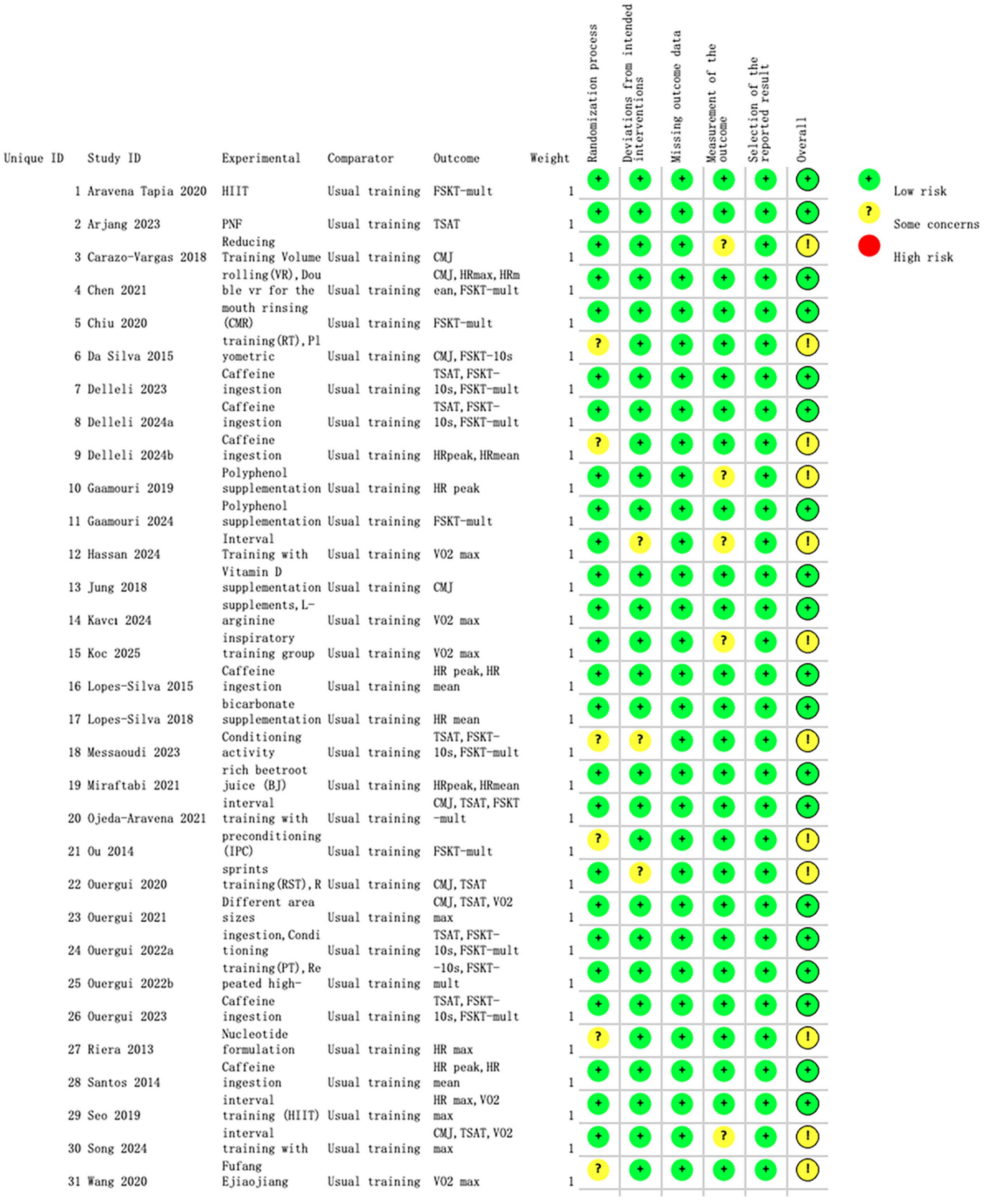
Summary of risk of bias for each included study (green plus sign = low risk; yellow question mark = unclear risk; red minus sign = high risk).

The results of the RoB 2.0 assessment are illustrated in Figures 2, 3.

### 3.4 Meta-analysis results

#### 3.4.1 Effects of exercise trainings

##### 3.4.1.1 Improvement in Taekwondo-Specific Agility Test

**Figure 4:**
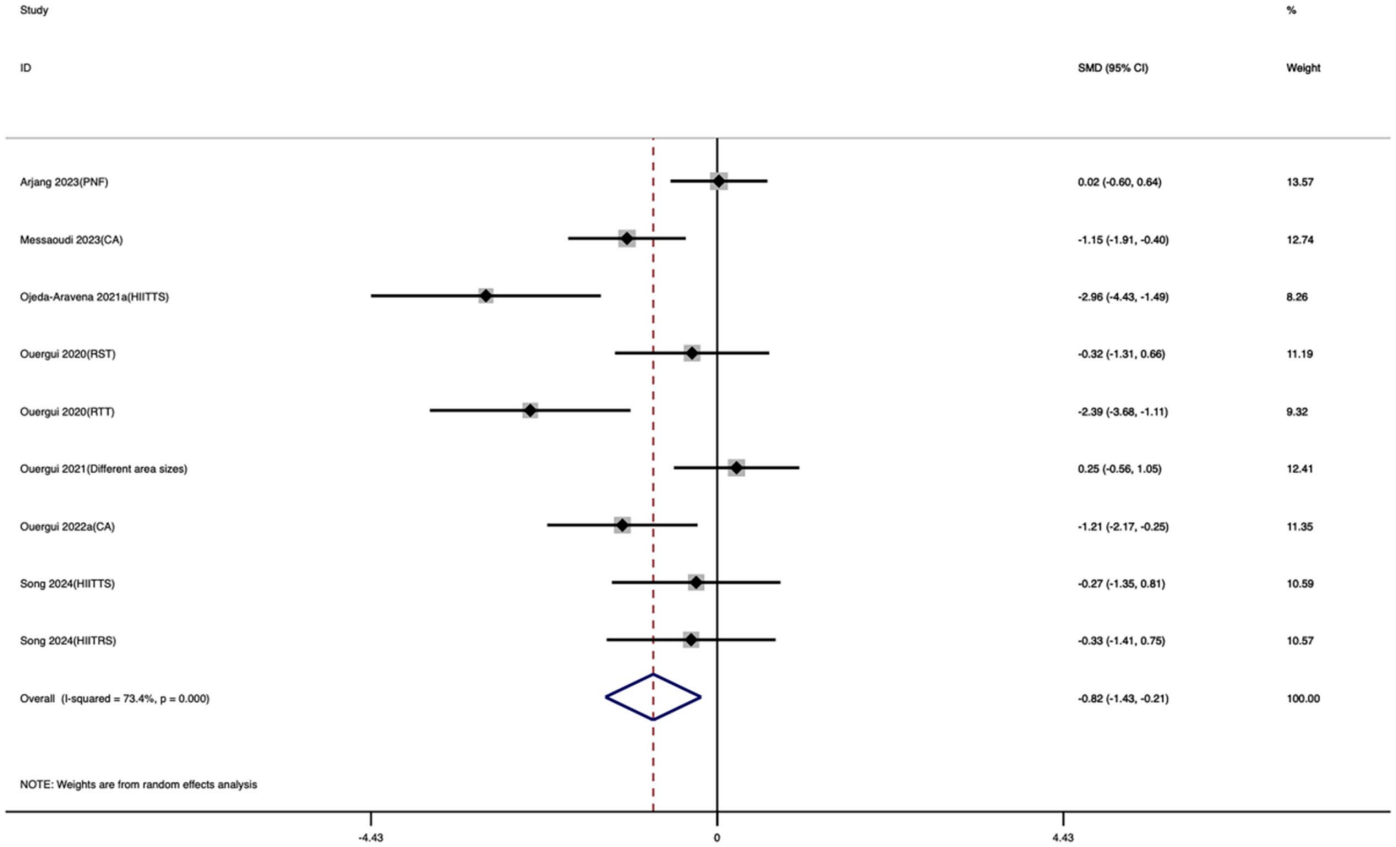
Forest plot of the effect of exercise training on TSAT.

A total of 9 studies were included in this meta-analysis to evaluate the effect of exercise training on TSAT. As shown in Figure 4, the meta-analysis results indicated that compared with the control group, exercise training significantly reduced the time required to complete TSAT. The standardized mean difference demonstrated a large and statistically significant effect (SMD = −0.82; 95% CI: −1.43 to −0.21; *I*^2^ = 73.4%; *p* = 0.009).

##### 3.4.1.2 Improvement in 10-s Frequency Speed of Kick Test

**Figure 5:**
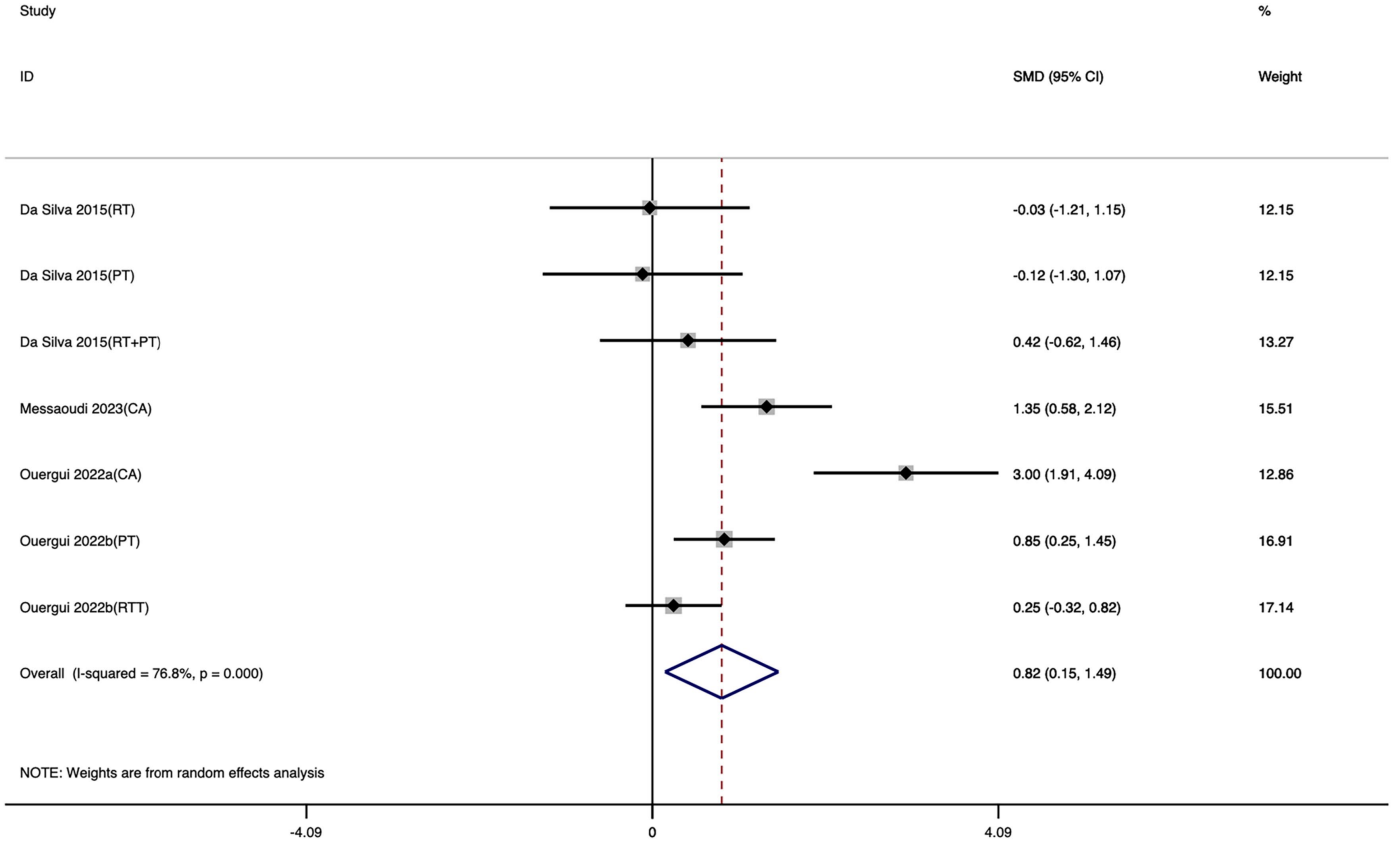
Forest plot of the effect of exercise training on FSKT-10s.

Seven studies were included in the meta-analysis evaluating the effect of exercise training on FSKT-10s. As shown in Figure 5, taekwondo athletes who received exercise training performed significantly better in FSKT-10s compared with the control group, as reflected by a notable increase in the number of kicks. The effect size was moderate (SMD = 0.82; 95% CI: 0.15 to 1.49; *I*^2^ = 76.8%; *p* = 0.016).

##### 3.4.1.3 Improvement in multiple-bout Frequency Speed of Kick Test

**Figure 6:**
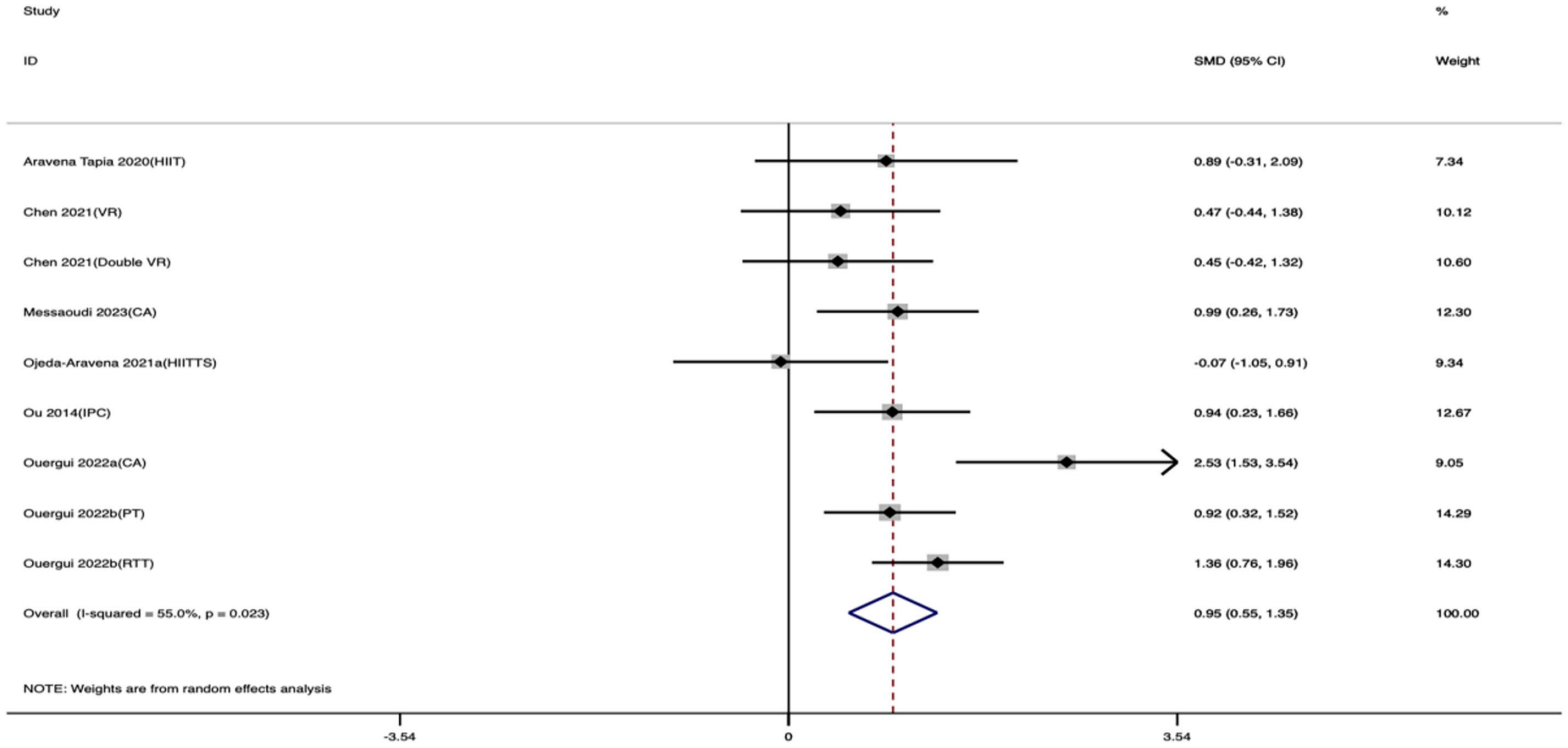
Forest plot of the effect of exercise training on FSKT-mult.

Nine studies were included to evaluate the effect of exercise training on FSKT-mult. As shown in Figure 6, taekwondo athletes who received exercise training performed significantly better than controls, as evidenced by a clear increase in the number of kicks. The standardized mean difference revealed a large and statistically significant effect (SMD = 0.95; 95% CI: 0.55 to 1.35; *I*^2^ = 55%; *p* < 0.001).

##### 3.4.1.4 Improvement in countermovement jump

**Figure 7:**
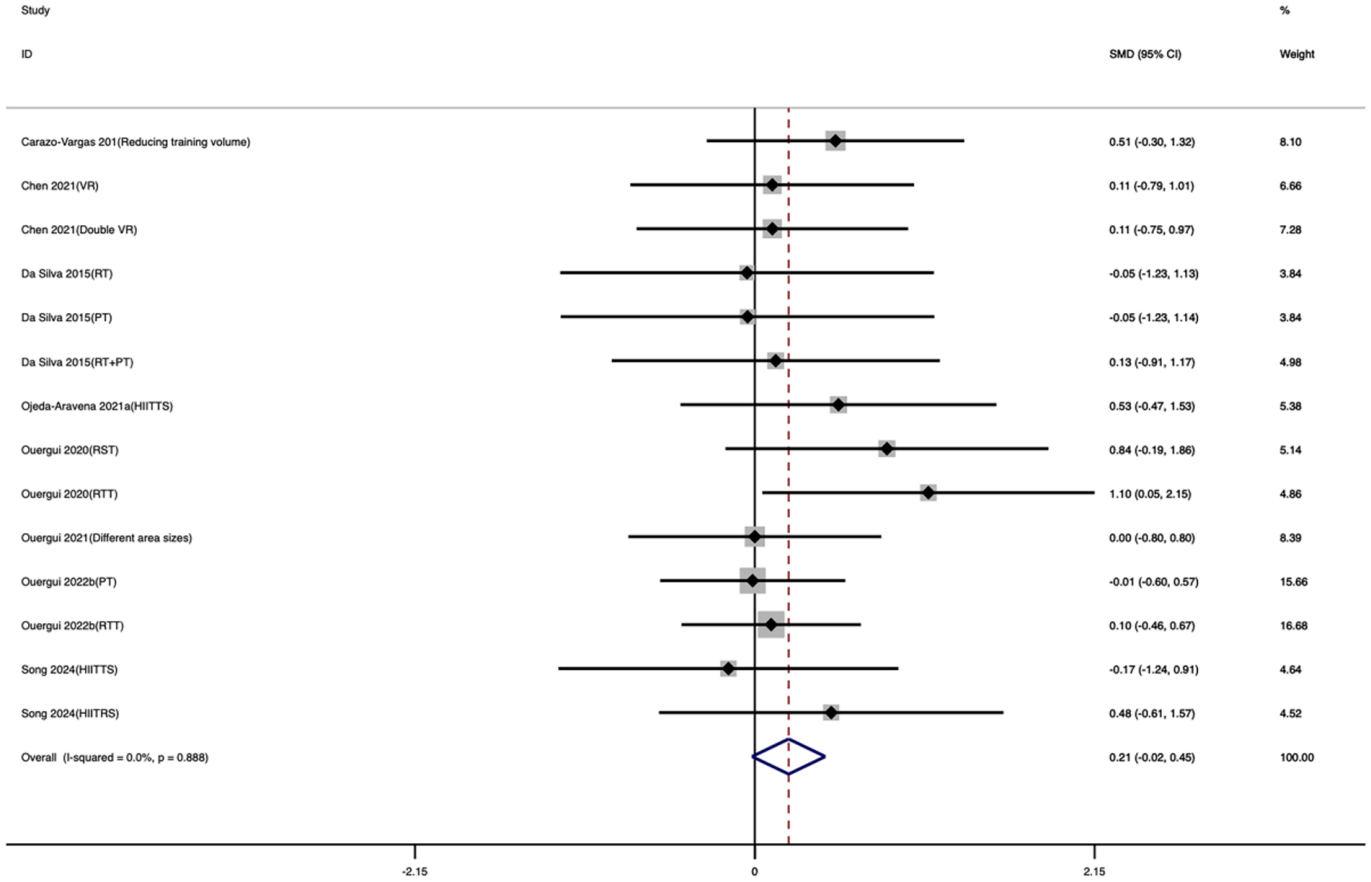
Forest plot of the effect of exercise training on CMJ.

Fourteen studies were included in the CMJ meta-analysis. As shown in Figure 7, compared with the control group, exercise training did not produce a statistically significant improvement in CMJ. Only a slight, non-significant trend toward enhancement was observed (SMD = 0.21; 95% CI: −0.02 to 0.45; *I*^2^ = 0%; *p* = 0.07). The heterogeneity across studies was minimal, indicating a high level of result consistency.

##### 3.4.1.5 Improvement in maximal oxygen uptake

**Figure 8:**
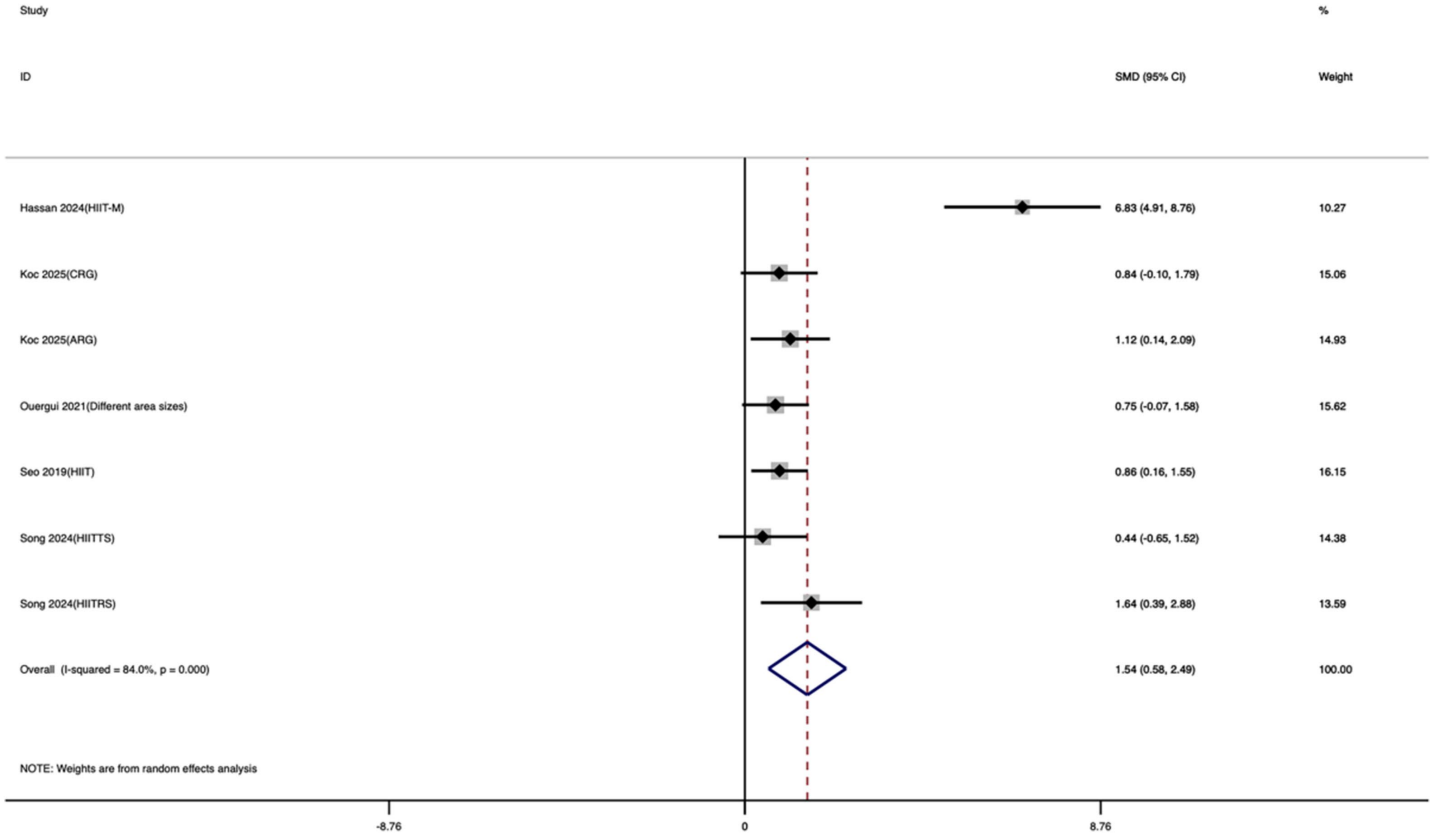
Forest plot of the effect of exercise training on VO_2_max.

Seven studies were included to evaluate the effect of exercise training on VO_2_max. As shown in Figure 8, athletes who underwent exercise training demonstrated a significantly greater improvement in VO_2_max compared with controls, with a large and statistically significant effect size (SMD = 1.54; 95% CI: 0.58 to 2.49; *I*^2^ = 84%; *p* = 0.002). Although there was substantial heterogeneity among studies—reducing the consistency of results—the overall findings clearly demonstrated that exercise training significantly enhanced aerobic metabolic capacity in taekwondo athletes.

##### 3.4.1.6 Improvement in maximum heart rate

**Figure 9:**
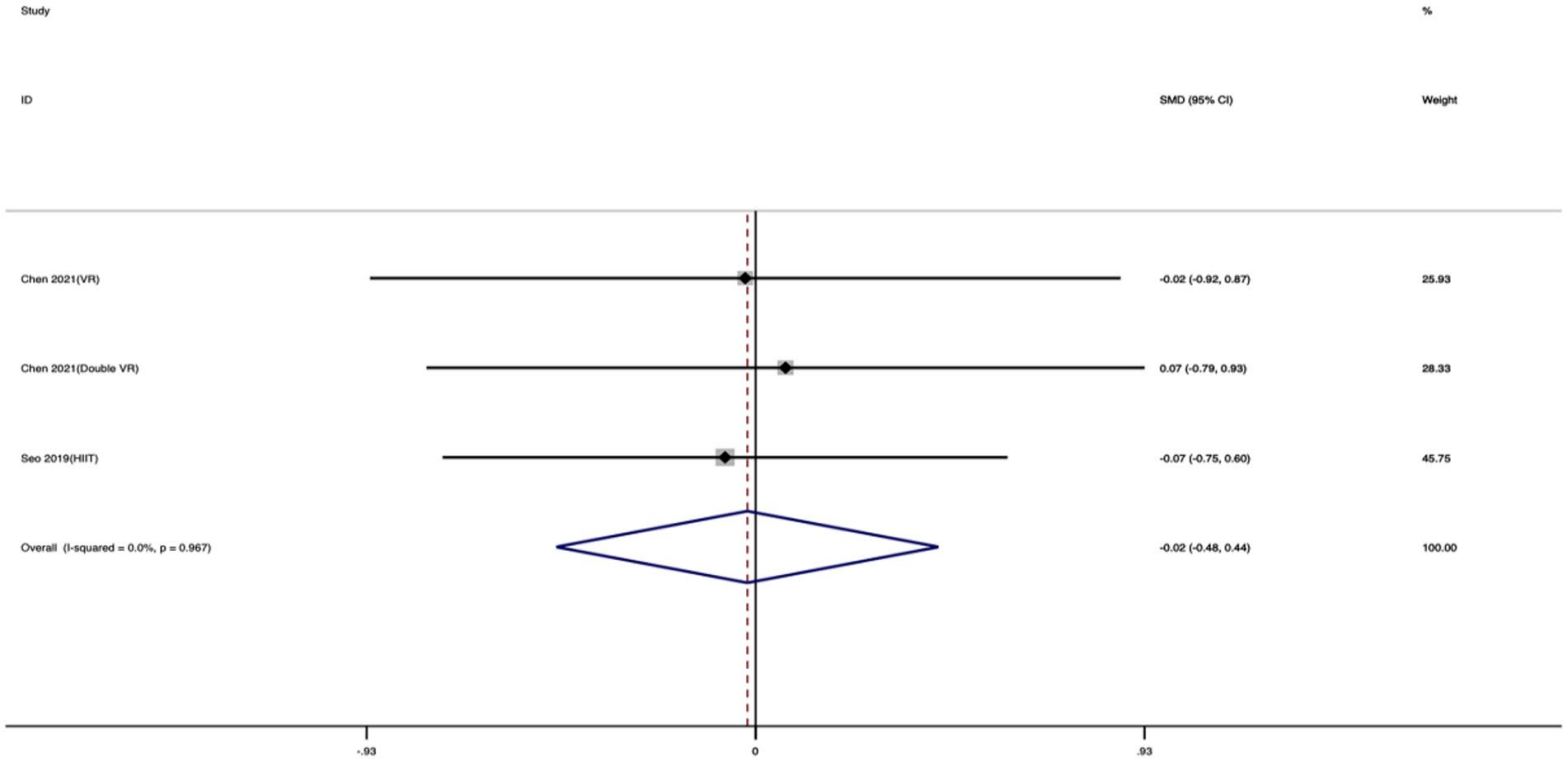
Forest plot of the effect of exercise training on HR_max.

Five studies were included in the HR_max meta-analysis. As shown in Figure 9, no significant between-group difference was observed in HR_max between the exercise and control groups. The standardized mean difference indicated a negligible and statistically non-significant effect (SMD = −0.02; 95% CI: −0.48 to 0.44; *I*^2^ = 0%; *p* = 0.993), with no substantial heterogeneity across studies.

#### 3.4.2 Effects of nutritional supplementations

##### 3.4.2.1 Improvement in Taekwondo-Specific Agility Test

**Figure 10:**
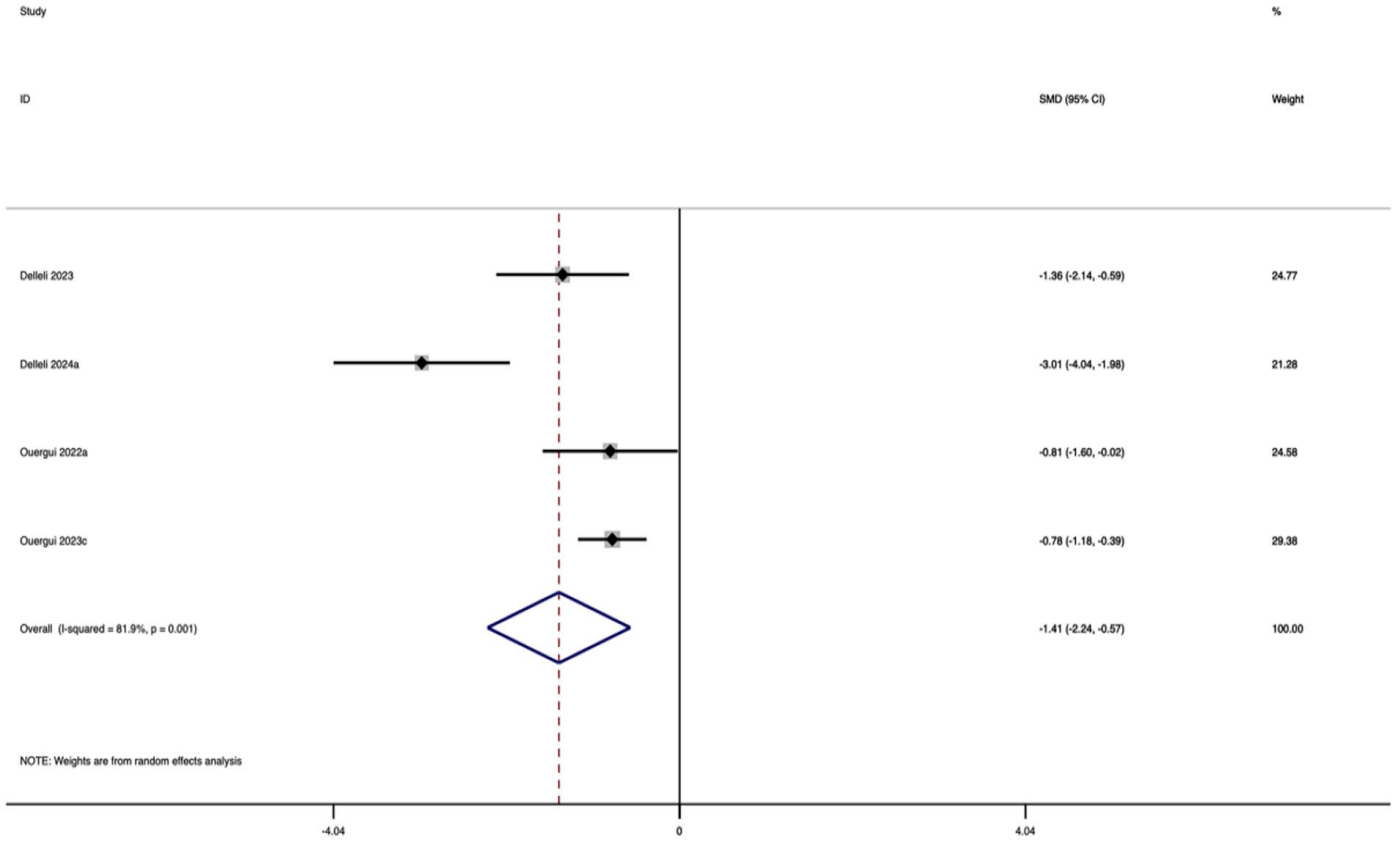
Forest plot of the effect of nutritional supplementation on TSAT.

A total of four studies were included in this meta-analysis to evaluate the effect of caffeine supplementation on TSAT. As shown in Figure 10, the results indicated that, compared with the control group, the caffeine group exhibited a significantly shorter TSAT completion time, reflecting a notable improvement in agility performance. The effect size was large and statistically significant (SMD = −1.41; 95% CI: −2.24 to −0.57; *I*^2^ = 81.9%; *p* = 0.001). Substantial heterogeneity was observed, suggesting that results should be interpreted with caution.

##### 3.4.2.2 Improvement in 10-s Frequency Speed of Kick Test

**Figure 11:**
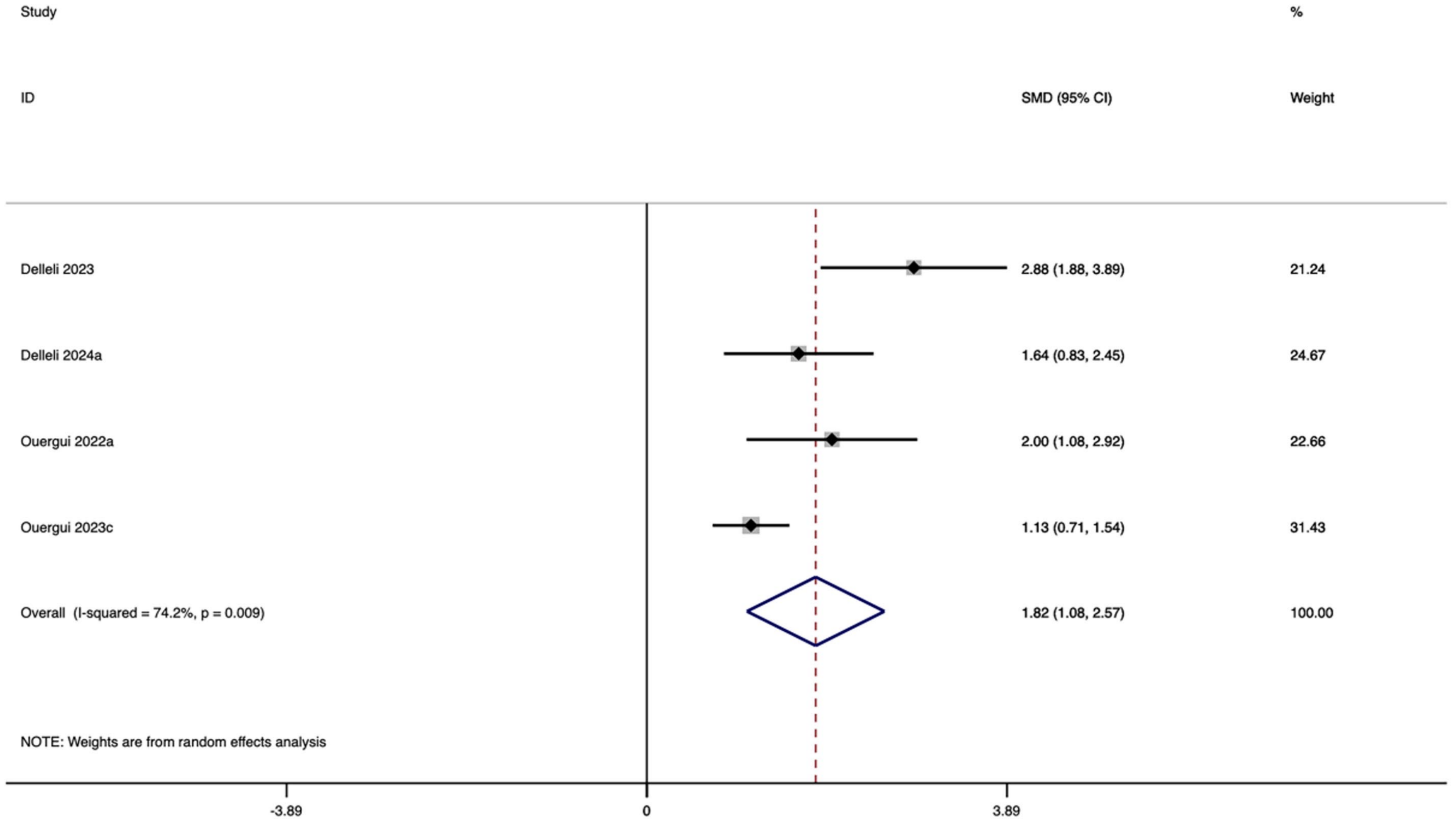
Forest plot of the effect of nutritional supplementation on FSKT-10s.

Four studies were included in the meta-analysis evaluating the effect of caffeine supplementation on FSKT-10s. As shown in Figure 11, caffeine intake significantly increased the number of kicks performed during FSKT-10s compared with the control group. The effect size was large and statistically significant (SMD = 1.82; 95% CI: 1.08 to 2.57; *I*^2^ = 74.2%; *p* < 0.001), although moderate heterogeneity was present.

##### 3.4.2.3 Improvement in multiple-bout Frequency Speed of Kick Test

**Figure 12:**
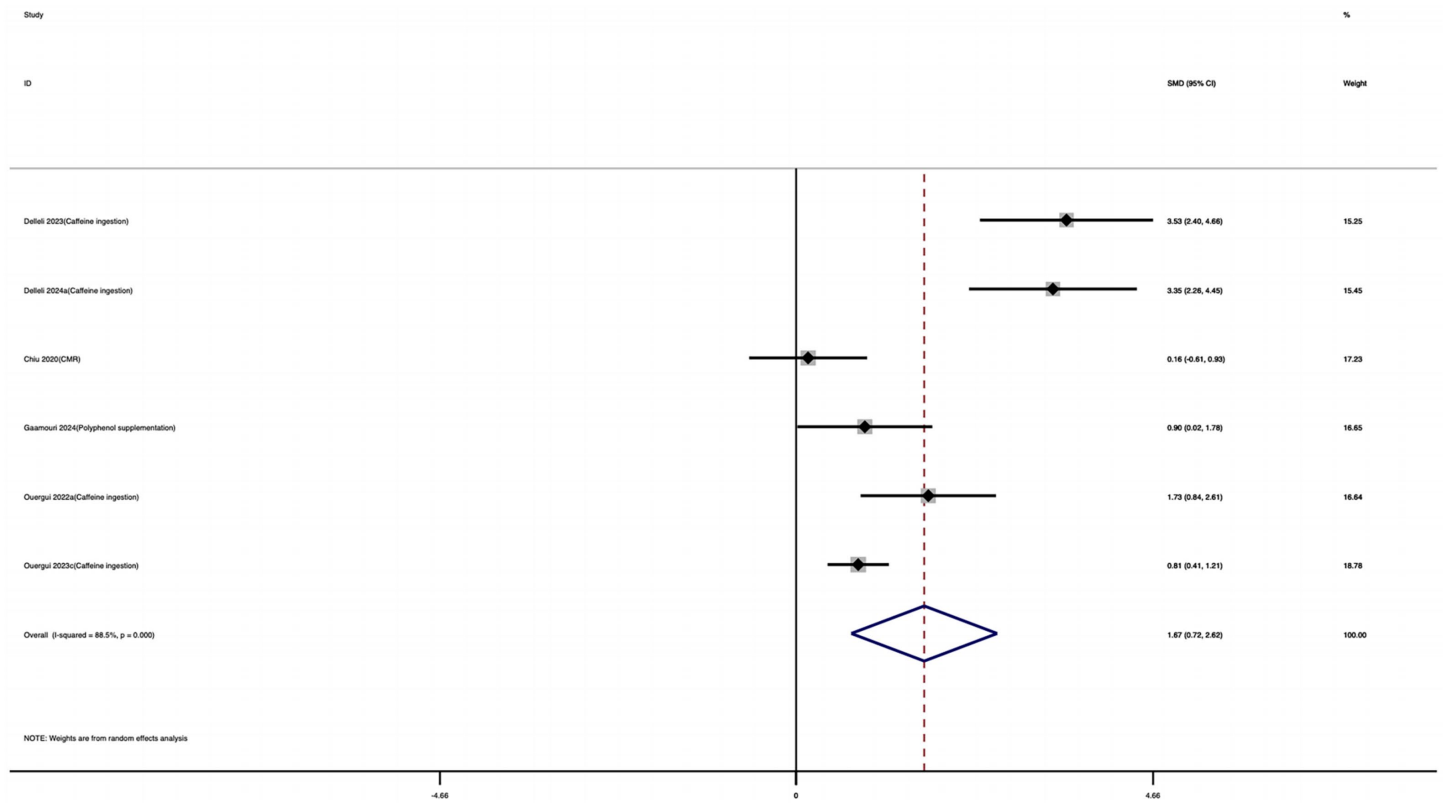
Forest plot of the effect of nutritional supplementation on FSKT-mult.

Six studies were included in this meta-analysis to evaluate the effect of nutritional supplementation on FSKT-mult. As shown in Figure 12, athletes who received nutritional supplementation performed significantly better than those in the control group, as indicated by a substantial increase in the number of kicks. The effect size was large and statistically significant (SMD = 1.67; 95% CI: 0.72 to 2.62; *I*^2^ = 88.5%; *p* = 0.001). High heterogeneity was observed, indicating variability among study designs and supplementation protocols.

##### 3.4.2.4 Improvement in maximal oxygen uptake

**Figure 13:**
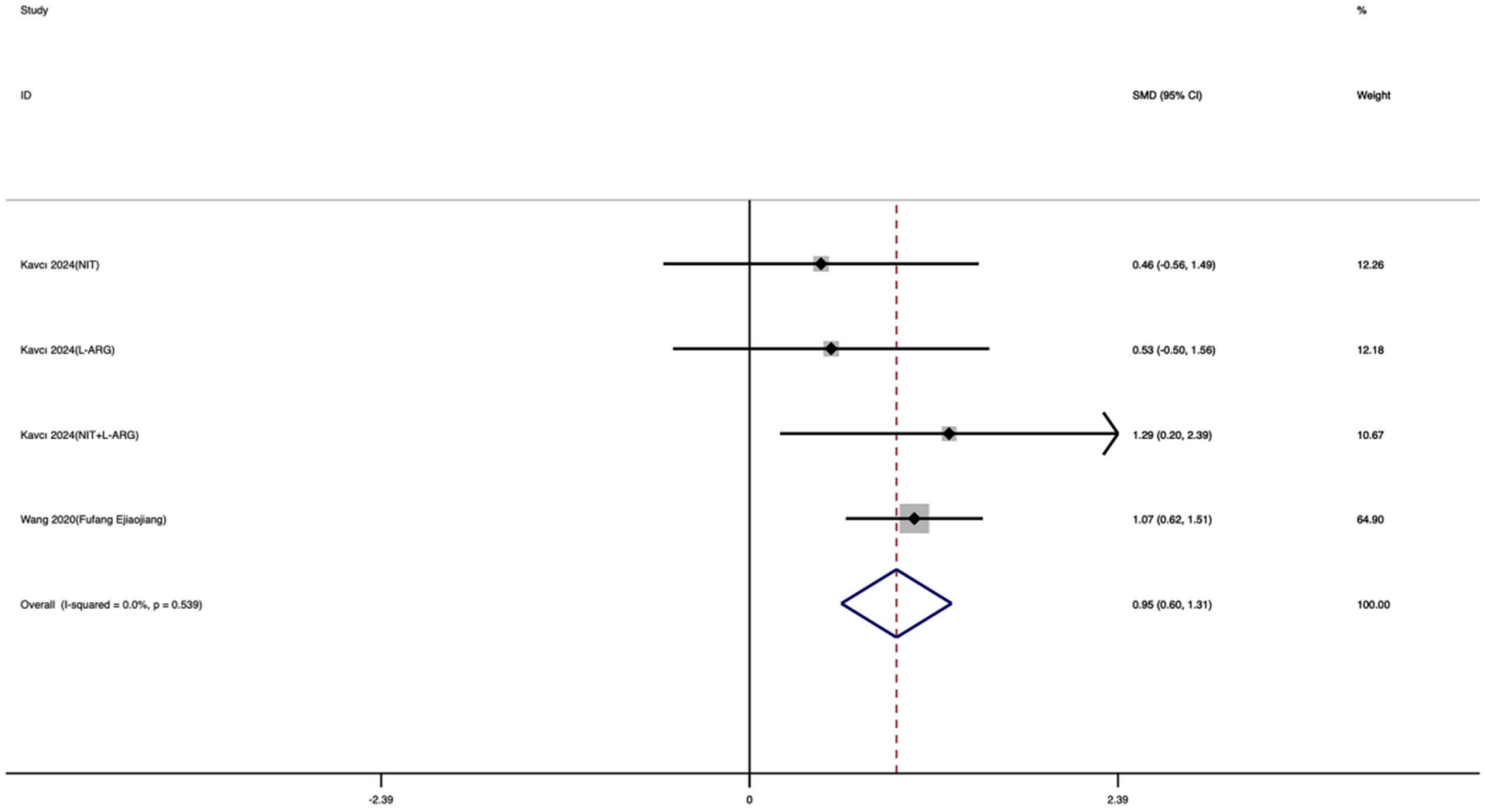
Forest plot of the effect of nutritional supplementation on VO_2_max.

Four studies were included to assess the effect of nutritional supplementation on VO_2_max. As shown in Figure 13, taekwondo athletes receiving nutritional supplementation demonstrated significantly greater improvements in VO_2_max compared with the control group. The effect size was large and statistically significant (SMD = 0.95; 95% CI: 0.60 to 1.31; *I*^2^ = 0%; *p* < 0.001), indicating a consistent enhancement of aerobic capacity across studies.

##### 3.4.2.5 Improvement in mean heart rate

**Figure 14:**
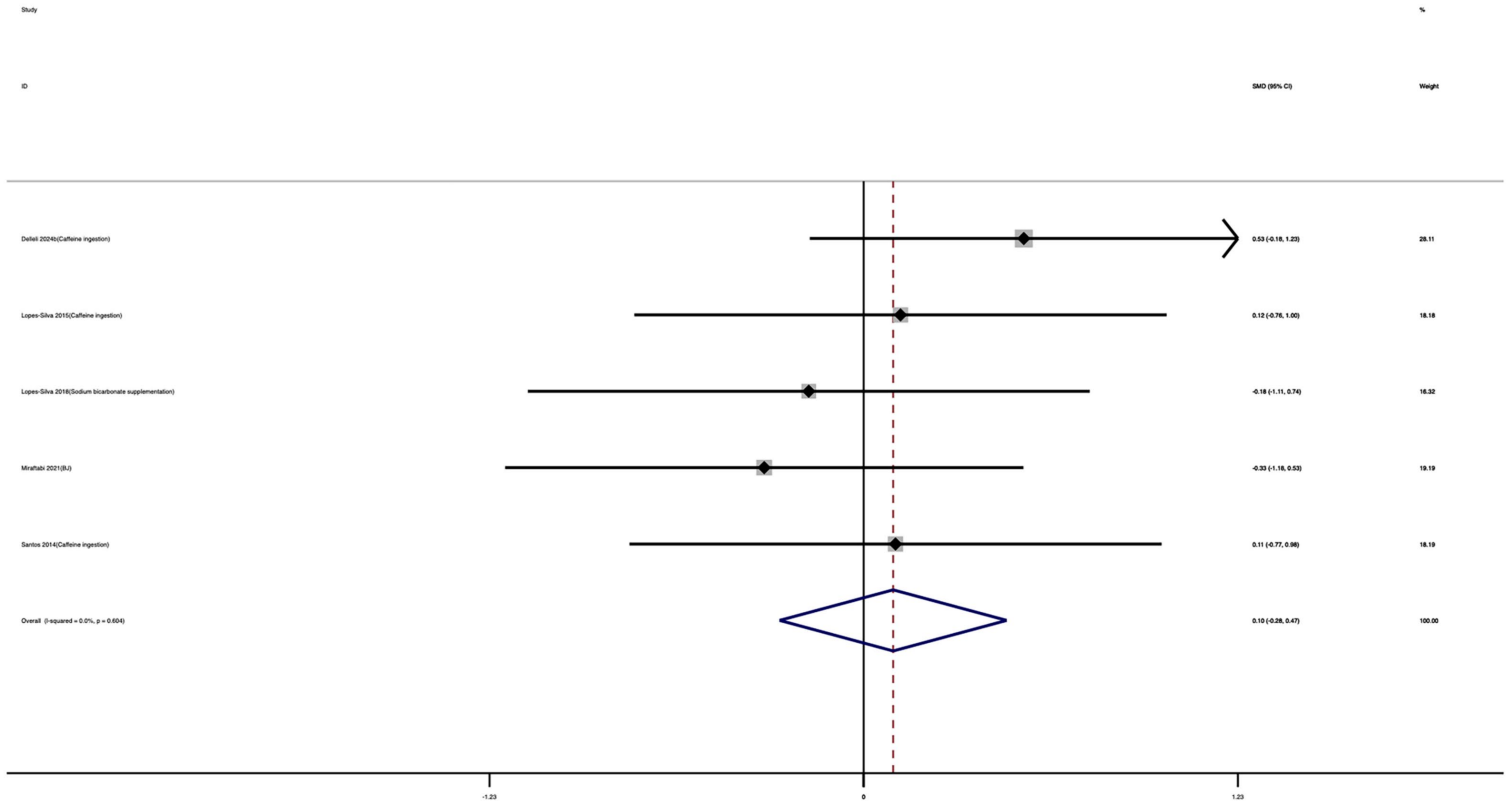
Forest plot showing the effect of nutritional supplementation on HR_mean.

Five studies were included to explore the effect of nutritional supplementation on HR_mean. As shown in Figure 14, there was no significant difference between the supplementation and control groups. The effect size was negligible and not statistically significant (SMD = 0.10; 95% CI: −0.28 to 0.47; *I*^2^ = 0%; *p* = 0.611), with no heterogeneity detected.

##### 3.4.2.6 Improvement in peak heart rate

**Figure 15:**
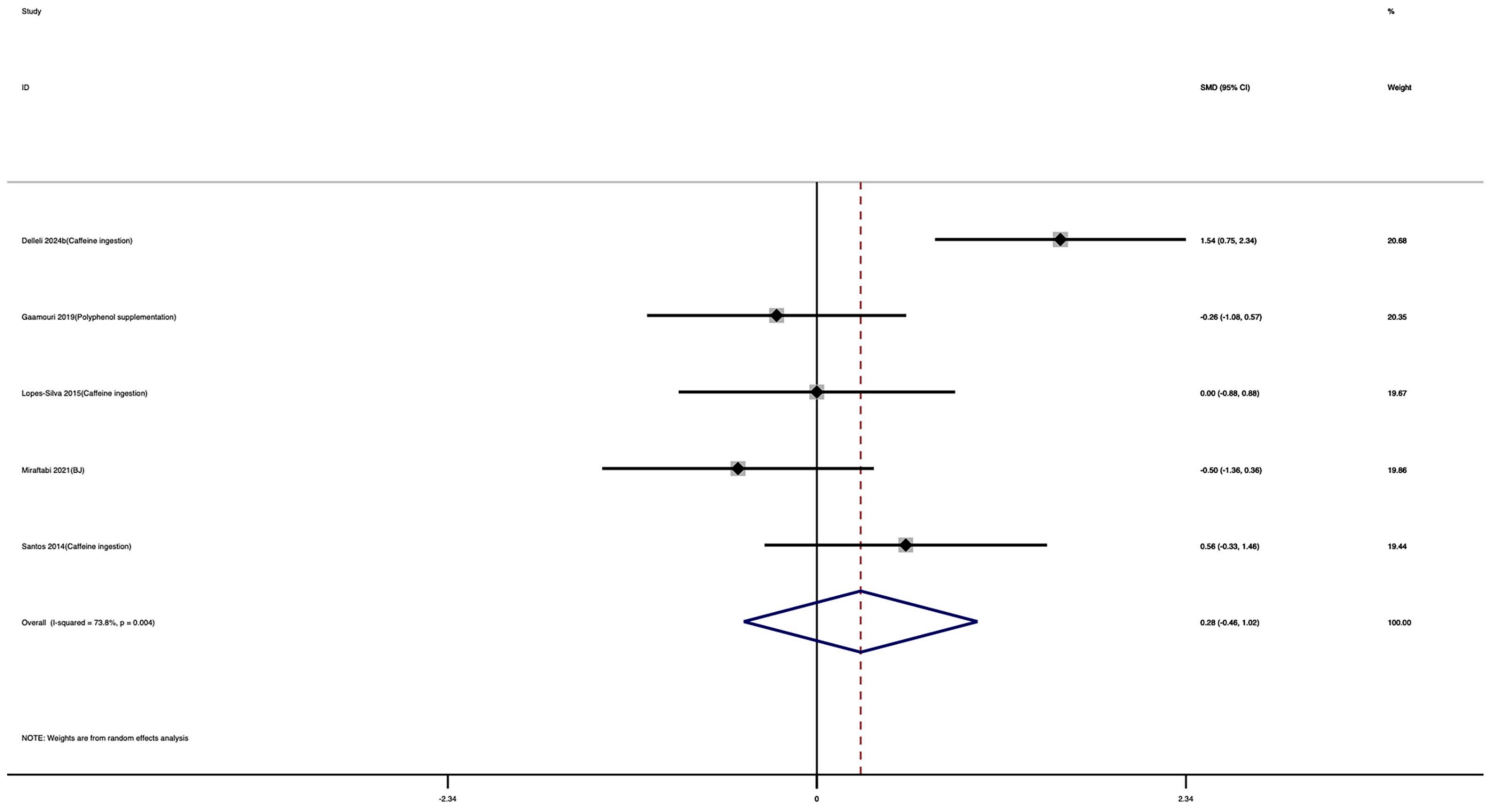
Forest plot showing the effect of nutritional supplementation on HR_peak.

Five studies were included in the meta-analysis of HR_peak. As shown in Figure 15, nutritional supplementation did not significantly affect HR_peak compared with the control group. The effect size was small and not statistically significant (SMD = 0.28; 95% CI: −0.46 to 1.02; *I*^2^ = 73.8%; *p* = 0.463). Considerable heterogeneity was observed across studies, which may reflect variations in supplement type, dosage, and assessment protocols.

### 3.5 Subgroup analysis results

Given the substantial heterogeneity identified in the overall analysis, subgroup analyses were conducted to explore potential sources of heterogeneity. Subgroups were categorized based on intervention duration, type of exercise intervention, sex, study design, body weight, and training experience.

#### 3.5.1 Subgroup analysis of exercise trainings

##### 3.5.1.1 TSAT

A detailed subgroup analysis was conducted to examine the effects of exercise training on TSAT (see Table 5).

**Table 5:** Subgroup analysis of exercise training.

Outcome	Covariate	Group	SMD	Lower 95% CI	Upper 95% CI	*p*	Heterogeneity (*I*^2^)	Regression
TSAT		9	−0.82	−1.43	−0.21	0.009	73.4	
	Intervention duration							0.260
	≤1 week	3	−0.72	−1.53	0.09	0.082	73.4	
	>1 week	6	−0.9	−1.84	0.04	0.060	77.8	
	Type of intervention							0.358
	Conditioning activity	2	−1.14	−1.69	−0.59	<0.001	0	
	HIIT	3	−1.11	−2.65	0.43	0.158	79.8	
	Repeated training	2	−1.31	−3.34	0.71	0.204	84.1	
	Others	2	0.11	−0.38	0.60	0.670	0	
	Sex							0.217
	Male	4	−0.4	−1.07	0.27	0.241	53.3	
	Female and male	5	−1.23	−2.25	−0.21	0.018	82.2	
	Study design							0.300
	RCT	7	−0.72	−1.49	0.04	0.063	76.3	
	Crossover	2	−1.14	−1.69	−0.59	<0.001	0	
	Body weight							0.292
	≤65 kg	5	−1.2	−2.28	−0.13	0.028	81.9	
	>65 kg	3	−0.11	−0.59	0.38	0.666	0	
	Unknown	1	−1.15	−1.91	−0.40	0.003	NA	
	Training experience							0.329
	≤5 years	4	−0.49	−1.4	0.43	0.296	76.8	
	>5 years	5	−1.07	−1.79	−0.36	0.003	61.1	
FSKT-10s		7	0.82	0.15	1.49	0.016	76.8	
	Type of intervention							0.241
	Conditioning activity	2	2.13	0.52	3.74	0.010	82.8	
	Strength and conditioning	4	0.5	0.04	0.96	0.033	4.8	
	Technical training	1	0.25	−0.32	0.82	0.389	0	
	Sex							0.977
	Male	1	1.35	0.58	2.12	0.001	NA	
	Female and male	3	1.28	0.01	2.55	0.049	89.6	
	Unknown	3	0.12	−0.53	0.77	0.718	0	
	Study design							0.526
	RCT	2	0.95	−0.12	2.01	0.082	50.7	
	Crossover	5	0.54	−0.05	1.13	0.071	81.1	
FSKT-mult		9	0.95	0.55	1.35	<0.001	55	
	Intervention duration							0.112
	≤1 week	7	1.07	0.65	1.49	<0.001	53.9	
	>1 week	2	0.34	−0.59	1.28	0.470	32.6	
	Type of intervention							0.072
	Muscle relaxation and recovery class	3	0.67	0.20	1.14	0.005	0	
	Sports training	6	1.11	0.54	1.67	<0.001	65.5	
	Sex							0.420
	Male	4	0.77	0.37	1.16	<0.001	0	
	Female and male	5	1.13	0.43	1.83	0.002	72.2	
	Study design							0.088
	RCT	4	0.86	0.30	1.42	0.002	49.8	
	Crossover	5	1.05	0.40	1.69	0.001	66	
	Body weight							0.094
	≤65 kg	4	1.18	0.34	2.01	0.006	78.9	
	>65 kg	4	0.7	0.26	1.14	0.002	0	
	Unknown	1	0.99	0.26	1.73	0.008	NA	
	Training experience							0.135
	≤5 years	1	0.89	−0.31	2.09	0.145	NA	
	>5 years	8	0.96	0.52	1.39	<0.001	60.6	
VO_2_max		7	1.54	0.58	2.49	0.002	84	
	Type of intervention							0.836
	HIIT	4	2.26	0.30	4.22	0.024	91.6	
	Inspiratory muscle training	2	0.98	0.30	1.66	0.005	0	
	Others	1	0.75	−0.07	1.58	0.073	NA	
	Sex							0.032
	Male	6	0.89	0.52	1.27	<0.001	0	
	Unknown	1	6.83	4.91	8.76	<0.001	NA	
	Body weight							0.744
	≤65 kg	1	0.75	−0.07	1.58	0.073	NA	
	>65 kg	6	1.73	0.56	2.89	0.004	86.4	
	Training experience							0.876
	≤5 years	5	1.80	0.52	3.08	0.006	88.7	
	>5 years	2	1.00	−0.18	2.17	0.096	50.8	

This estimate is based on *k* = 2–3 studies and should be interpreted cautiously due to limited statistical power (see forest plots in Appendix 4).

*Intervention duration*: When the duration was ≤1 week or >1 week, the effects were not statistically significant (SMD = −0.72; 95% CI: −1.53 to 0.09; *I*^2^ = 73.4%; *p* = 0.082) (SMD = −0.90; 95% CI: −1.84 to 0.04; *I*^2^ = 77.8%; *p* = 0.045), with high heterogeneity.

*Type of intervention*: Conditioning activity significantly improved TSAT (SMD = −1.14; 95% CI: −1.69 to −0.59; *I*^2^ = 0%; *p* < 0.001) with low heterogeneity. In contrast, HIIT (SMD = −1.11; 95% CI: −2.65 to 0.43; *I*^2^ = 79.8%; *p* = 0.158), repeated training (SMD = −1.31; 95% CI: −3.34 to 0.71; I^2^ = 84.1%; *p* = 0.204), and other types (SMD = 0.11; 95% CI: −0.38 to 0.60; *I*^2^ = 0%; *p* = 0.670) showed no significant effects, with high heterogeneity in the former two.

*Sex*: Mixed-sex groups showed significant improvement in TSAT (SMD = −1.23; 95% CI: −2.55 to −0.21; *I*^2^ = 82.2%; *p* = 0.018), whereas male-only groups did not (SMD = −0.40; 95% CI: −1.07 to 0.27; *I*^2^ = 53.3%; *p* = 0.241), with high heterogeneity in both.

*Study design*: Crossover studies reported significant effects (SMD = −1.14; 95% CI: −1.69 to −0.58; *I*^2^ = 0%; *p* < 0.001) with low heterogeneity, while RCTs did not reach significance (SMD = −0.72; 95% CI: −1.49 to 0.04; *I*^2^ = 76.3%; *p* = 0.063), with high heterogeneity.

*Body weight*: Significant effects were observed in studies with body weight ≤65 kg (SMD = −1.20; 95% CI: −2.28 to −0.13; *I*^2^ = 81.9%; *p* = 0.028) and unspecified weight (SMD = −1.15; 95% CI: −1.91 to −0.40; *I*^2^ = NA; *p* = 0.003), while no effect was seen in >65 kg groups (SMD = −0.11; 95% CI: −0.59 to 0.38; *I*^2^ = 0%; *p* = 0.666).

*Training experience*: Significant improvement in TSAT was found in >5-year experience groups (SMD = −1.07; 95% CI: −1.79 to −0.36; I^2^ = 61.1%; *p* = 0.003), while ≤5-year groups did not show significant results (SMD = −0.49; 95% CI: −1.40 to 0.43; *I*^2^ = 76.8%; *p* = 0.296).

##### 3.5.1.2 FSKT-10s

Subgroup analysis results for the effect of exercise training on FSKT-10s are shown in Table 5.

*Type of intervention*: Conditioning activity and strength-and-conditioning significantly improved performance (SMD = 2.13; 95% CI: 0.52 to 3.74; *I*^2^ = 82.8%; *p* = 0.010) (SMD = 0.50; 95% CI: 0.04 to 0.96; *I*^2^ = 4.8%; *p* = 0.033). No significant effects were found for other types (SMD = 0.25; 95% CI: −0.32 to 0.82; *I*^2^ = 0%; *p* = 0.389).

*Sex*: Significant effects were observed in male-only groups (SMD = 1.35; 95% CI: 0.58 to 2.12; *I*^2^ = NA; *p* = 0.001) and mixed-sex groups (SMD = 1.28; 95% CI: 0.01 to 2.55; *I*^2^ = 89.6%; *p* = 0.049), with higher heterogeneity in the latter. No effect was found in groups with unspecified sex (SMD = 0.12; 95% CI: −0.53 to 0.77; *I*^2^ = 0%; *p* = 0.718).

*Study design*: Neither RCTs (SMD = 0.95; 95% CI: −0.12 to 2.01; *I*^2^ = 50.7%; *p* = 0.082) nor crossover designs (SMD = 0.54; 95% CI: −0.05 to 1.13; *I*^2^ = 81.1%; *p* = 0.071) reached statistical significance, both showing moderate-to-high heterogeneity.

##### 3.5.1.3 FSKT-mult

To further clarify the effects of exercise training on FSKT-mult, subgroup analyses were conducted based on intervention duration, type, sex, study design, body weight, and training experience (see Table 5).

*Intervention duration*: Significant improvements were observed in studies with duration ≤1 week (SMD = 1.07; 95% CI: 0.65 to 1.49; *I*^2^ = 53.9%; *p* < 0.001), with moderate heterogeneity. No significant effect was found for >1 week (SMD = 0.34; 95% CI: −0.59 to 1.28; *I*^2^ = 32.6%; *p* = 0.470).

*Type of intervention*: Muscle-relaxation/recovery interventions significantly improved performance with no heterogeneity (SMD = 0.67; 95% CI: 0.20 to 1.14; *I*^2^ = 0%; *p* = 0.005). Sports training also showed significant improvement (SMD = 1.11; 95% CI: 0.54 to 1.67; *I*^2^ = 65.5%; *p* < 0.001), though with moderate heterogeneity.

*Sex*: Significant improvements were found in both male-only groups (SMD = 0.77; 95% CI: 0.37 to 1.16; *I*^2^ = 0%; *p* < 0.001) and mixed-sex groups (SMD = 1.13; 95% CI: 0.43 to 1.83; *I*^2^ = 72.2%; *p* = 0.002), with higher heterogeneity in the latter.

*Study design*: Both RCTs (SMD = 0.86; 95% CI: 0.30 to 1.42; *I*^2^ = 49.8%; *p* = 0.002) and crossover designs (SMD = 1.05; 95% CI: 0.40 to 1.69; *I*^2^ = 66%; *p* = 0.001) showed significant effects with moderate heterogeneity.

*Body weight*: Significant effects were observed in ≤65 kg (SMD = 1.18; 95% CI: 0.34 to 2.01; *I*^2^ = 78.9%; *p* = 0.006), >65 kg (SMD = 0.70; 95% CI: 0.26 to 1.14; *I*^2^ = 0%; *p* = 0.002), and unspecified weight groups (SMD = 0.99; 95% CI: 0.26 to 1.73; *I*^2^ = NA; *p* = 0.008), with high heterogeneity in the ≤65 kg group.

*Training experience*: Significant effects were observed in >5-year groups (SMD = 0.96; 95% CI: 0.52 to 1.39; *I*^2^ = 60.6%; *p* < 0.001) with moderate heterogeneity, while no effect was found in ≤5-year groups (SMD = 0.89; 95% CI: −0.31 to 2.09; *I*^2^ = NA; *p* = 0.145).

##### 3.5.1.4 VO_2_max

Subgroup analysis of VO_2_max was conducted based on intervention type, sex, body weight, and training experience (see Table 5).

*Type of intervention*: Both HIIT (SMD = 2.26; 95% CI: 0.30 to 4.22; *I*^2^ = 91.6%; *p* = 0.024) and inspiratory muscle training (SMD = 0.98 95% CI: 0.30 to 1.66; *I*^2^ = 0%; *p* = 0.005) significantly improved VO_2_max, though HIIT showed very high heterogeneity. Other types showed no significant effect (SMD = 0.75; 95% CI: −0.07 to 1.58; *I*^2^ = NA; *p* = 0.073).

*Sex*: Significant improvements were observed in male-only (SMD = 0.89; 95% CI: 0.52 to 1.27; *I*^2^ = 0%; *p* < 0.001) and unspecified-sex groups (SMD = 6.83; 95% CI: 4.91 to 8.76; *I*^2^ = NA; *p* < 0.001).

*Body weight*: Significant improvement was found in >65 kg groups (SMD = 1.73; 95% CI: 0.56 to 2.89; *I*^2^ = 86.4; *p* = 0.004), with high heterogeneity. No significant effect was found in ≤65 kg groups (SMD = 0.75; 95% CI: −0.07 to 1.58; *I*^2^ = NA%; *p* = 0.073).

*Training experience*: In ≤5-year groups, VO₂max was significantly improved (SMD = 1.80; 95% CI: 0.52 to 3.08; *I*^2^ = 88.7%; *p* = 0.006), though heterogeneity was high. No significant effect was observed in >5-year groups (SMD = 0.99; 95% CI: −0.18 to 2.17; *I*^2^ = 50.8%; *p* = 0.096), with moderate heterogeneity.

#### 3.5.2 Subgroup analysis of nutritional supplementations

To further explore the effects of nutritional supplementation on TSAT, FSKT-10s and FSKT-mult performance in taekwondo athletes, subgroup analyses were conducted based on intervention duration, type of nutritional intervention, sex, study design, and training experience. Detailed results are presented in Table 6.

**Table 6:** Subgroup analysis of nutrition supplementation.

Outcome	Covariate	Group	SMD	Lower 95% CI	Upper 95% CI	*p*	Heterogeneity (*I*^2^)	Regression
TSAT		4	1.41	−2.24	0.57	0.001	81.9	
	Intervention duration							0.234
	≤1 week	2	−0.79	−1.15	−0.43	<0.001	84	
	>1 week	2	−2.15	−3.76	−0.54	0.009	0	
	Training experience							0.541
	>5 years	3	−1.68	−2.86	−0.51	0.005	82.3	
	Unknown	1	−0.78	−1.18	−0.39	<0.001	NA	
FSKT-10s		4	1.82	1.08	2.57	<0.001	74.2	
	Intervention duration							0.869
	≤1 week	2	1.46	0.63	2.30	0.001	65.2	
	>1 week	2	2.19	0.84	3.54	0.002	77.4	
	Training experience							0.593
	>5 years	3	2.10	1.32	2.88	<0.001	54.9	
	Unknown	1	1.13	0.71	1.54	<0.001	NA	
FSKT-mult		6	1.67	0.72	2.62	0.001	88.5	
	Intervention duration							0.058
	≤1 week	3	0.86	0.14	1.58	0.019	71	
	>1 week	3	2.57	0.79	4.34	0.005	88.8	
	Type of nutritional intervention							0.067
	Caffeine ingestion	4	2.29	0.89	3.69	0.001	91.4	
	Others	2	0.49	−0.23	1.22	0.180	35	
	Sex							0.029
	Female	2	3.44	2.65	4.23	<0.001	0	
	Female and male	3	1.05	0.53	1.57	<0.001	41.9	
	Unknown	1	0.16	−0.61	0.93	0.690	NA	
	Study design							0.262
	RCT	1	0.90	0.02	1.78	0.046	NA	
	Crossover	5	1.84	0.69	2.99	0.002	90.7	
	Training experience							0.084
	≤5 years	1	0.90	0.02	1.78	0.046	NA	
	>5 years	4	2.15	0.55	3.76	0.009	91.2	
	Unknown	1	0.81	0.41	1.21	<0.001	NA	

This estimate is based on *k* = 2–3 studies and should be interpreted cautiously due to limited statistical power (see forest plots in Appendix 4).

##### 3.5.2.1 TSAT

Subgroup analysis of TSAT was based on intervention duration and training experience, with the following results.

*Intervention duration*: Nutritional supplementation significantly improved TSAT performance in both the ≤1 week subgroup (SMD = −0.79; 95% CI: −1.15 to −0.43; *I*^2^ = 0%; *p* < 0.001) and the >1 week subgroup (SMD = −2.15; 95% CI: −3.76 to −0.51; *I*^2^ = 84%; *p* = 0.009), with low heterogeneity in the 1 week subgroup.

*Training experience*: Significant improvements were observed in the >5 years subgroup (SMD = −1.68; 95% CI: −2.86 to −0.51; *I*^2^ = 82.3%; *p* = 0.005) and the unspecified training experience subgroup (SMD = −0.78; 95% CI: −1.18 to −0.39; *I*^2^ = NA; *p* < 0.001), though the former exhibited high heterogeneity.

##### 3.5.2.2 FSKT-10s

Subgroup analysis of FSKT-10s was based on intervention duration and training experience, with the following results.

*Intervention duration*: Nutritional supplementation significantly improved FSKT-10s performance in both the ≤1 week subgroup (SMD = 1.46; 95% CI: 0.63 to 2.30; *I*^2^ = 65.2%; *p* = 0.001) and the >1 week subgroup (SMD = 2.19; 95% CI: 0.84 to 3.54; *I*^2^ = 77.4%; *p* = 0.002), although both exhibited moderate-to-high heterogeneity.

*Training experience*: Significant improvements were observed in the >5 years subgroup (SMD = 2.10; 95% CI: 1.32 to 2.83; *I*^2^ = 54.9%; *p* < 0.001) and the unspecified training experience subgroup (SMD = 1.13; 95% CI: 0.71 to 1.54; *I*^2^ = NA; *p* < 0.001).

##### 3.5.2.3 FSKT-mult

Subgroup analysis of FSKT-mult was conducted across five covariates: intervention duration, type of nutritional intervention, sex, study design, and training experience.

*Intervention duration*: Nutritional supplementation significantly enhanced FSKT-mult performance in both the ≤1 week subgroup (SMD = 0.86; 95% CI: 0.14 to 1.58; *I*^2^ = 71%; *p* = 0.019) and the >1 week subgroup (SMD = 2.57; 95% CI: 0.79 to 4.34; *I*^2^ = 88.8%; *p* = 0.005), both showing high heterogeneity.

*Type of nutritional intervention*: Caffeine ingestion significantly improved FSKT-mult performance (SMD = 2.29; 95% CI: 0.89 to 3.69; *I*^2^ = 91.4%; *p* = 0.001), though heterogeneity was high. No significant effect was observed for other supplement types (SMD = 0.49; 95% CI: −0.23 to 1.22; *I*^2^ = 35%; *p* = 0.180).

*Sex*: Nutritional supplementation significantly improved FSKT-mult in both female groups (SMD = 3.44; 95% CI: 2.65 to 4.23; *I*^2^ = 0%; *p* < 0.001) and mixed-sex groups (SMD = 1.05; 95% CI: 0.53 to 1.57; *I*^2^ = 41.9%; *p* < 0.001), with higher heterogeneity in the latter. No significant effect was found in unspecified-sex groups (SMD = 0.16; 95% CI: −0.61 to 0.93; *I*^2^ = NA; *p* = 0.690).

*Study design*: Nutritional supplementation was effective in both RCTs (SMD = 0.90; 95% CI: 0.02 to 1.78; *I*^2^ = NA; *p* = 0.046) and crossover studies (SMD = 1.84; 95% CI: 0.69 to 2.99; *I*^2^ = 90.7%; *p* = 0.002), though the latter exhibited high heterogeneity.

*Training experience*: FSKT-mult performance was significantly improved in the ≤5 years subgroup (SMD = 0.90; 95% CI: 0.02 to 1.78; *I*^2^ = NA; *p* = 0.046), the >5 years subgroup (SMD = 2.15; 95% CI: 0.55 to 3.76; *I*^2^ = 91.2%; *p* = 0.009), and the unspecified experience subgroup (SMD = 0.81; 95% CI: 0.41 to 1.21; *I*^2^ = NA; *p* < 0.001).

For a detailed summary of effect sizes and subgroup comparisons, please refer to Table 6.

### 3.6 Sensitivity analysis

To assess the robustness of the meta-analytic findings, sensitivity analyses were performed for all performance-related outcomes in taekwondo athletes. The analyses involved sequentially removing each individual study to examine whether the pooled effect size remained stable—that is, whether the revised estimate after exclusion stayed within the 95% CI of the overall pooled effect.

The results indicated that the exclusion of any single study did not substantially alter the direction or magnitude of the overall effects, suggesting that the findings of this meta-analysis are robust and not unduly influenced by any single trial. Sensitivity analysis results for exercise training are presented in Figures 16–21, and those for nutritional supplementation are shown in Figures 22–27.

**Figure 16:**
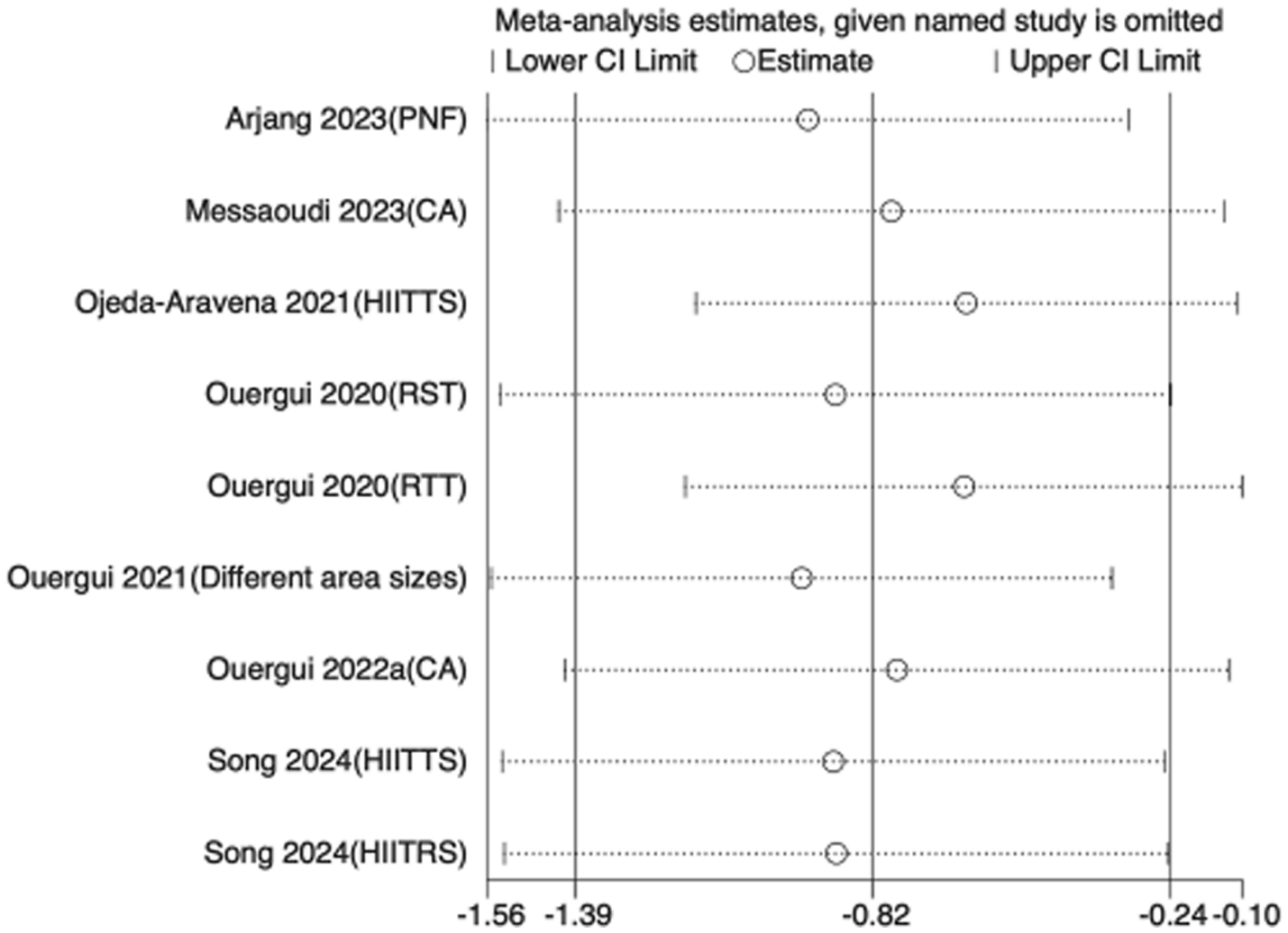
Sensitivity analysis chart of TSAT for exercise training.

**Figure 17:**
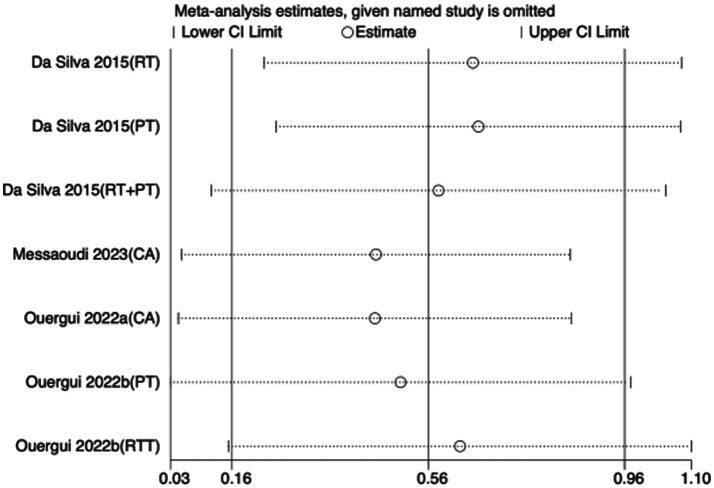
Sensitivity analysis chart of FSKT-10s for exercise training.

**Figure 18:**
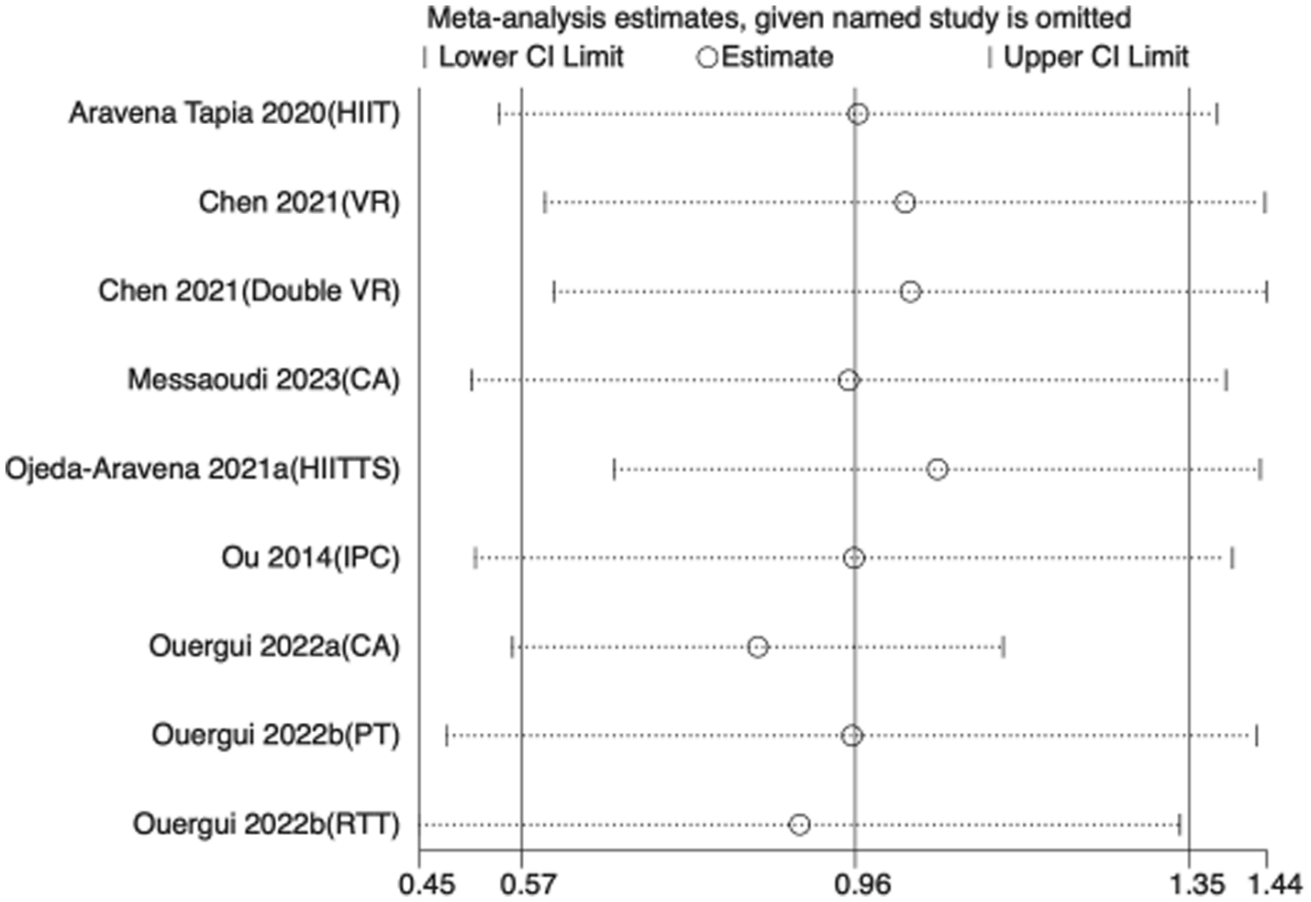
Sensitivity analysis chart of FSKT-mult for exercise training.

**Figure 19:**
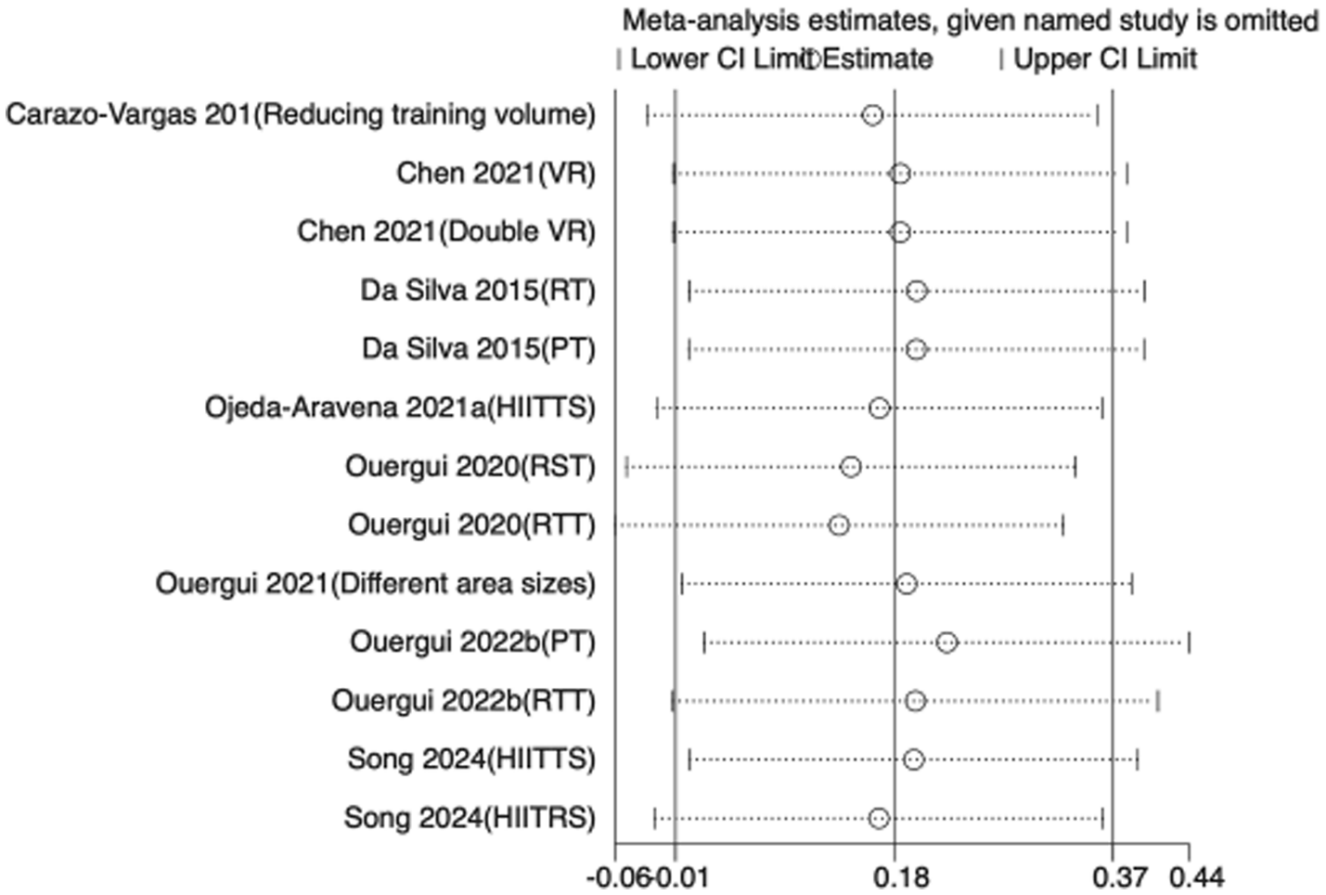
Sensitivity analysis chart of CMJ for exercise training.

**Figure 20:**
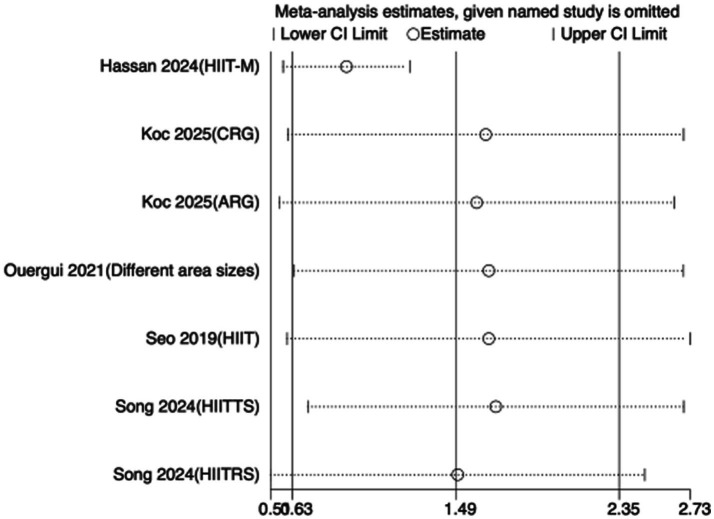
Sensitivity analysis chart of VO_2_max for exercise training.

**Figure 21:**
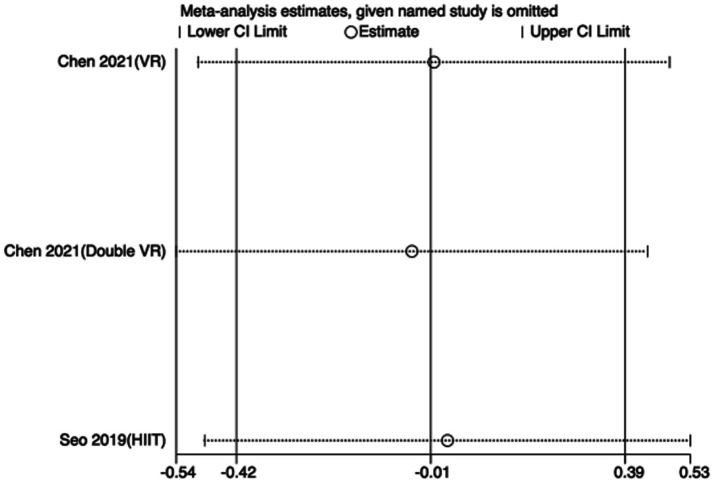
Sensitivity analysis chart of HR_max for exercise training.

**Figure 22:**
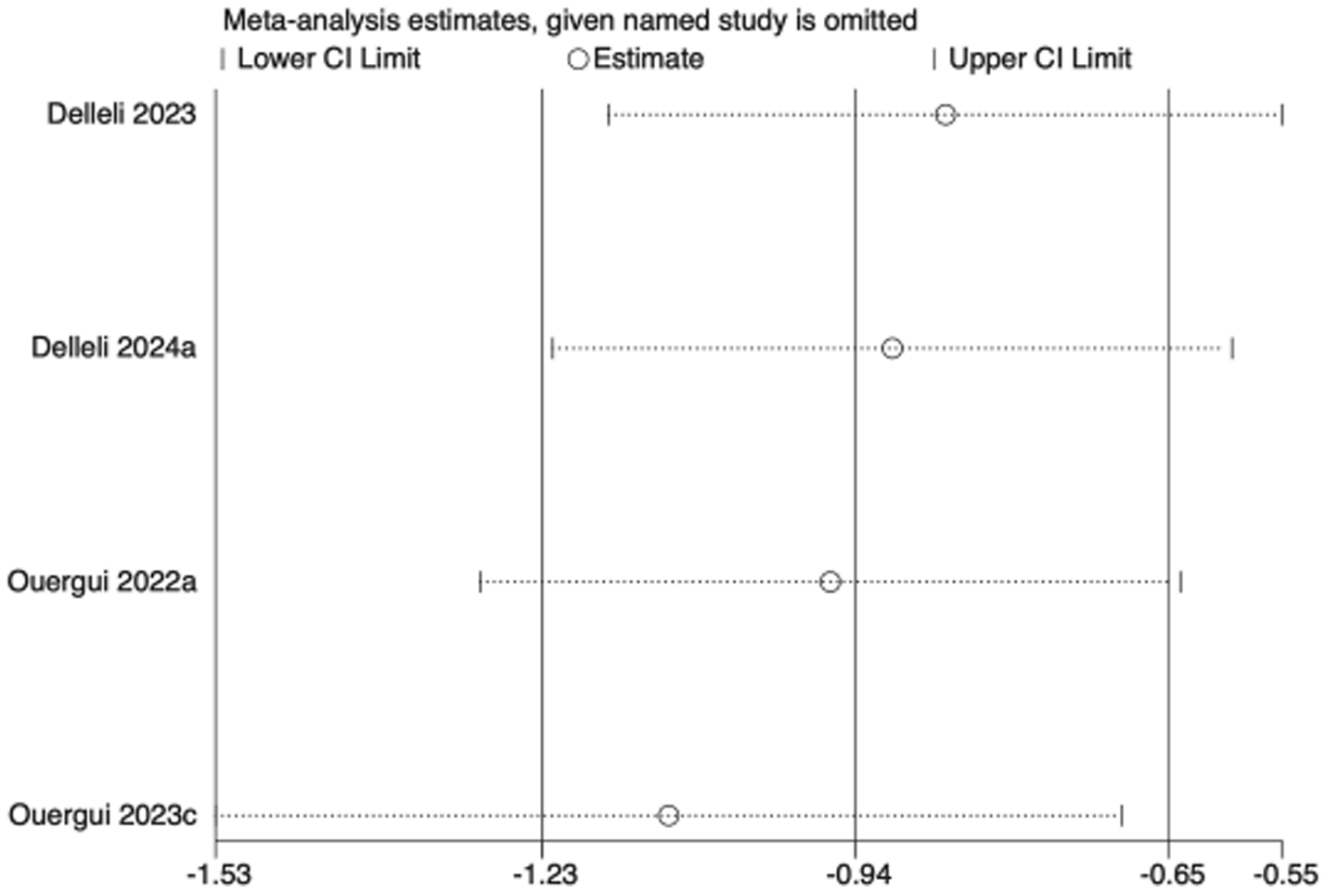
Sensitivity analysis chart of TSAT for nutritional supplementation.

**Figure 23:**
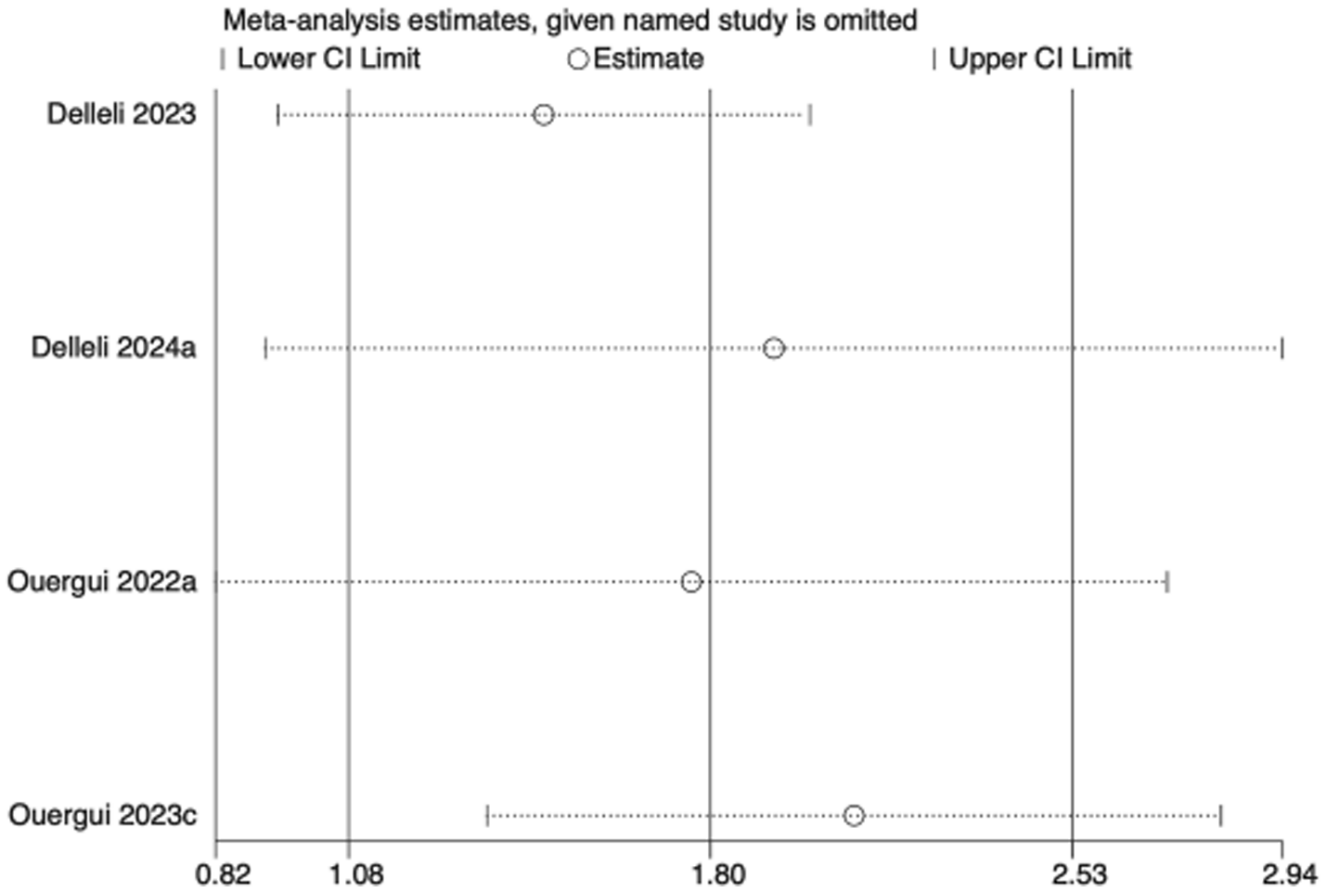
Sensitivity analysis chart of FSKT-10s for nutritional supplementation.

**Figure 24:**
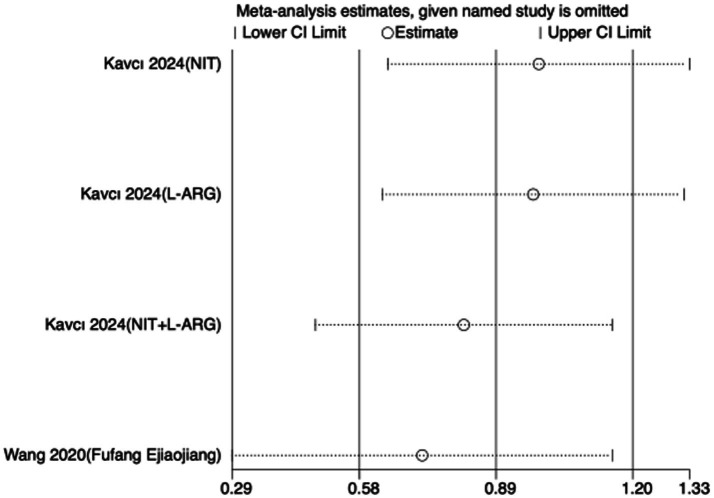
Sensitivity analysis chart of FSKT-mult for nutritional supplementation.

**Figure 25:**
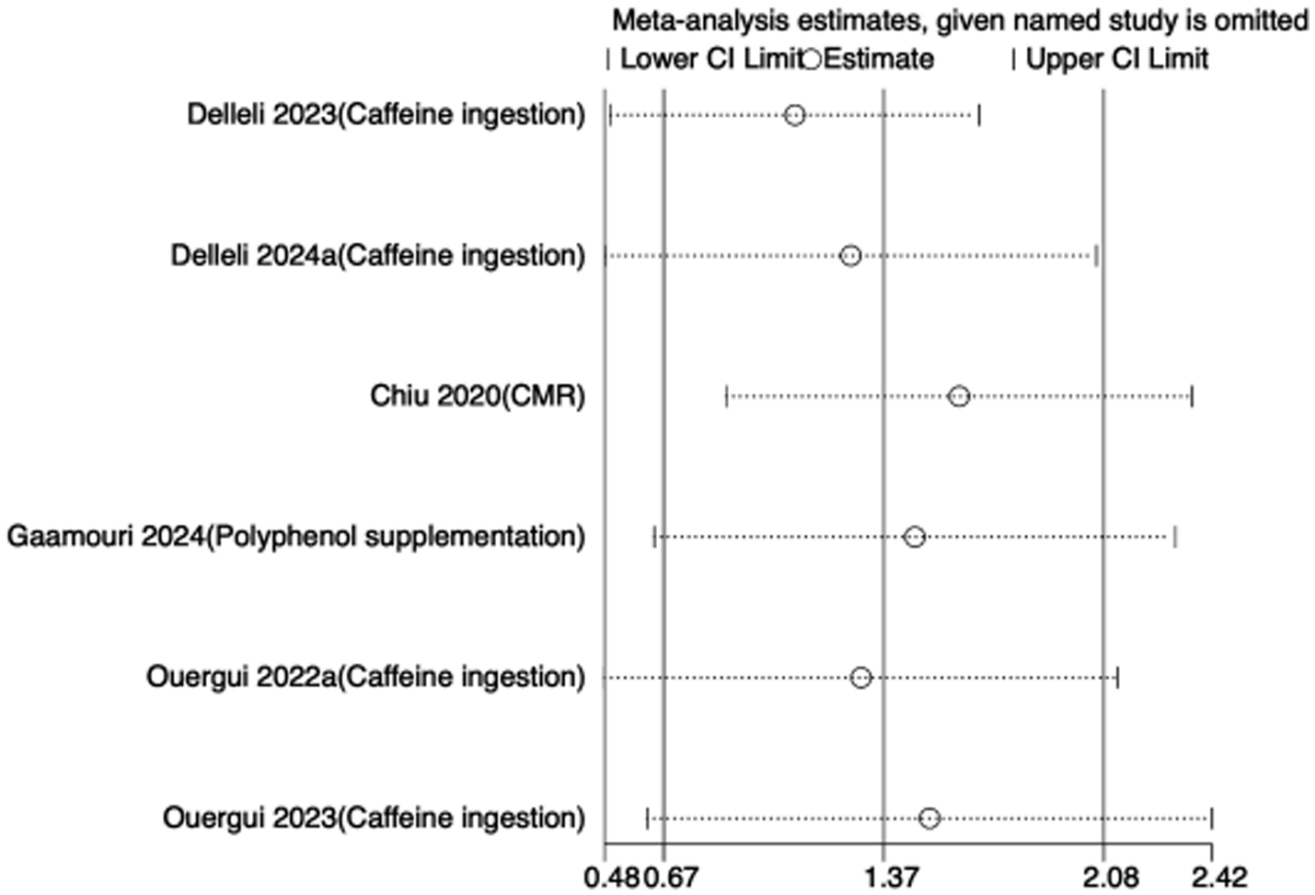
Sensitivity analysis chart of VO_2_max for nutritional supplementation.

**Figure 26:**
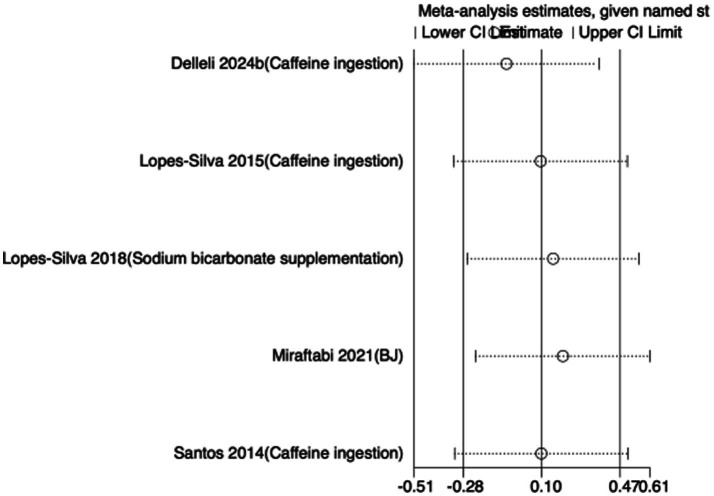
Sensitivity analysis chart of HR_mean for nutritional supplementation.

**Figure 27:**
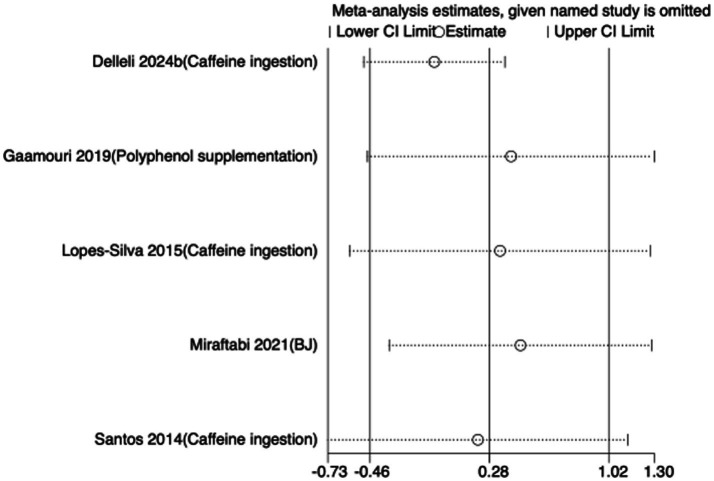
Sensitivity analysis chart of HR_peak for nutritional supplementation.

### 3.7 Publication bias assessment

To further assess the potential presence of publication bias, Egger’s regression test and Begg’s rank correlation test were employed for quantitative evaluation. The Egger’s test results for each outcome are summarized in Table 7.

**Table 7:** Egger’s test results for meta-analysis.

Category	Outcomes	Std_Eff.	Coeff.	Std. Err.	*t*	*p*	95% CI
Exercise							
	TSAT	Slope	1.596401	0.9573729	1.67	0.139	−0.674257 to 3.860229
		Bias	−4.891888	2.057197	−2.38	0.049	−9.756385 to −0.0273907
	FSKT-10s	Slope	0.3738861	1.187167	0.31	0.766	−2.677823 to 3.425596
		Bias	0.9896813	2.883798	0.34	0.745	−6.423359 to 8.402721
	FSKT-mult	Slope	1.320595	0.959099	1.38	0.211	−0.9473135 to 3.588504
		Bias	−0.8895947	2.396389	−0.37	0.721	−6.556153 to 4.776964
	CMJ	Slope	−0.2098457	0.3562123	−0.59	0.567	−0.9859657 to 0.5662743
		Bias	0.9875997	0.8051255	1.23	0.243	−0.7666181 to 2.741818
	VO_2_max	Slope	−2.242839	1.18094	−1.90	0.116	−5.278541 to 0.7928636
		Bias	7.024289	2.389847	2.94	0.032	0.8809921–13.16759
	HR_max	Slope	−0.3571827	0.3278264	−1.09	0.473	−4.522613 to 3.808247
		Bias	0.8432455	0.8120523	1.04	0.488	−9.474858 to 11.16135
Nutrition							
	TSAT	Slope	0.17639	0.8027769	0.22	0.846	−3.27768 to 3.63046
		Bias	−4.282749	2.549081	−1.68	0.235	−15.25056 to 6.685063
	FSKT-10s	Slope	0.1975569	0.4127313	0.48	0.679	−1.578282 to 1.973396
		Bias	4.239404	1.248101	3.4	0.077	−1.130743 to 9.609551
	FSKT-mult	Slope	−0.3681833	0.9293067	−0.4	0.712	−2.948352 to 2.211986
		Bias	4.664441	2.588498	1.8	0.146	−2.522382 to 11.85126
	VO_2_max	Slope	1.277918	0.4015215	3.18	0.086	−0.449689 to 3.005526
		Bias	−0.9692916	1.098192	−0.88	0.471	−5.69443 to 3.755846
	HR_mean	Slope	2.693583	1.078159	2.5	0. 088	−0.7376013 to 6.124767
		Bias	−6.112188	2.525733	−2.42	0.094	−14.1502 to 1.925822
	HR_peak	Slope	8.466328	8.832033	0.96	0.408	−19.64114 to 36.5738
		Bias	−18.89544	20.41808	−0.93	0.423	−83.8749 to 46.08401

Overall, all Egger’s test *p*-values were greater than 0.05—except for TSAT (*p* = 0.049) and VO_2_max (*p* = 0.032) in the exercise intervention subgroup—indicating no significant evidence of publication bias for most outcomes.

For the two potentially biased outcomes (TSAT and VO_2_max), the trim-and-fill method was applied for adjustment. The results revealed that no missing studies were imputed, and the adjusted pooled estimates changed from TSAT: SMD = −0.82 (95% CI: −1.43 to −0.21; *p* = 0.009) to SMD = 0.44 (95% CI: 0.24 to 0.81; *p* = 0.009), and from VO_2_max: SMD = 1.54 (95% CI: 0.58 to 2.49; *p* = 0.002) to SMD = 4.65 (95% CI: 1.79 to 12.08; *p* = 0.002).

The consistency of statistical significance and direction before and after adjustment suggests that the potential publication bias for TSAT and VO_2_max is minimal and has negligible impact on the final conclusions.

Additionally, Begg’s funnel plots were generated to visually inspect publication bias. Most studies were symmetrically distributed within the 95% CI range, and no substantial asymmetry was detected in the funnel plots, confirming the absence of major bias. Detailed results are presented in Appendix 5.

## 4 Discussion

This study systematically evaluated the effects of exercise training and nutritional supplementation on physical performance in taekwondo athletes using a meta-analytic approach. Key performance indicators—including TSAT, FSKT, CMJ, VO_2_max, and HR—were analyzed to provide evidence-based insights into sport-specific and physiological adaptations. Despite the widespread use of both exercise and nutritional interventions in combat sports, their combined or comparative impacts in taekwondo have remained underexplored. The present meta-analysis further examined moderating factors such as intervention duration, type, sex, body weight, and training experience, thereby offering implications for individualized and evidence-based conditioning strategies.

### 4.1 Main findings

#### 4.1.1 Effects of exercise training

Exercise training significantly improved TSAT, FSKT-10s, FSKT-mult, and VO_2_max, confirming its pivotal role in enhancing both agility and aerobic capacity. These findings align with prior evidence showing that resistance, plyometric, and HIIT programs markedly enhance agility and sport-specific speed performance (64–68). Agility, reflecting rapid directional changes and neuromuscular control, is a key determinant of competitive success in taekwondo (67, 68). Improvements in FSKT likely stem from enhanced lower-limb strength, neuromuscular coordination, and anaerobic endurance, as both resistance and HIIT training promote muscle power and fatigue tolerance (69, 70).

The effect on CMJ was small and statistically non-significant, consistent with mixed findings in earlier studies (71–73). This may reflect the limited sensitivity of CMJ to short-term or non-specific interventions that fail to replicate taekwondo’s explosive movement patterns. Conversely, the substantial improvement in VO_2_max supports prior findings that HIIT elicits superior aerobic adaptations compared with steady-state endurance training (66, 74–76). The non-significant changes in HRmax are consistent with evidence suggesting that short-term interventions may not induce measurable cardiac remodeling (37, 77–80).

Physiologically, exercise-induced performance enhancement is mediated by mitochondrial biogenesis, increased capillary density, elevated cardiac output, and neural activation through resistance or plyometric loading (81–84). Moreover, exercise-induced myokines such as IL-6 and irisin (FNDC5) enhance metabolic efficiency, regulate substrate utilization, and delay fatigue (85, 86). Collectively, these findings underscore that systematic and periodized exercise training is fundamental to improving both agility and aerobic fitness in taekwondo athletes.

#### 4.1.2 Main findings of nutritional supplementations

Nutritional supplementation—particularly caffeine—also produced significant improvements in TSAT, FSKT-10s, FSKT-mult, and VO_2_max. The ergogenic effects of caffeine, previously shown to enhance agility, endurance, and fatigue resistance (87–90), are largely mediated by increased calcium release, stimulation of Na^+^/K^+^-ATPase activity, and central nervous system arousal (91–93). These effects appear more pronounced in male athletes and occur even at relatively low doses (0.9–2 mg/kg) (88, 89).

The observed increase in VO_2_max following nitrate or Ejiao supplementation supports evidence that enhanced oxygen transport, improved redox balance, and elevated nitric oxide bioavailability can facilitate endurance performance (94, 95). However, no significant effects were observed for HRmean or HRpeak (96, 97), likely due to small sample sizes, variable testing protocols, and short intervention durations. Similarly, vitamin D supplementation did not improve CMJ, echoing recent findings that vitamin D may exert limited ergogenic influence in well-trained populations (46, 98–100).

Other bioactive compounds, such as polyphenols and dietary nitrates, may further augment performance by promoting vasodilation, reducing oxidative stress, and enhancing muscle oxygenation (101–104). Overall, both exercise and supplementation improved taekwondo performance, though exercise training was more effective for agility and VO_2_max, while caffeine supplementation provided superior benefits for repeated kicking performance (FSKT).

### 4.2 Subgroup analysis interpretation

#### 4.2.1 Exercise training subgroups

Subgroup analyses revealed that intervention duration, type, sex, study design, body weight, and training experience moderated the effects of exercise training. Short-term interventions (≤1 week) yielded greater improvements in FSKT-mult, possibly reflecting acute neuromuscular potentiation and fatigue recovery, whereas longer-term programs were required for substantial agility gains (TSAT). Conditioning activities enhanced both TSAT and FSKT-10s, while HIIT and inspiratory muscle training produced the largest VO_2_max gains (105–107).

Male athletes exhibited smaller improvements in agility, likely due to higher baseline neuromuscular power, while lighter athletes (≤65 kg) achieved greater agility benefits and heavier athletes (>65 kg) improved VO₂max, possibly reflecting differences in body composition and oxygen utilization efficiency. Athletes with >5 years of experience demonstrated superior gains in agility and anaerobic performance, whereas less experienced athletes exhibited larger aerobic adaptations (70, 108, 109).

These results highlight the necessity of experience- and physiology-specific training prescriptions to maximize taekwondo performance.

#### 4.2.2 Nutritional supplementation subgroups

Both short- and long-term nutritional interventions enhanced agility and repeated kicking performance, reflecting improvements in energy metabolism, oxygen utilization, and fatigue resistance (90). Caffeine supplementation produced the most consistent effects on FSKT-mult, attributable to enhanced calcium ion kinetics, increased motor unit recruitment, and delayed central fatigue (110, 111).

Future studies should explore synergistic combinations of ergogenic aids (e.g., caffeine with nitrates or amino acids) to determine additive effects across performance domains and metabolic pathways.

### 4.3 Strengths, limitations, and future perspectives

This meta-analysis represents the first comprehensive synthesis of both exercise and nutritional interventions targeting taekwondo athletes. It integrated six key physiological and performance indicators, thereby offering an evidence-based framework for optimizing training and supplementation strategies in combat sports.

However, several limitations should be acknowledged. First, considerable heterogeneity in study design, sample size, and measurement protocols may affect the generalizability of findings. Second, most studies investigated single interventions with short-term follow-up, precluding conclusions about long-term or combined effects. Third, several subgroup analyses were based on small samples (*k* = 2–3), limiting statistical power and precision; these results should therefore be interpreted as exploratory. Fourth, although publication bias was generally low, the restriction to English-language, peer-reviewed studies may still have introduced selection bias.

Future research should focus on large-scale, multicenter randomized controlled trials employing standardized testing and longitudinal tracking. Integrating physiological, biochemical, and psychological indicators will yield a more comprehensive understanding of performance adaptations. Moreover, AI-driven analytics and wearable technology could enable real-time performance monitoring, predictive modeling, and precision-tailored training or nutrition interventions, ultimately advancing the scientific basis for elite taekwondo performance optimization.

## 5 Conclusion

Exercise training significantly improved TSAT, FSKT-10s, FSKT-mult, and VO₂max, while CMJ and HRmax showed no significant effects. Nutritional supplementation, especially caffeine, enhanced TSAT, FSKT-10s, FSKT-mult, and VO₂max but did not affect HRmean or HRpeak.

Despite methodological heterogeneity, these findings offer practical, evidence-based guidance for designing integrated conditioning and supplementation strategies in taekwondo. Tailoring programs to athlete characteristics, experience level, and physiological demands may further enhance performance outcomes.

## Data Availability

The datasets analyzed in this study are available from the corresponding author upon reasonable request.
