# Supplementary material for: The effects of exercise training and nutritional supplementation on taekwondo performance: a systematic review and meta-analysis
Source: Front Nutr. 2025 Dec 11;12:1618612. doi: 10.3389/fnut.2025.1618612 (PMC12739882; doi:10.3389/fnut.2025.1618612)
Supplement: Supplementary file 1 [file Table_1.DOCX]

**Appendix 1:Search Strategy**

(1) PubMed

((taekwondo athletes[Title/Abstract] OR taekwondo athlete[Title/Abstract]OR taekwondo[Title/Abstract]) AND (VO2max[Title/Abstract] OR maximal oxygen uptake[Title/Abstract] OR maximal oxygen consumption[Title/Abstract] OR heart rate[Title/Abstract] OR HR[Title/Abstract] OR countermovement jump[Title/Abstract] OR CMJ[Title/Abstract] OR taekwondo-specific agility test[Title/Abstract] OR TSAT[Title/Abstract] OR frequency speed of kick test[Title/Abstract] OR FSKT[Title/Abstract] OR aerobic[Title/Abstract] OR aerobic[Title/Abstract])) NOT (patient[Title/Abstract] OR patients[Title/Abstract])

(2) Scopus

( ( TITLE-ABS-KEY ( taekwondo AND athletes ) OR TITLE-ABS-KEY ( taekwondo AND athlete ) OR TITLE-ABS-KEY ( taekwondo ) ) ) AND ( ( TITLE-ABS-KEY ( maximal AND oxygen AND uptake ) OR TITLE-ABS-KEY ( vo2max ) OR TITLE-ABS-KEY ( maximal AND oxygen AND consumption ) OR TITLE-ABS-KEY ( heart AND rate ) OR TITLE-ABS-KEY ( hr ) OR TITLE-ABS-KEY ( countermovement AND jump ) OR TITLE-ABS-KEY ( cmj ) OR TITLE-ABS-KEY ( taekwondo-specific AND agility AND test ) OR TITLE-ABS-KEY ( tsat ) OR TITLE-ABS-KEY ( frequency AND speed AND of AND kick AND test ) OR TITLE-ABS-KEY ( fskt ) OR TITLE-ABS-KEY ( aerobic ) OR TITLE-ABS-KEY ( anaerobic ) ) ) AND NOT ( ( TITLE-ABS-KEY ( patient ) OR TITLE-ABS-KEY ( patients ) ) )

(3) Web of science

((TS=(taekwondo athletes OR taekwondo athlete)) AND TS=(VO2max OR maximal oxygen uptake OR maximal oxygen consumption OR heart rate OR HR OR countermovement jump OR CMJ OR taekwondo-specific agility test OR TSAT OR frequency speed of kick test OR FSKT OR aerobic OR aerobic)) NOT TS=(patient OR patients)

**Appendix 2: Overview of Exercise Training Interventions and Number of Studies per Category**

| No. | Intervention | Number of Studies | Description of Interventions |
| --- | --- | --- | --- |
| 1 | Usual training | 16 | Regular taekwondo training as a control condition, general warm-up, including placebo. |
| 2 | Acute Inspiratory Training Group (ARG) | 1 | Inspiratory muscle training (IMT) at 40–50% maximal inspiratory pressure (MIP) for approximately 20 minutes, three times weekly during pre-training warm-up. |
| 3 | Chronic Inspiratory Training Group (CRG) | 1 | IMT at 60–90% MIP with 30 breaths, performed twice daily for 8 weeks. |
| 4 | Conditioning Activity (CA) | 2 | Performing 3 sets of 10 vertical jumps above 40 cm. |
| 5 | Different Area Sizes | 1 | Training sessions conducted in three varied area sizes: 4 × 4 m, 6 × 6 m, and 8 × 8 m. |
| 6 | Double Vibration Foam Rolling (Double VR) | 1 | Six sets of vibration foam rolling specifically targeting the weaker leg. |
| 7 | High-Intensity Interval Training Repeated Sprints (HIITRS) | 1 | Three sets of 10 repetitions of 4-second maximal linear sprints over 5 m, each followed by 28-second walking-based recovery. |
| 8 | High-Intensity Interval Training with Breathing Mask (HIIT-M) | 1 | HIIT performed 3 times weekly for 60–90 minutes, involving short-duration, high-intensity exercises with a breathing mask and 30–60 seconds rest intervals. |
| 9 | High-Intensity Interval Training (HIIT) | 2 | High-intensity exercises at 85–100% HRmax with rest intervals between sets. |
| 10 | High-Intensity Interval Training with Specific Techniques (HIITTS) | 2 | Three rounds of 2-minute sets consisting of 4-second maximal effort kicks followed by 28 seconds of active recovery (1:7 ratio). |
| 11 | Ischemic Preconditioning (IPC) | 1 | Application of pressure at 220 mmHg for preconditioning purposes. |
| 12 | Plyometric Training (PT) | 1 | Three sets of 10 consecutive vertical jumps over a 40 cm obstacle. |
| 13 | Proprioceptive Neuromuscular Facilitation (PNF) | 1 | PNF stretching applied to one leg. |
| 14 | Reducing Training Volume | 1 | Progressive linear reduction of training volume by 3.33% per session, reaching 50% of the original volume at the end of tapering. |
| 15 | Repeated High-Intensity Techniques Training (RTT) | 2 | Three sets of alternating kicks (Bandal-chagui) executed maximally for 5 seconds, with a 10-second rest interval between sets. |
| 16 | Repeated Sprints Training (RST) | 1 | Performing 10 sets of 35 m sprints with 10-second rest intervals. |
| 17 | Resistance Training and Plyometric Training (RT+PT) | 1 | Three half-squats followed immediately by four vertical jumps over a 40 cm barrier. |
| 18 | Resistance Training (RT) | 1 | Three sets of 1 repetition half-squats at 95% 1RM, including deep squat, deadlift, and kettlebell squat jumps. |
| 19 | Vibration Foam Rolling (VR) | 1 | Three sets of foam rolling targeting quadriceps and hamstrings after warm-up. |

**Appendix 3: Overview of Nutritional Supplementation Interventions and Number of Studies per Category**

| No. | Intervention | Number of Studies | Description of Interventions |
| --- | --- | --- | --- |
| 1 | Usual training | 17 | Regular taekwondo training as a control condition, general warm-up, including placebo. |
| 2 | Caffeine ingestion | 7 | Caffeine supplementation (3.5 mg/kg/day). |
| 3 | Carbohydrate Mouth Rinsing (CMR) | 1 | Mouth rinsing with carbohydrate solution (25 mL, 6.4% maltodextrin) for 15 seconds. |
| 4 | Fufang Ejiaojiang | 1 | Supplementation with 1 tablet daily containing donkey-hide gelatin, Codonopsis pilosula, prepared rehmannia root, red ginseng, maybush, and sucrose. |
| 5 | L-Arginine Supplements (L-ARG) | 1 | Single-dose supplementation of 6 g powdered L-arginine. |
| 6 | Nitrate-Rich Beetroot Juice (BJ) | 1 | Beetroot juice supplementation (400–800 mg of nitrate). |
| 7 | Nitrate and L-Arginine Supplements (NIT+L-ARG) | 1 | Combined supplementation with nitrate (837.40 mg/kg from spinach) and a single dose of 6 g L-arginine powder. |
| 8 | Nitrate Supplements (NIT) | 1 | Nitrate supplementation provided by spinach (837.40 mg/kg). |
| 9 | Nucleotide Formulation (Inmunactive) | 1 | Daily supplementation of 480 mg Inmunactive for 30 days. |
| 10 | Polyphenol Supplementation | 2 | Carob supplementation at a dosage of 40 g/day. |
| 11 | Sodium Bicarbonate Supplementation (NaHCO3) | 1 | Supplementation of 300 mg/kg body mass of sodium bicarbonate. |
| 12 | Vitamin D | 1 | Daily supplementation with one capsule containing 5,000 IU vitamin D3. |

**Appendix 4. Forest Plots of Subgroup Analyses**

**Exercise Training:**

**TSAT**:

**Intervention duration**

**
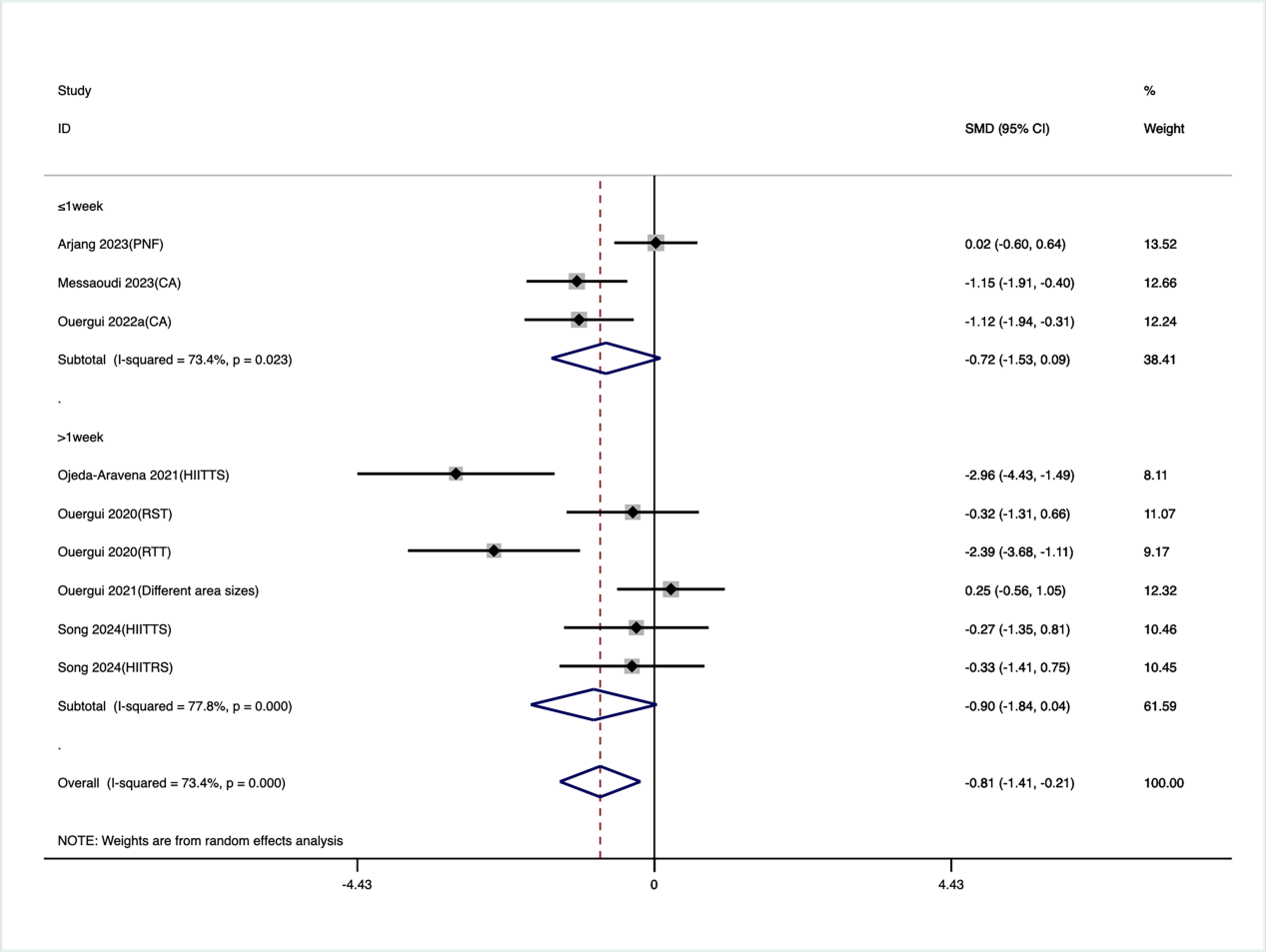
**

**Type of intervention**

**
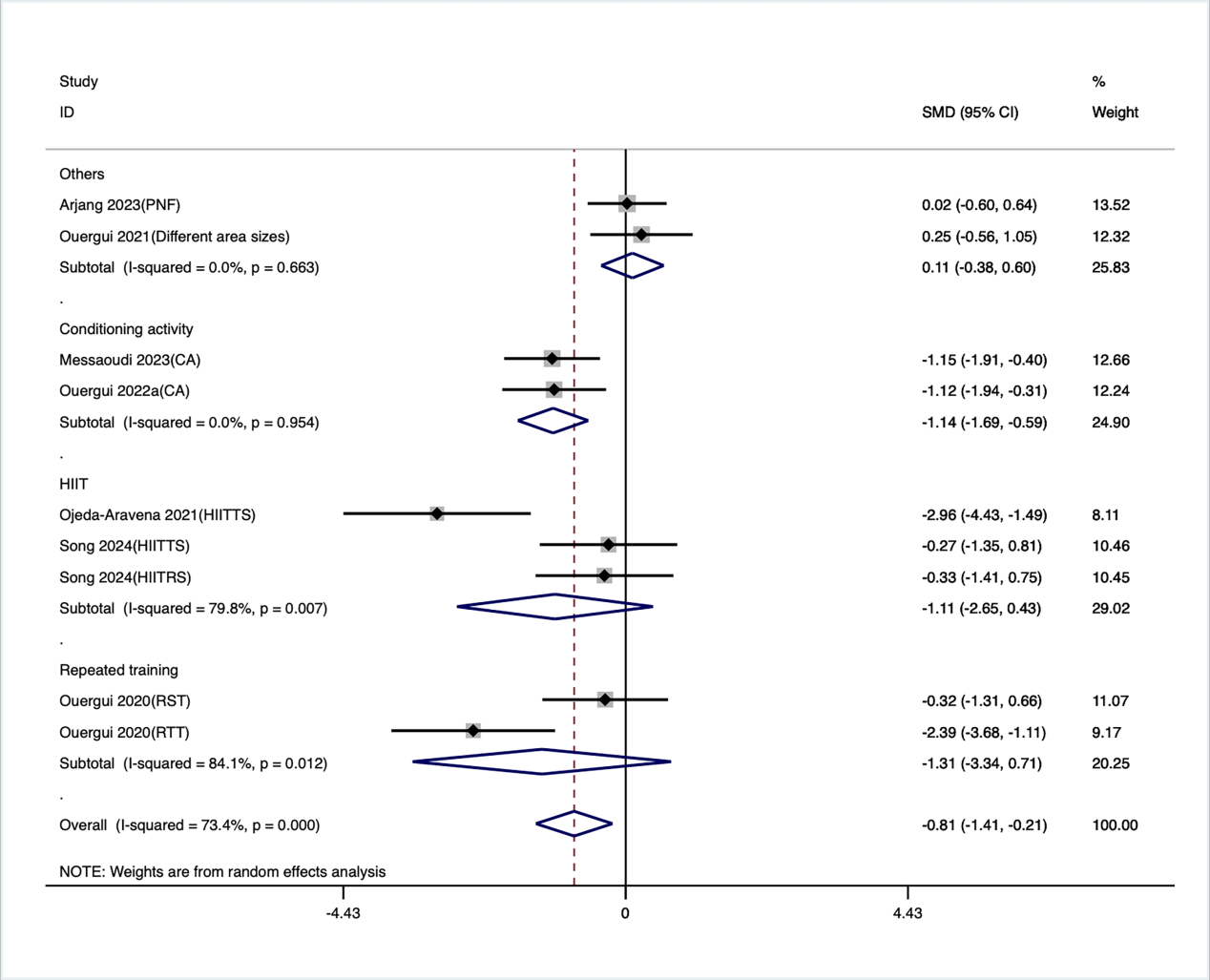
**

**Sex**

**
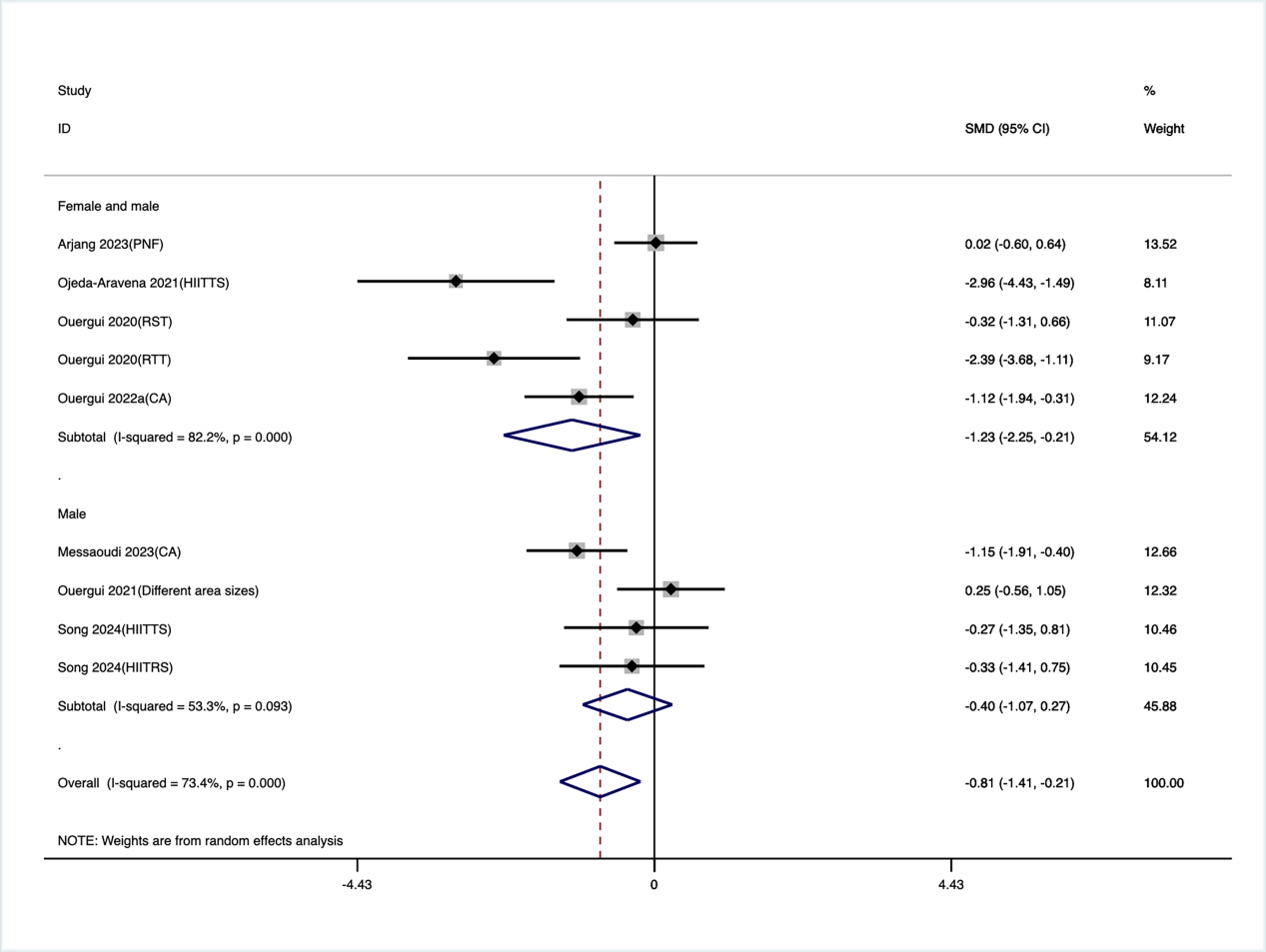
**

**Study design**

**
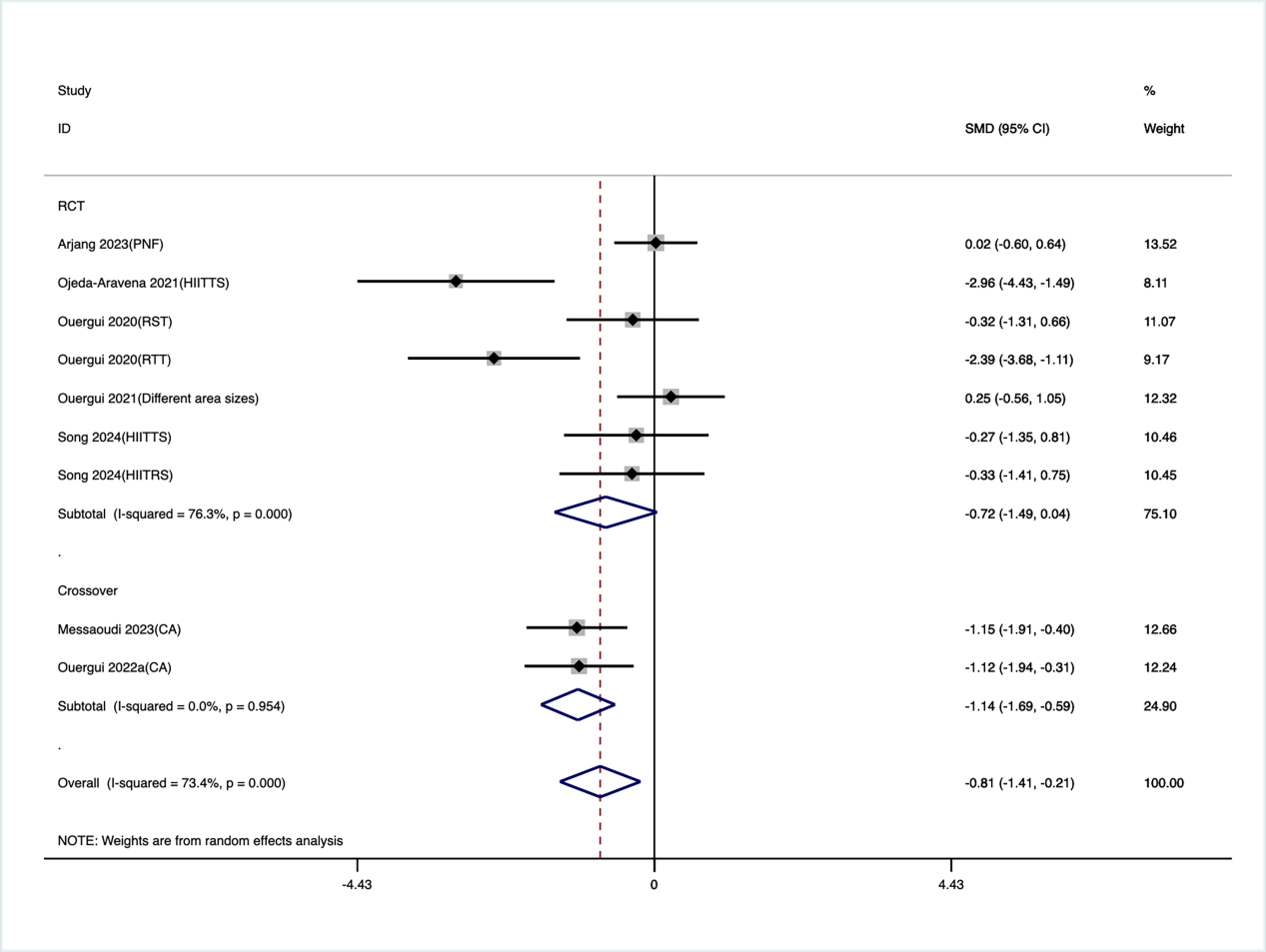
**

**Body weight**

**
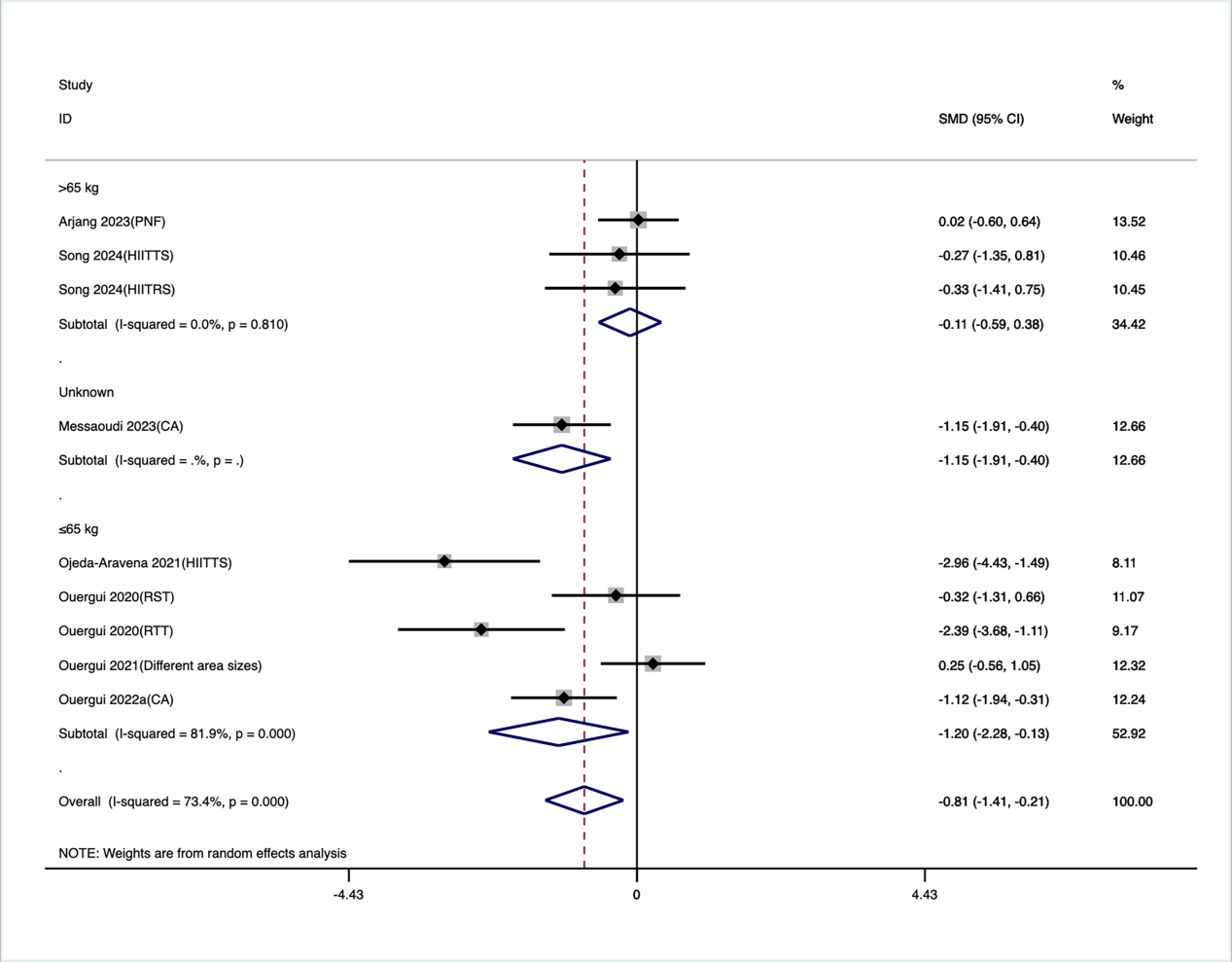
**

**Training experience**

**
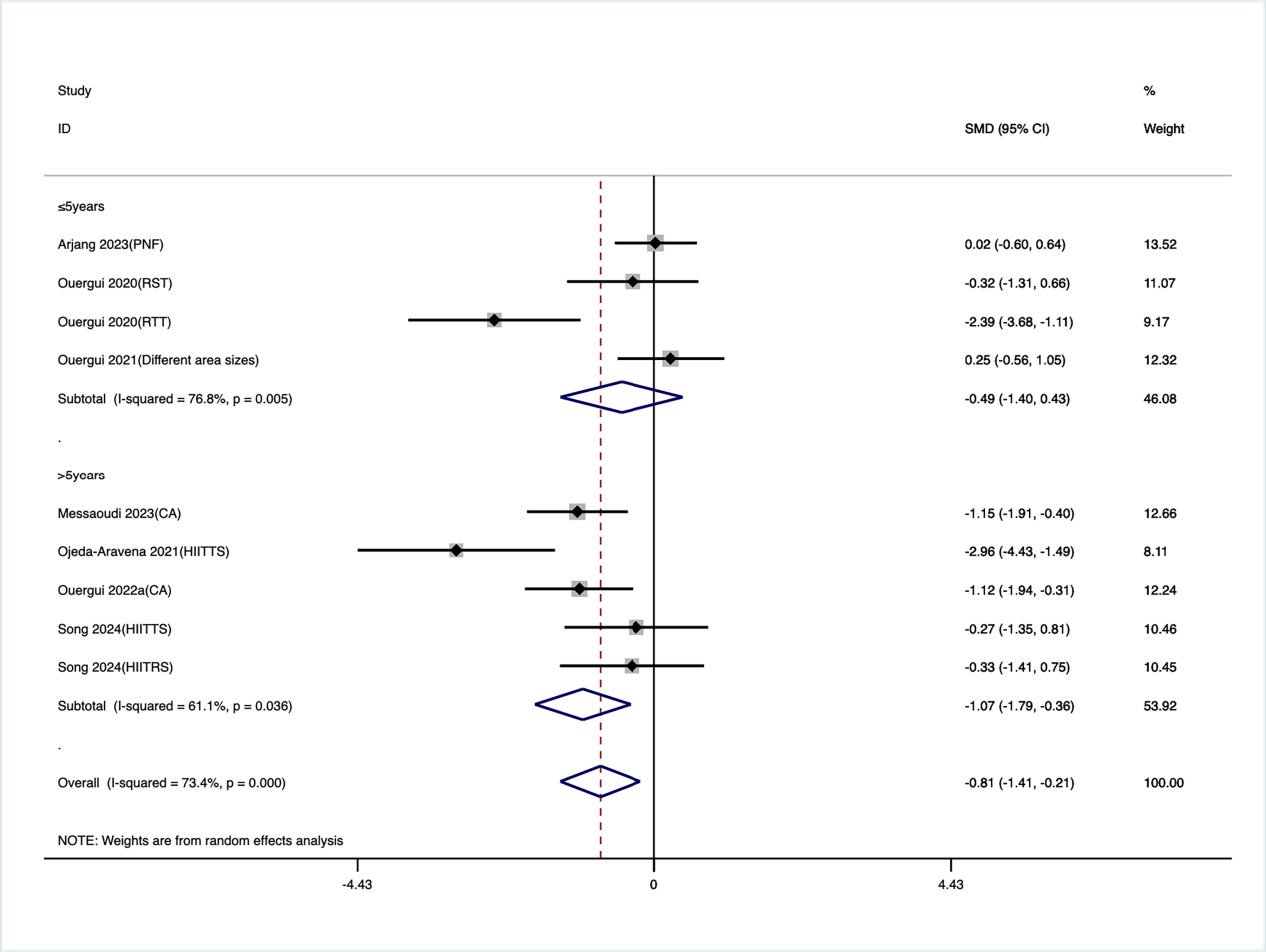
**

**FSKT-10s:**

**Type of intervention**

**
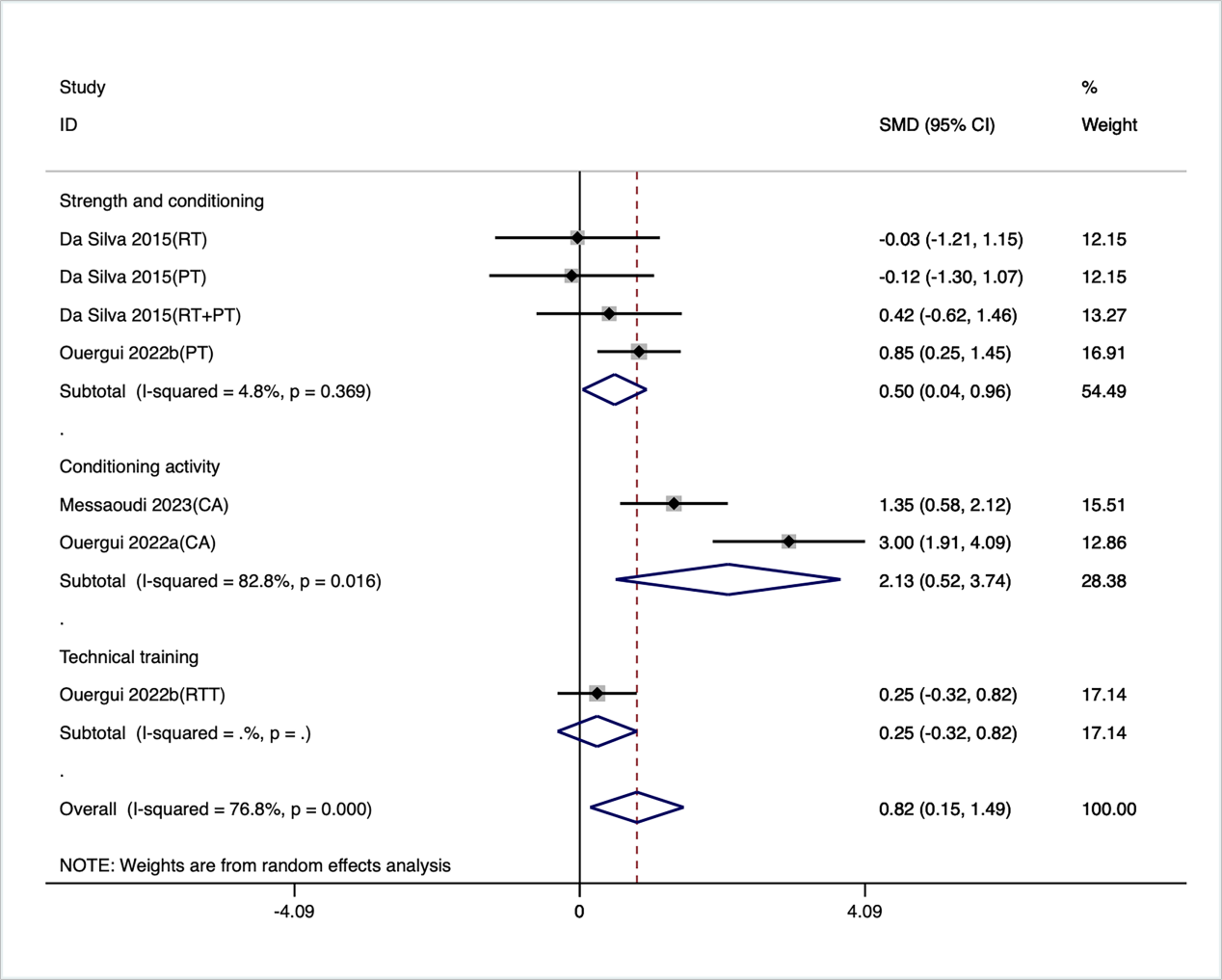
**

**Sex**

**
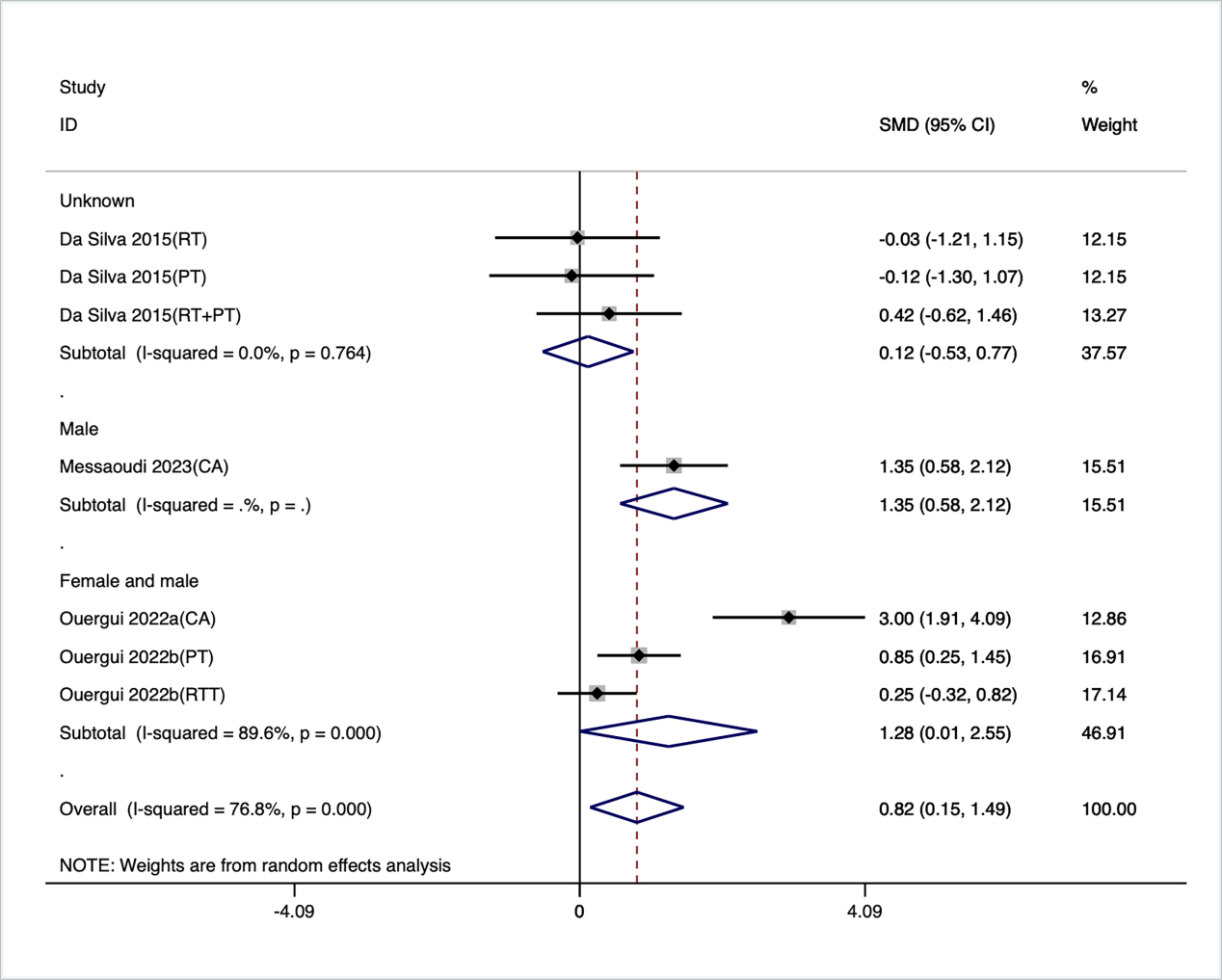
**

**Study design**

**
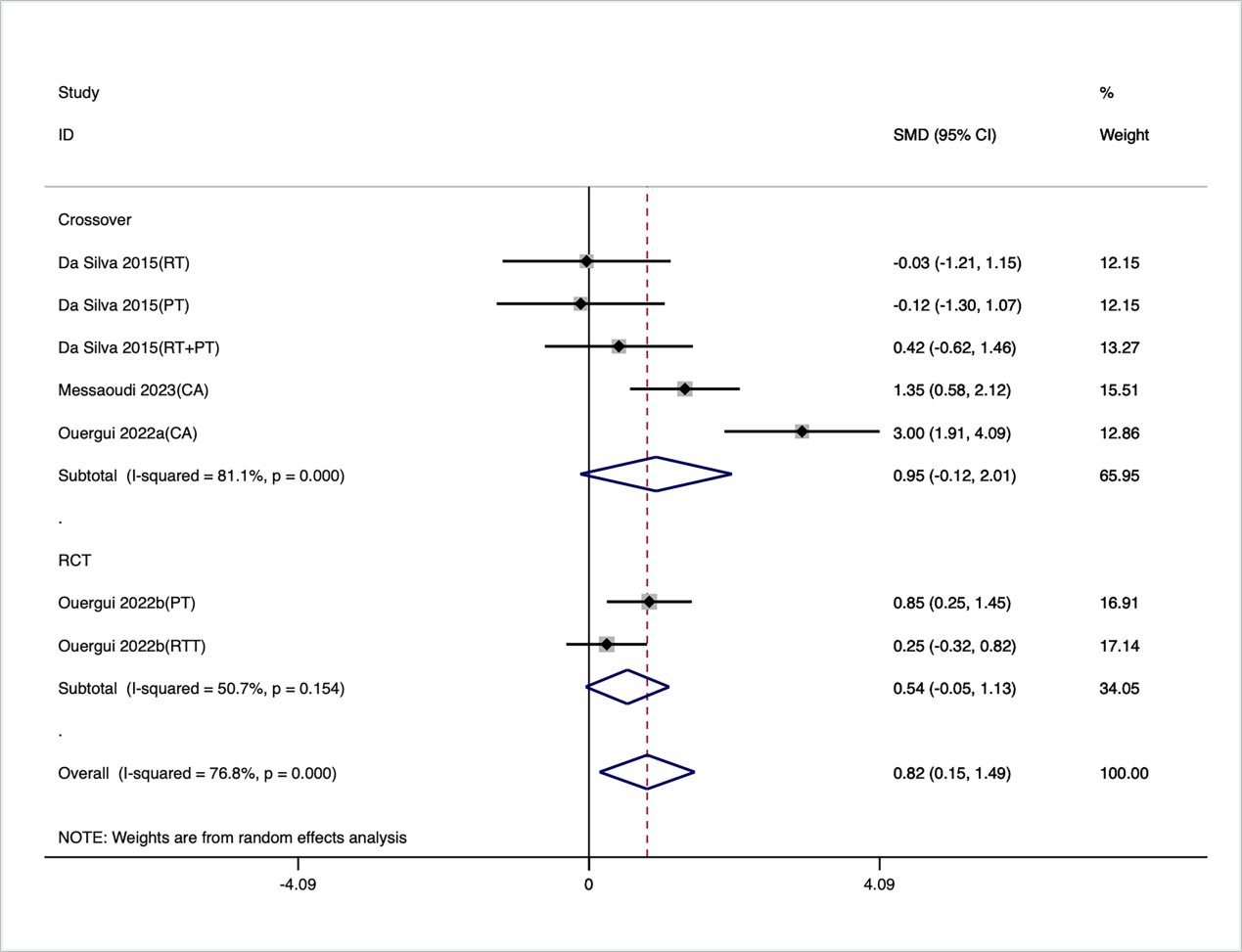
**

**FSKT-mult:**

**Intervention duration**

**
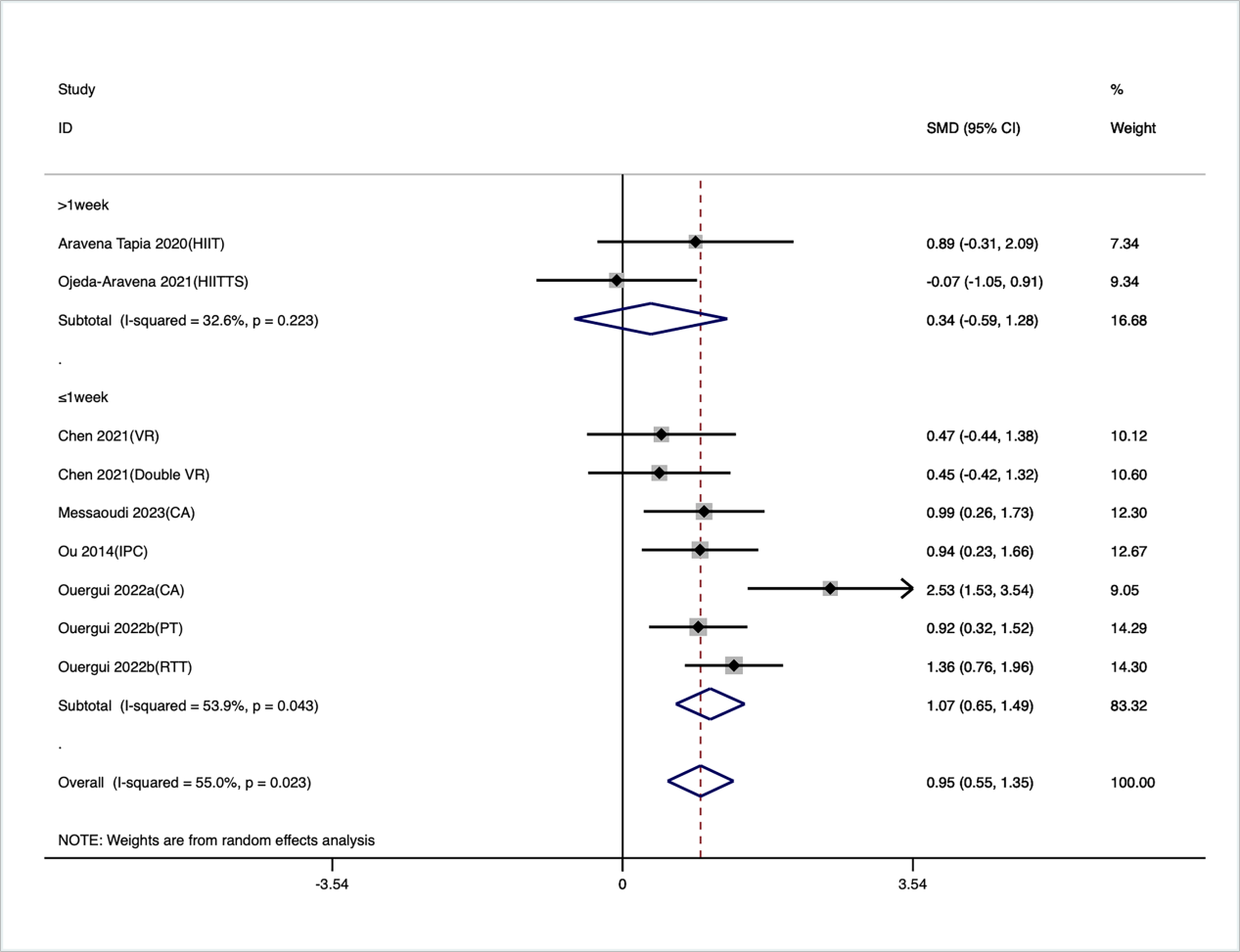
**

**Type of intervention**

**
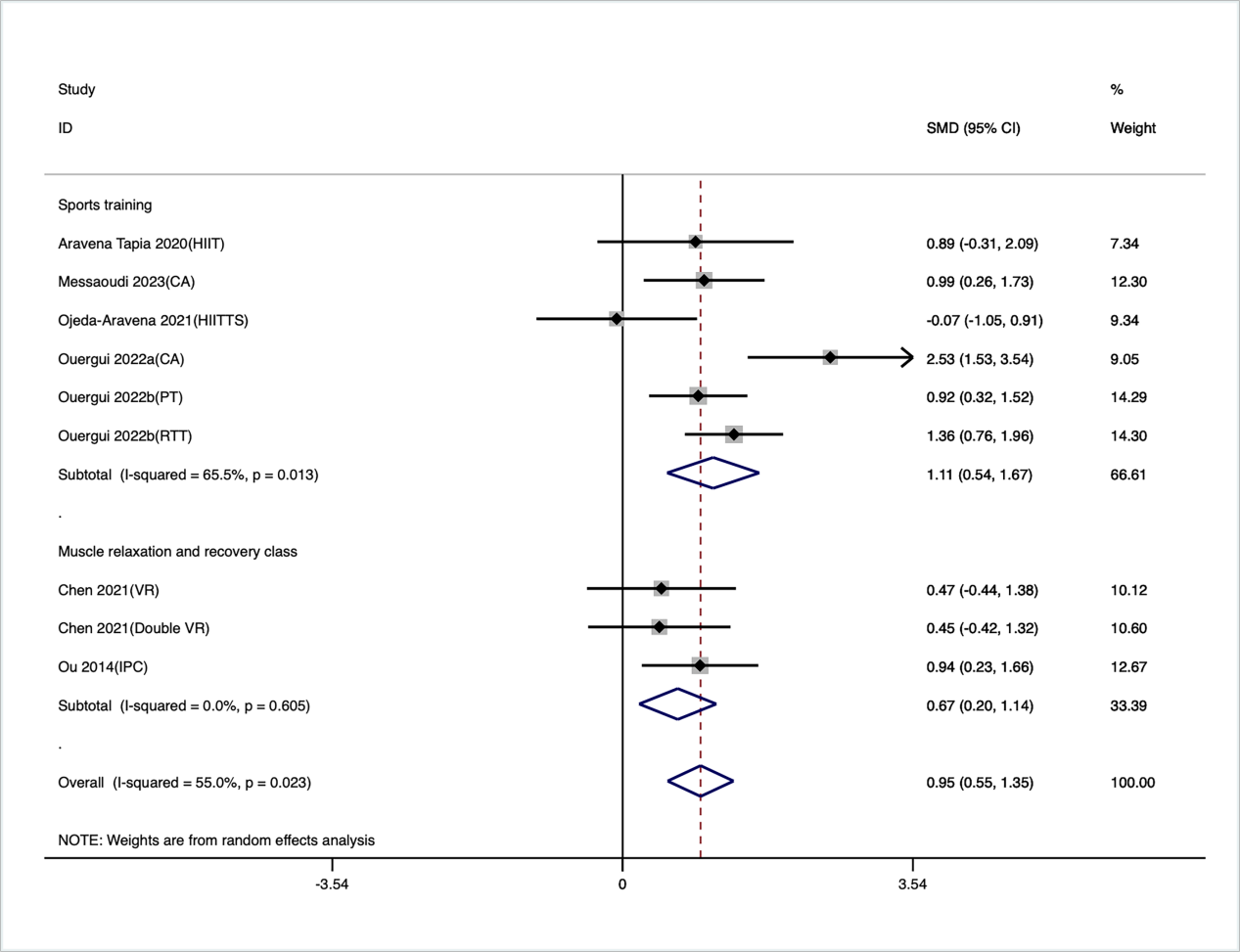
**

**Sex**

**
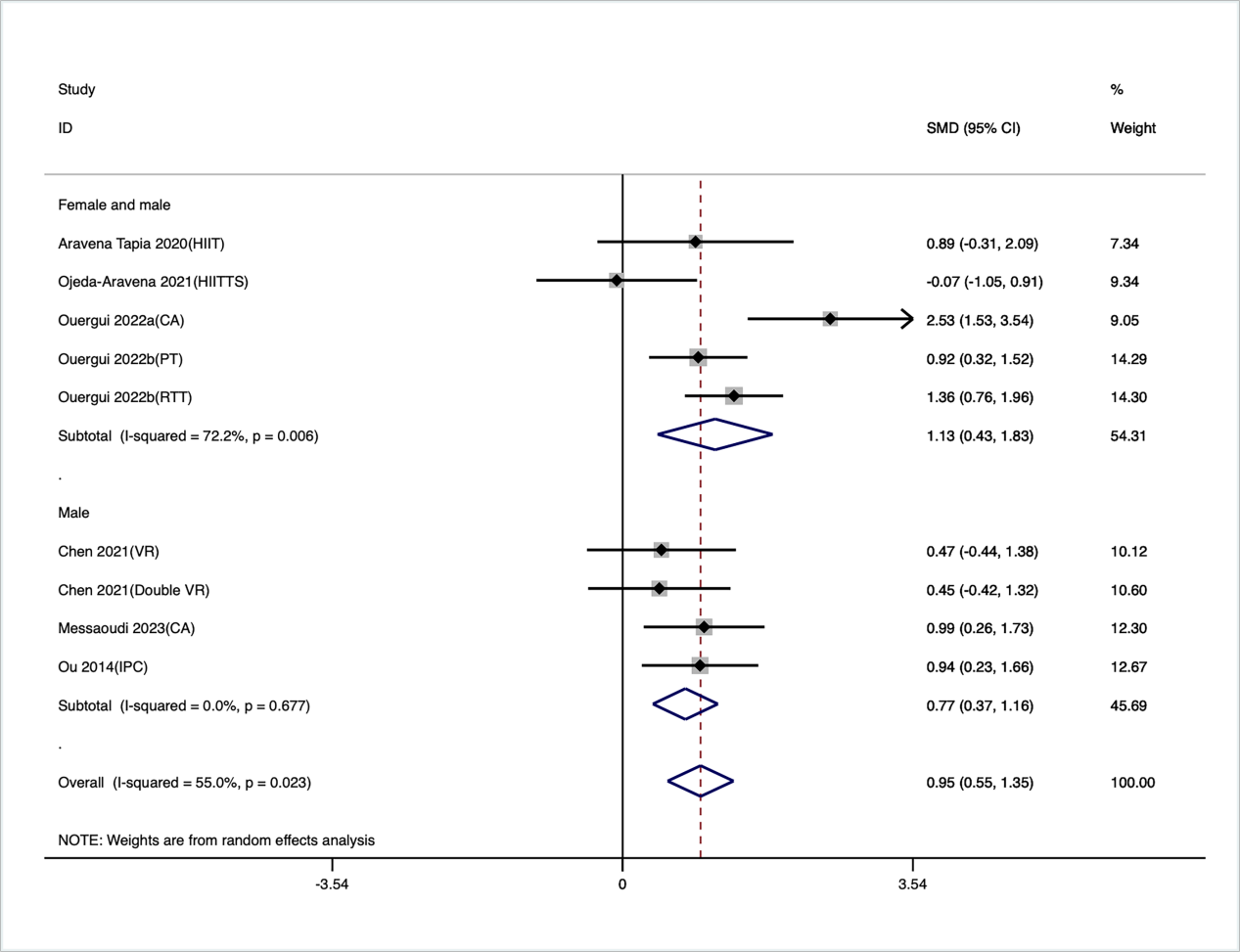
**

**Study design**

**
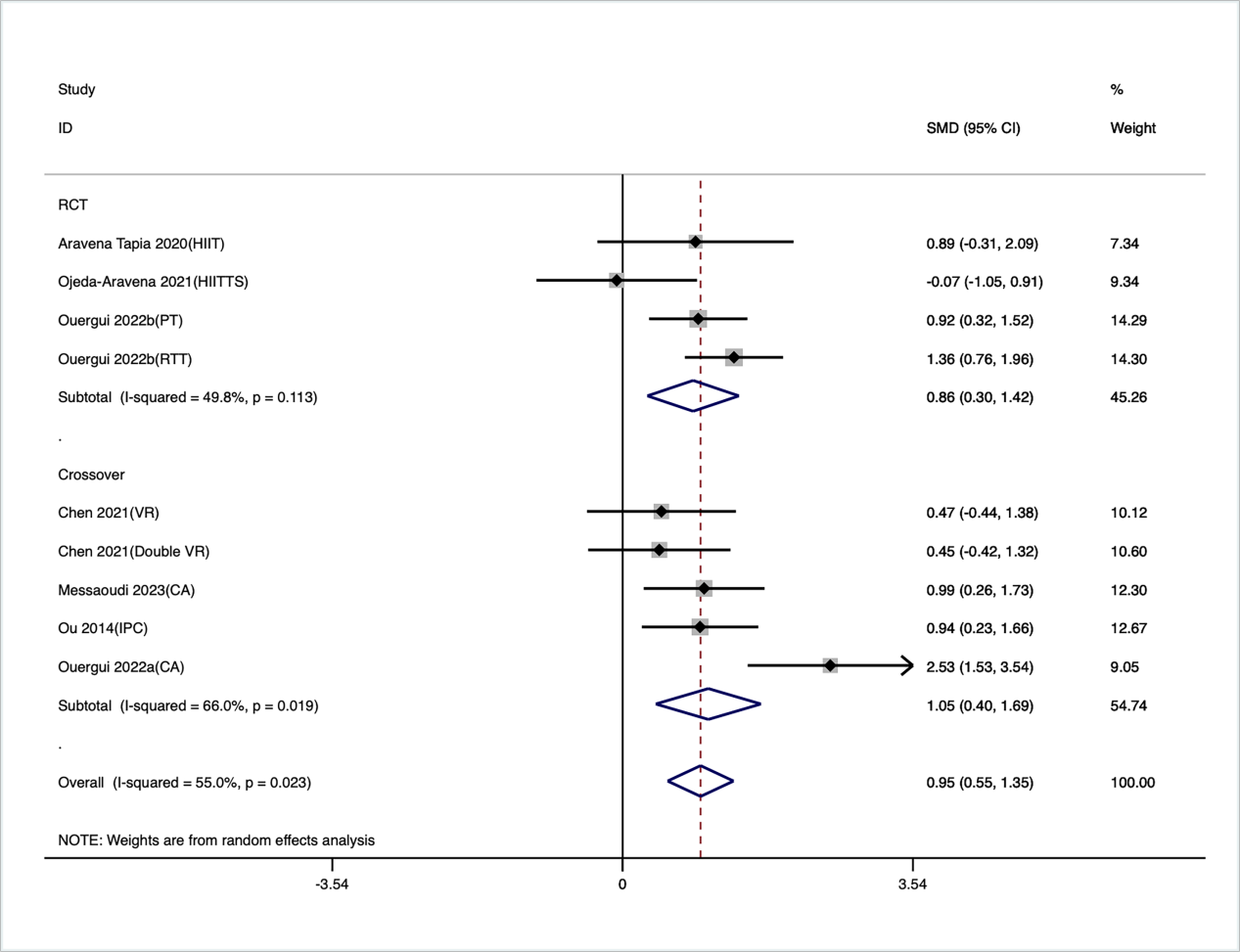
**

**Body weight**

**
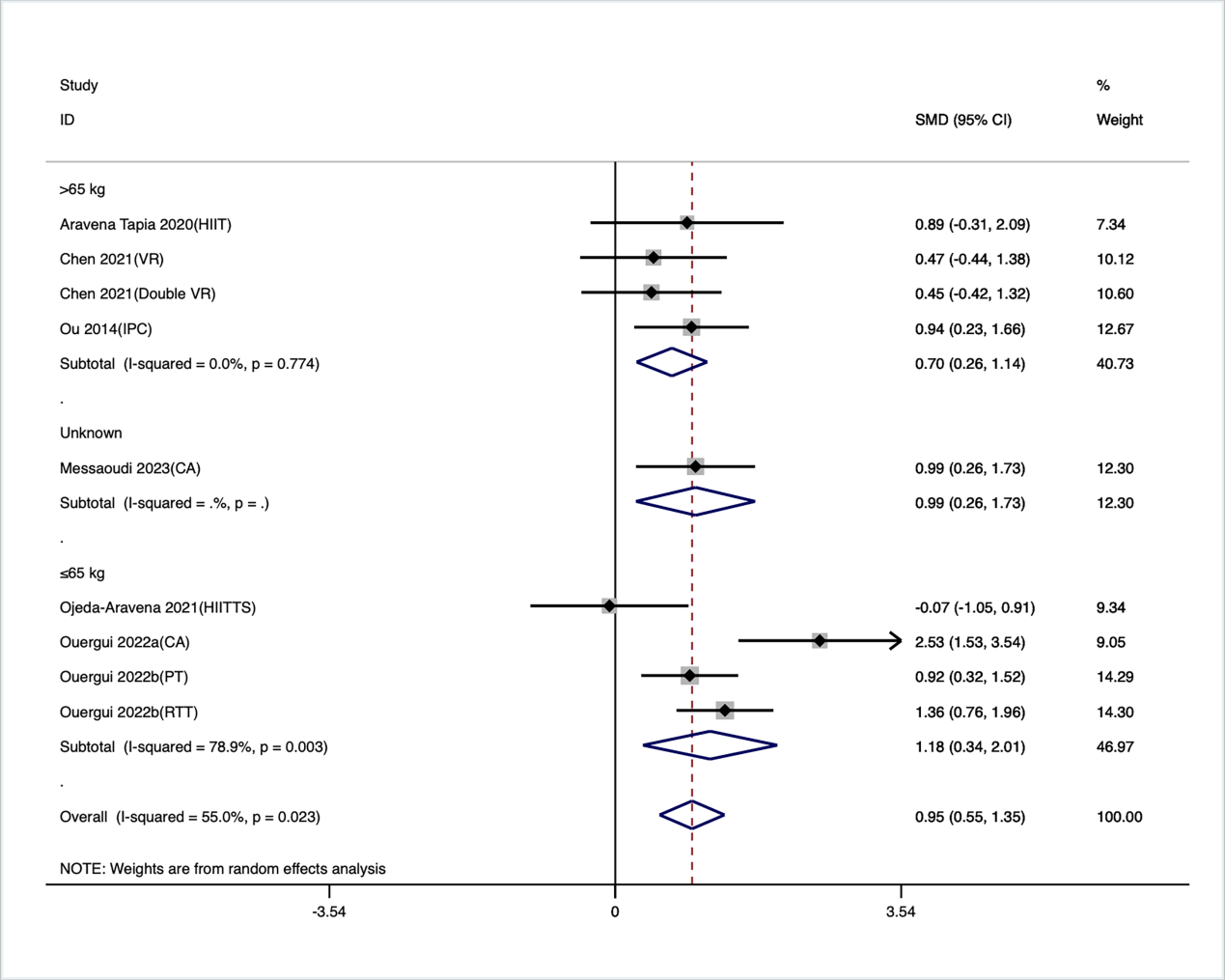
**

**Training experience**

**
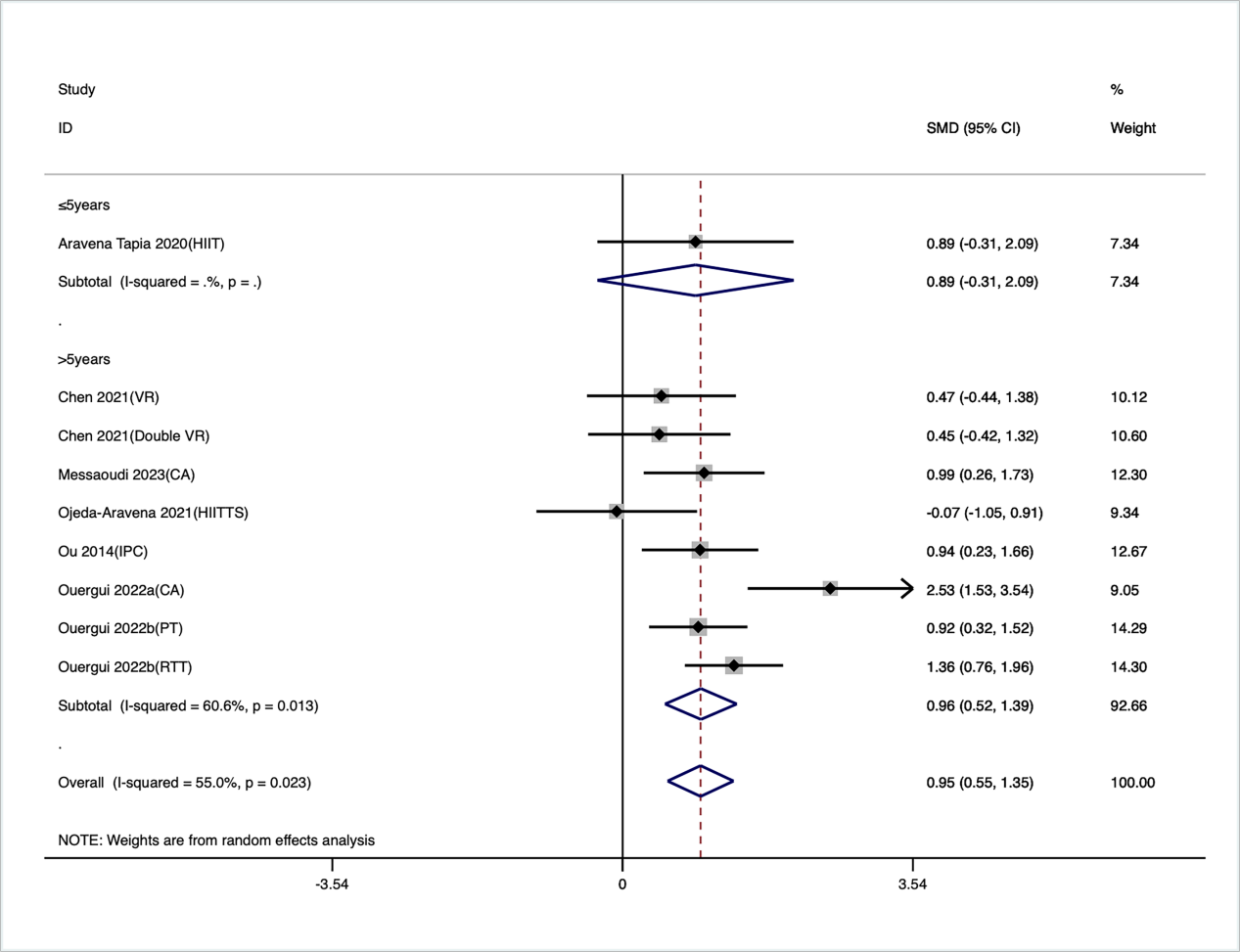
**

**VO_2_max:**

**Type of intervention**

**
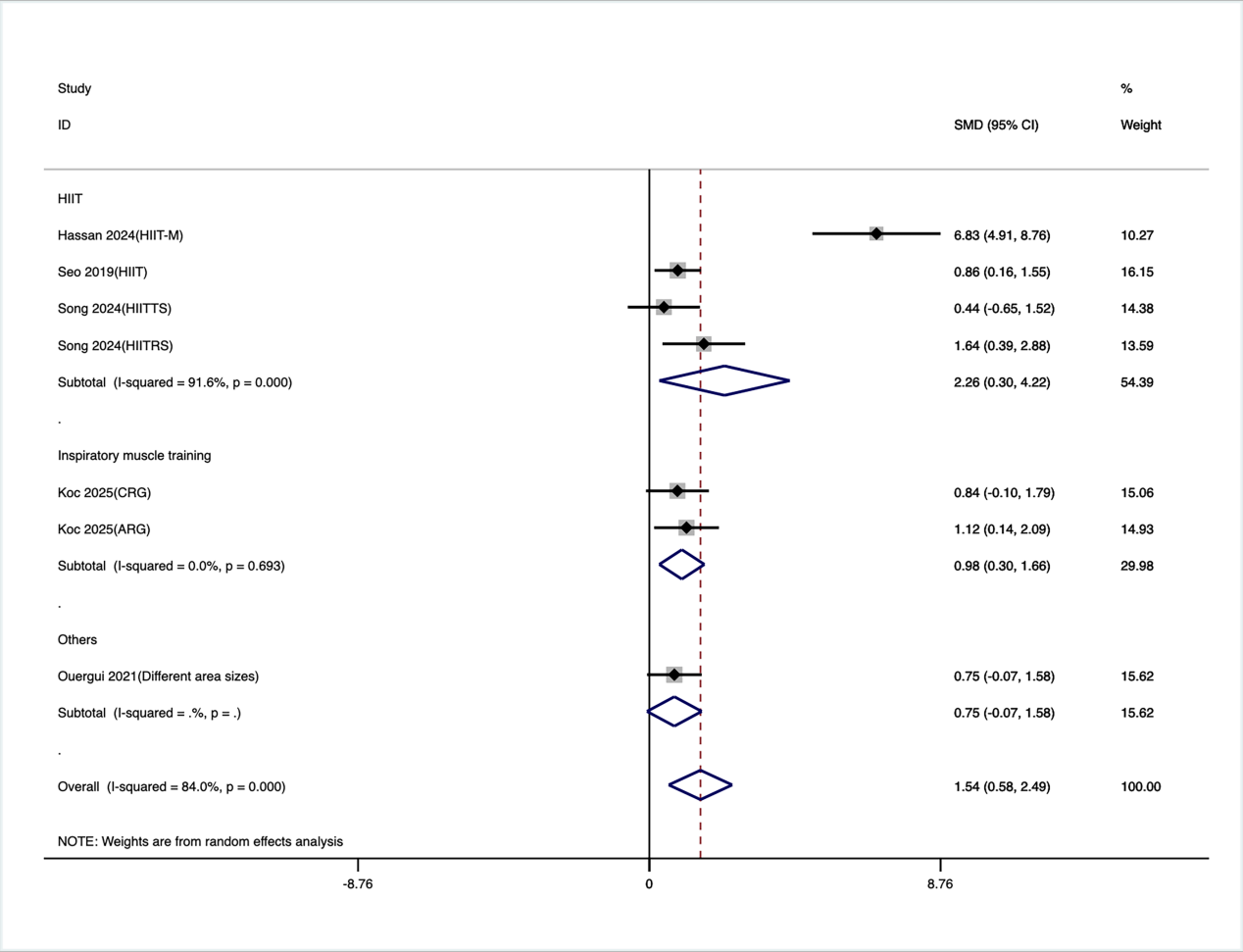
**

**Sex**

**
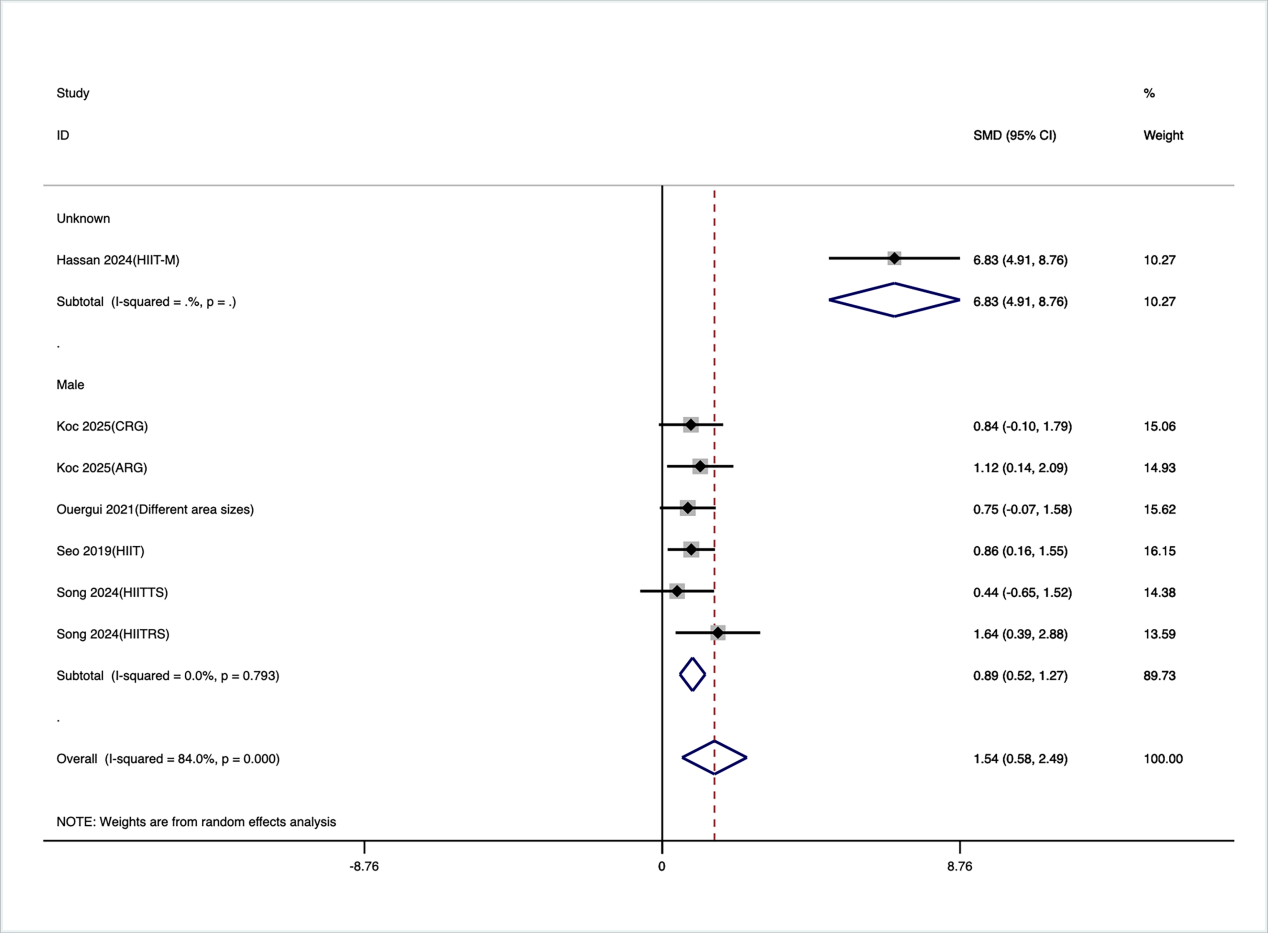
**

**Body weight**

**
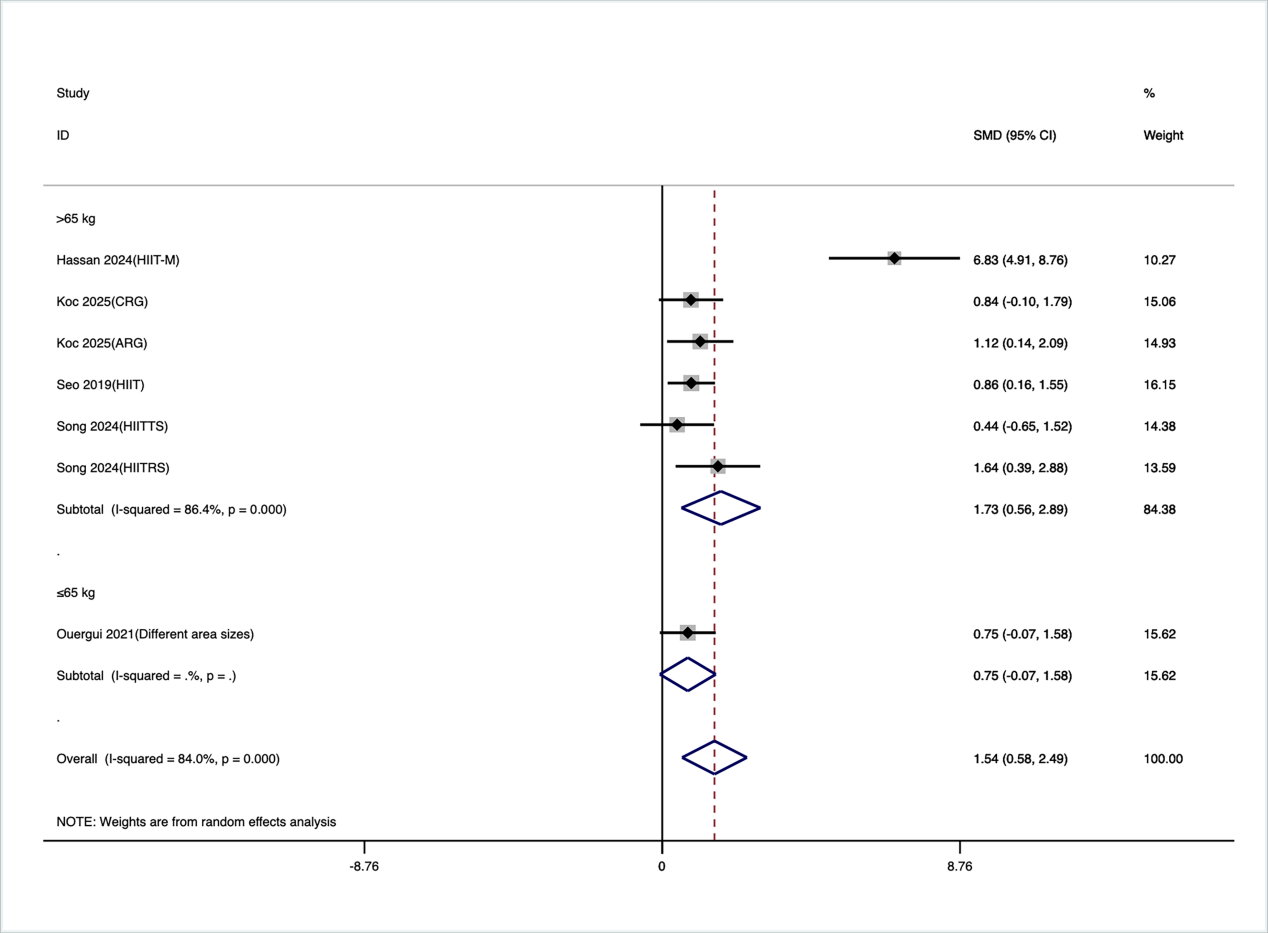
**

**Training experience**

**
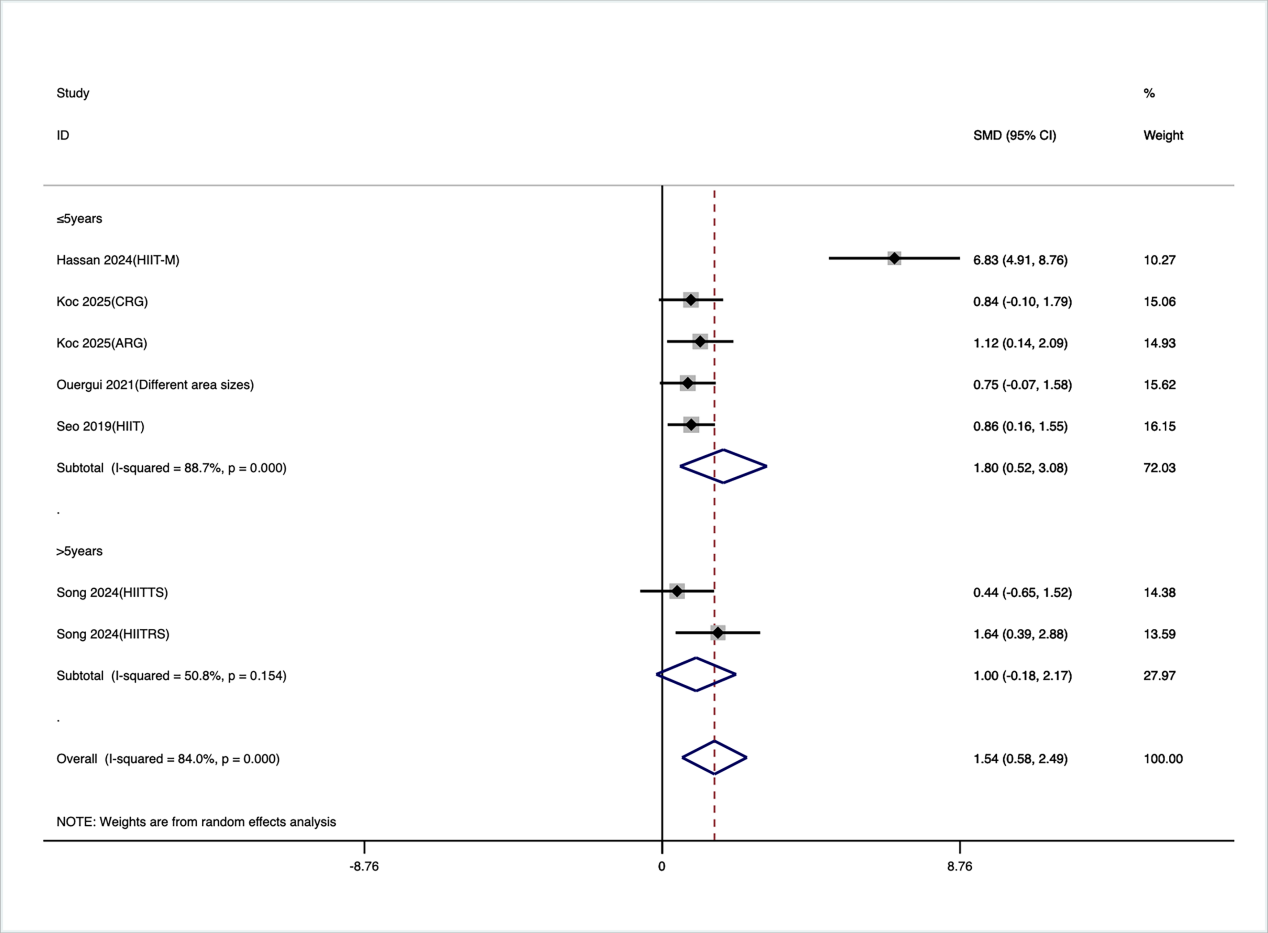
**

**nutrition supplementation**

**TSAT:**

**Intervention duration**

**
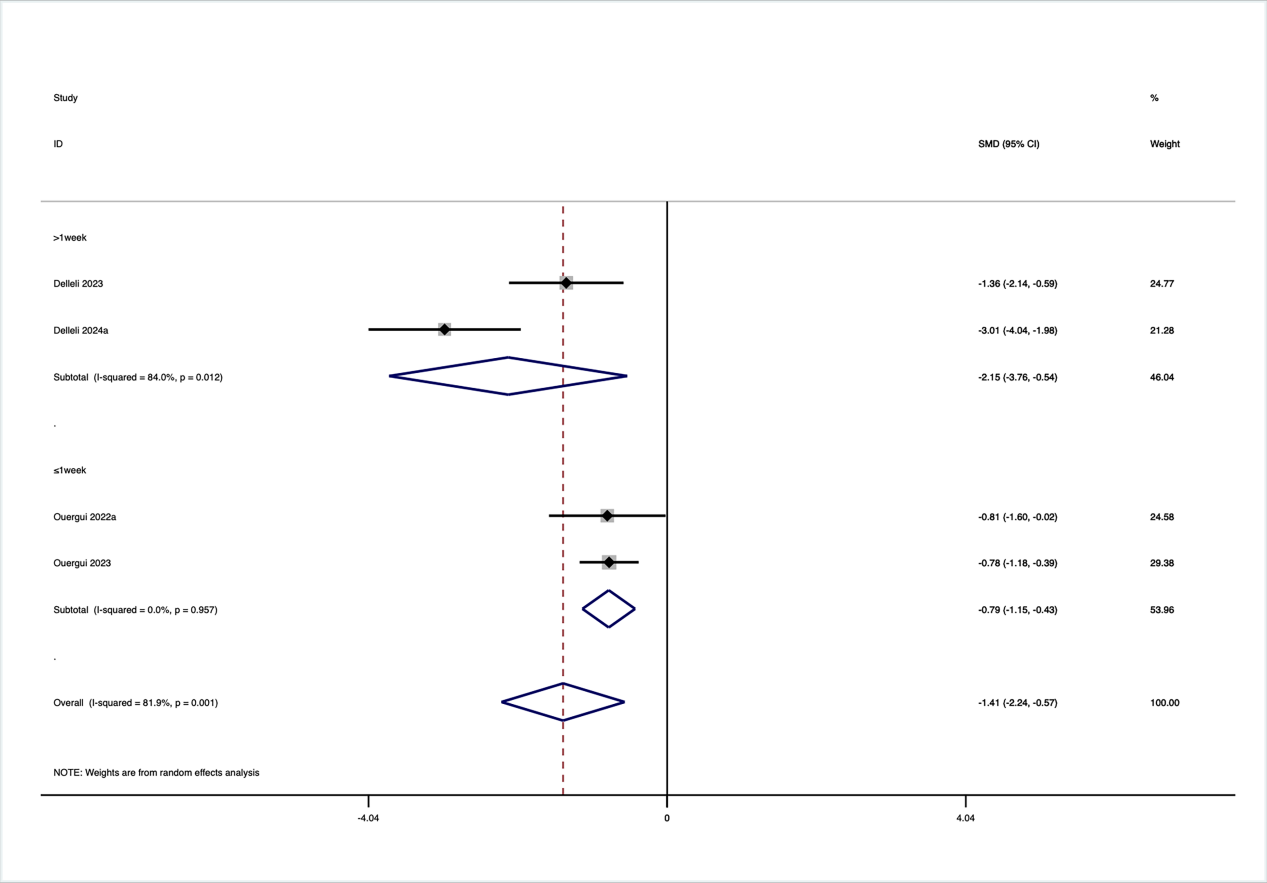
**

**Training experience**

**
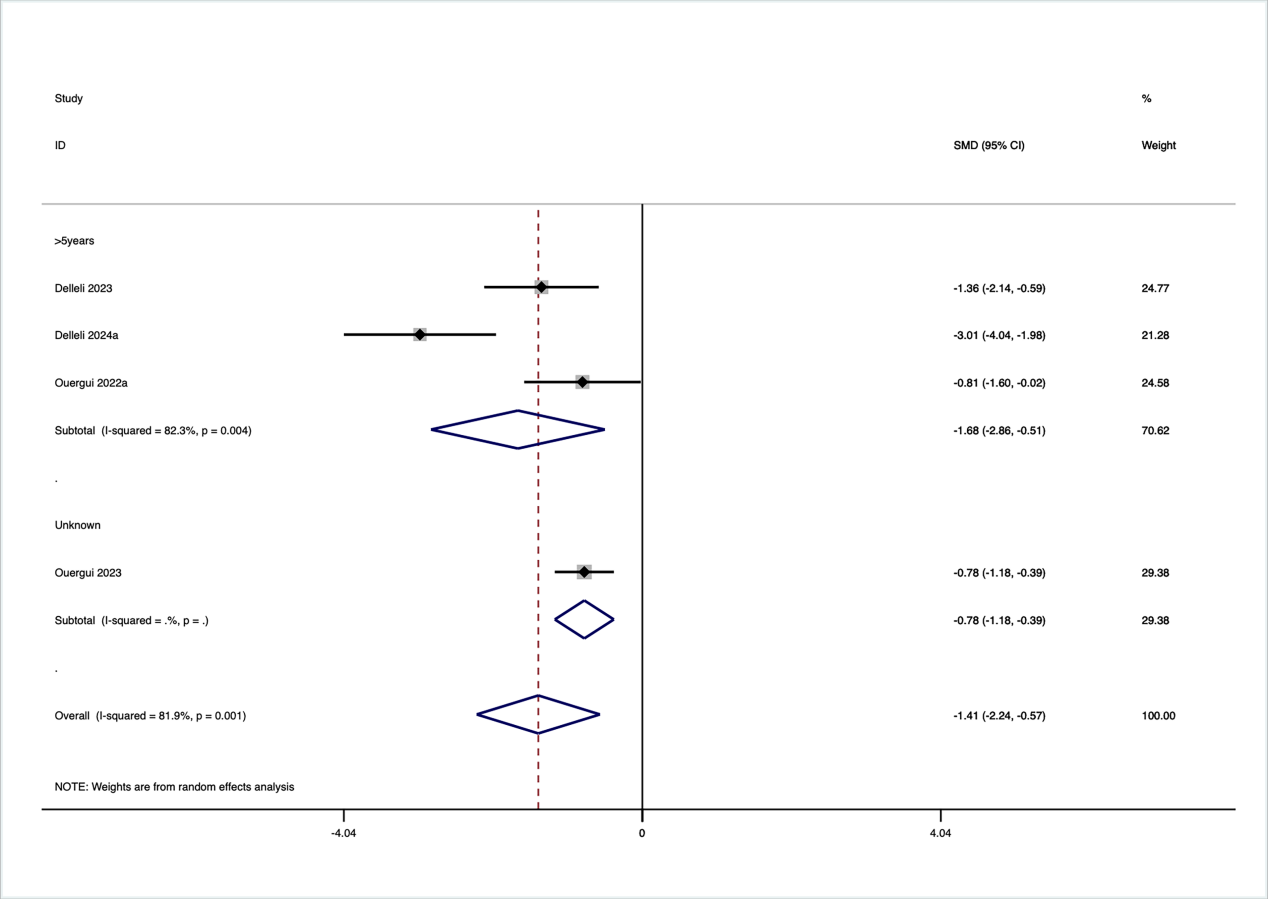
**

**FSKT-10s:**

**Intervention duration**

**
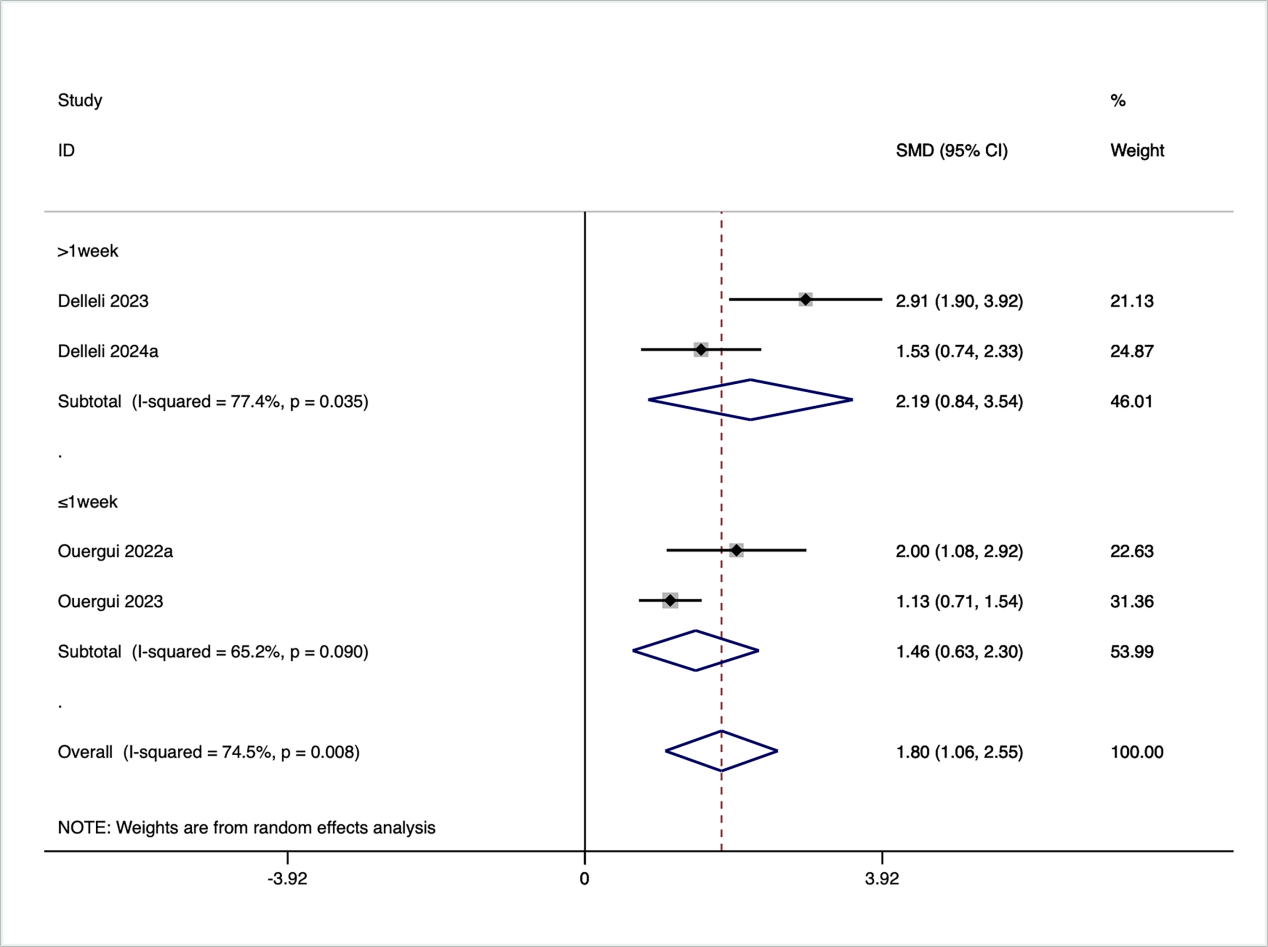
**

**Training experience**

**
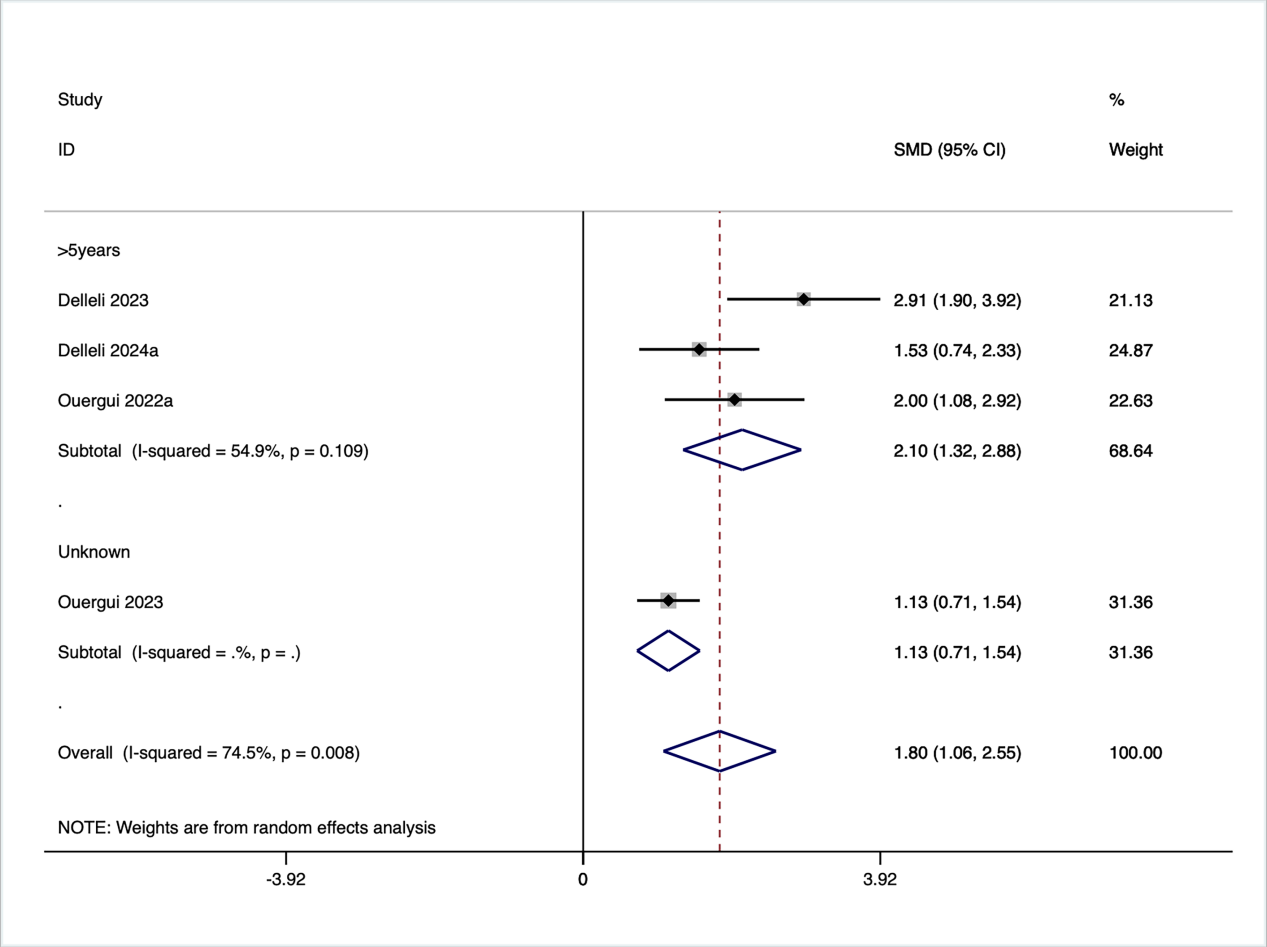
**

**FSKT-mult:**

**Intervention duration**

**
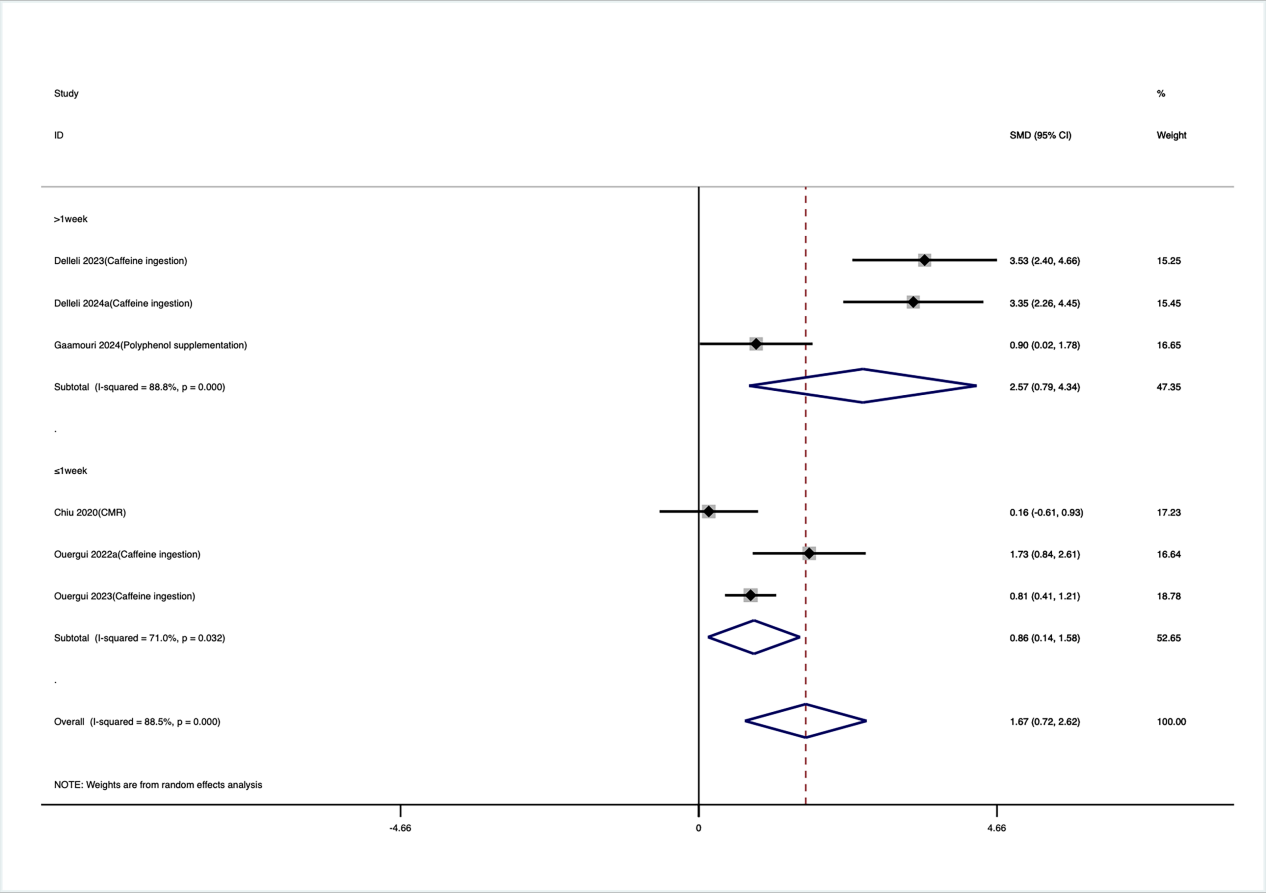
**

**Type of nutritional intervention**

**
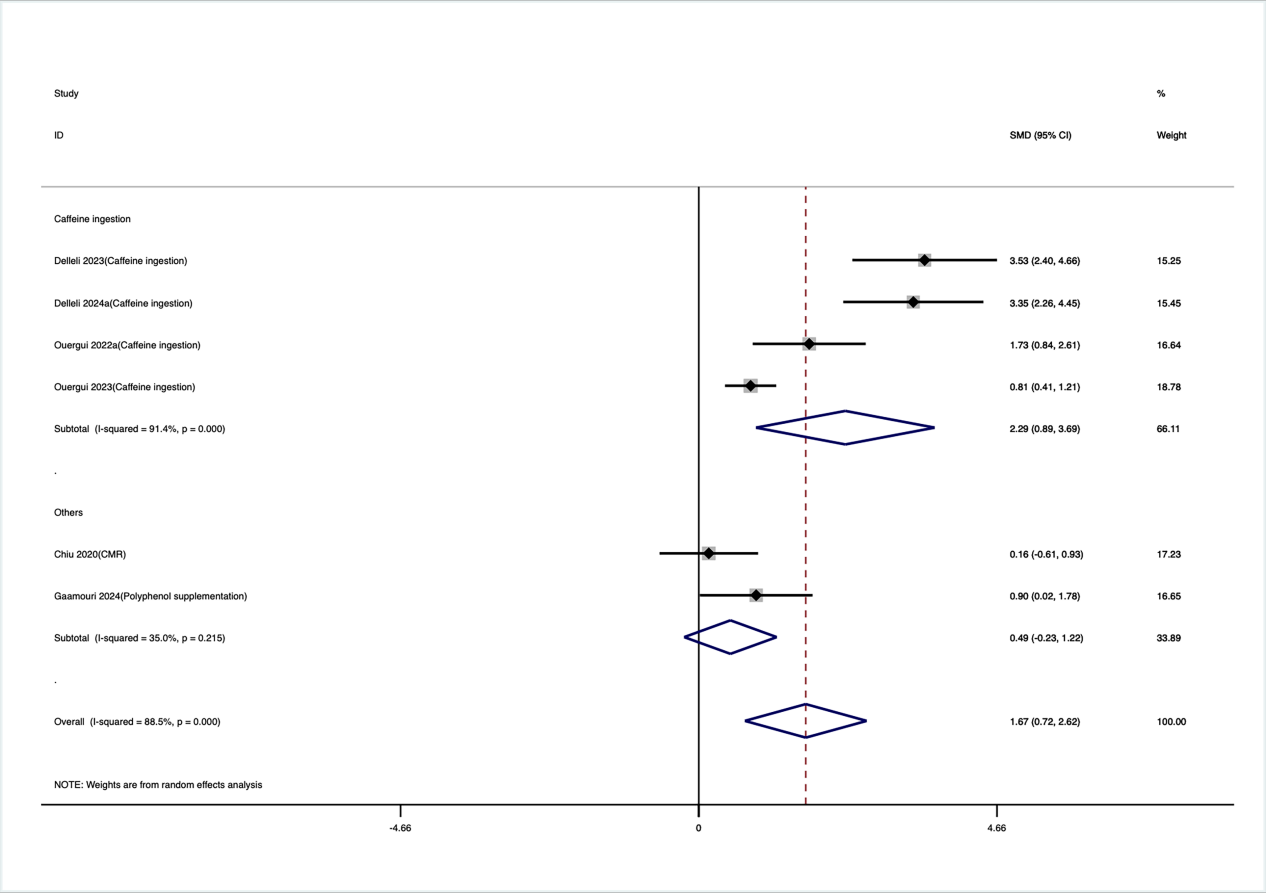
**

**Sex**

**
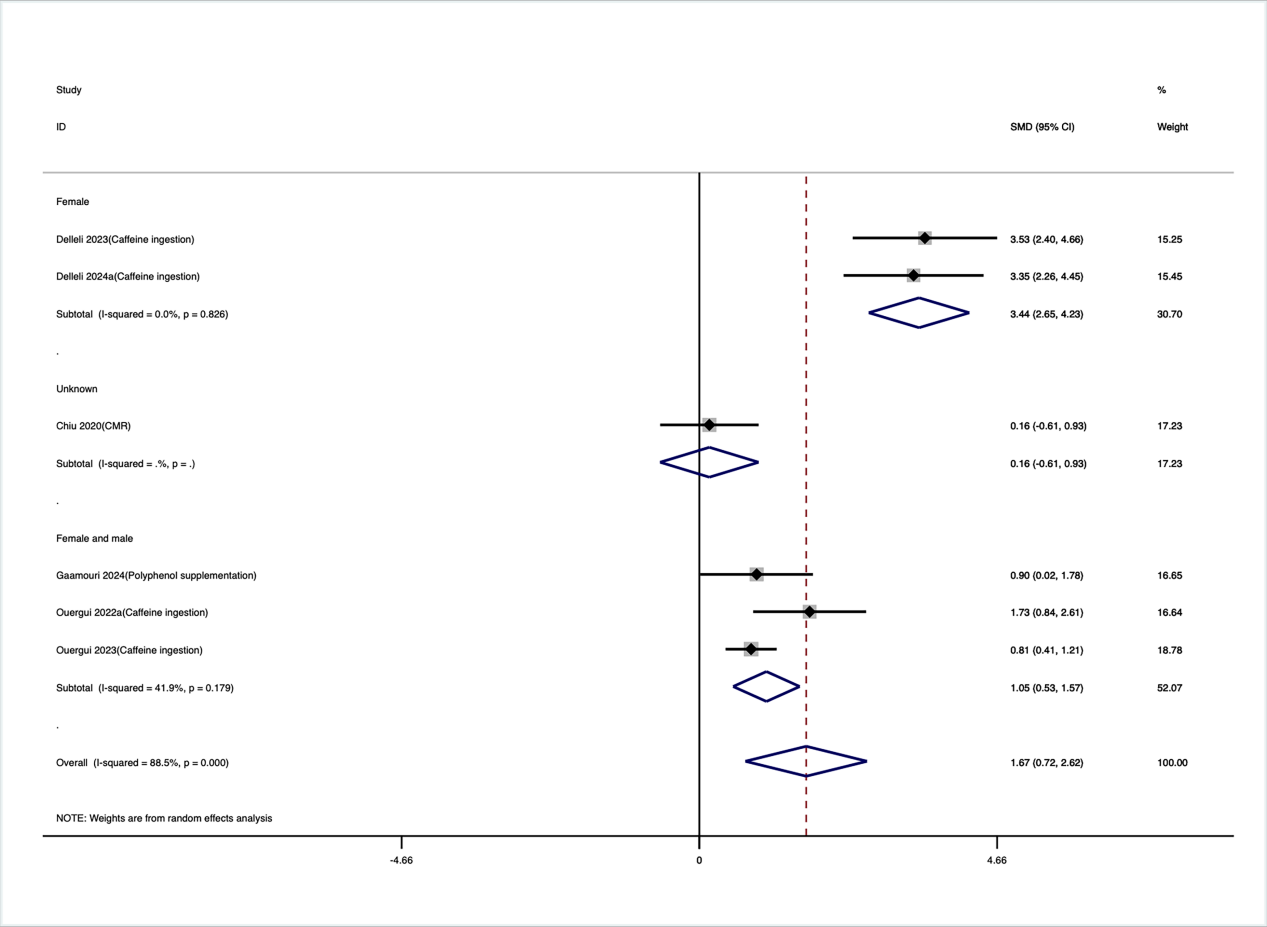
**

**Study design**

**
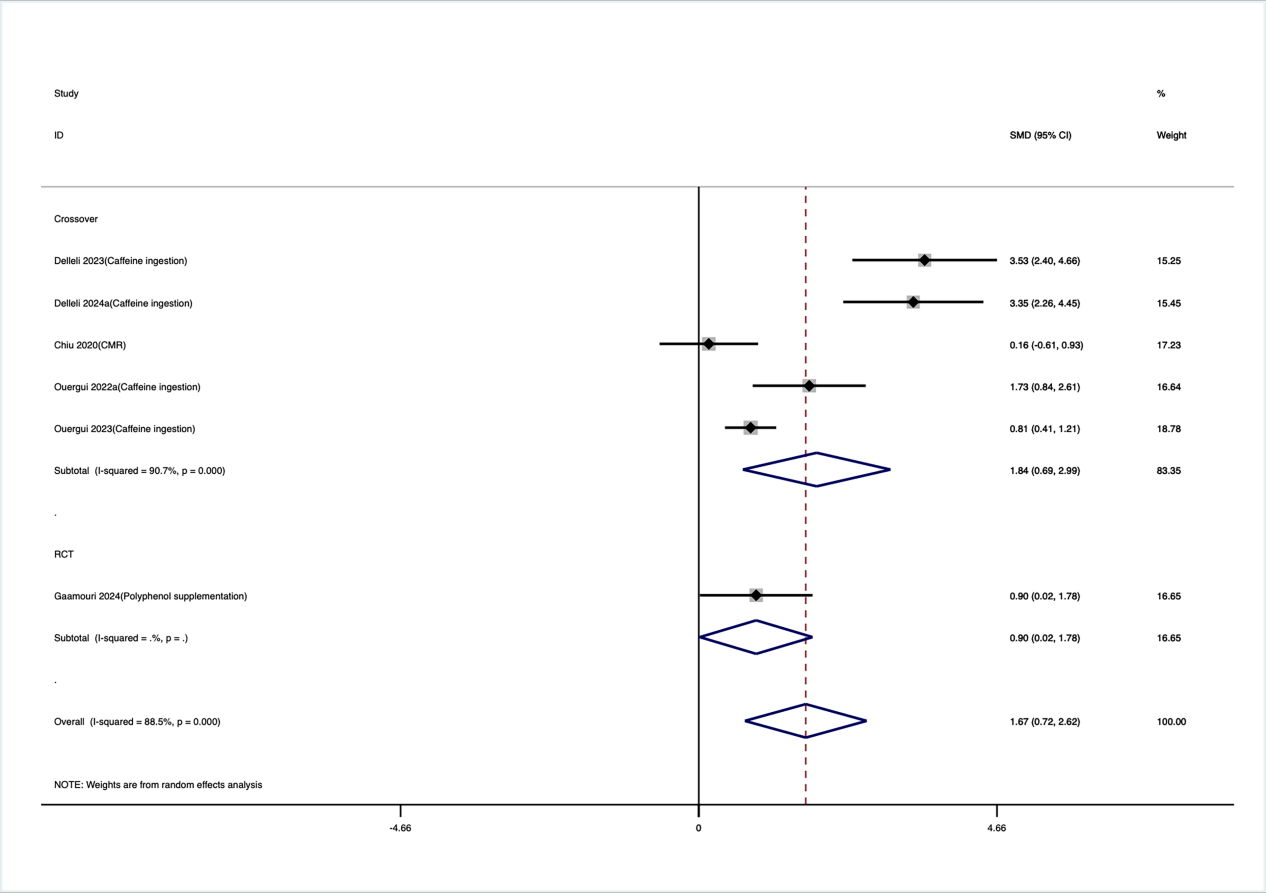
**

**Training experience**

**
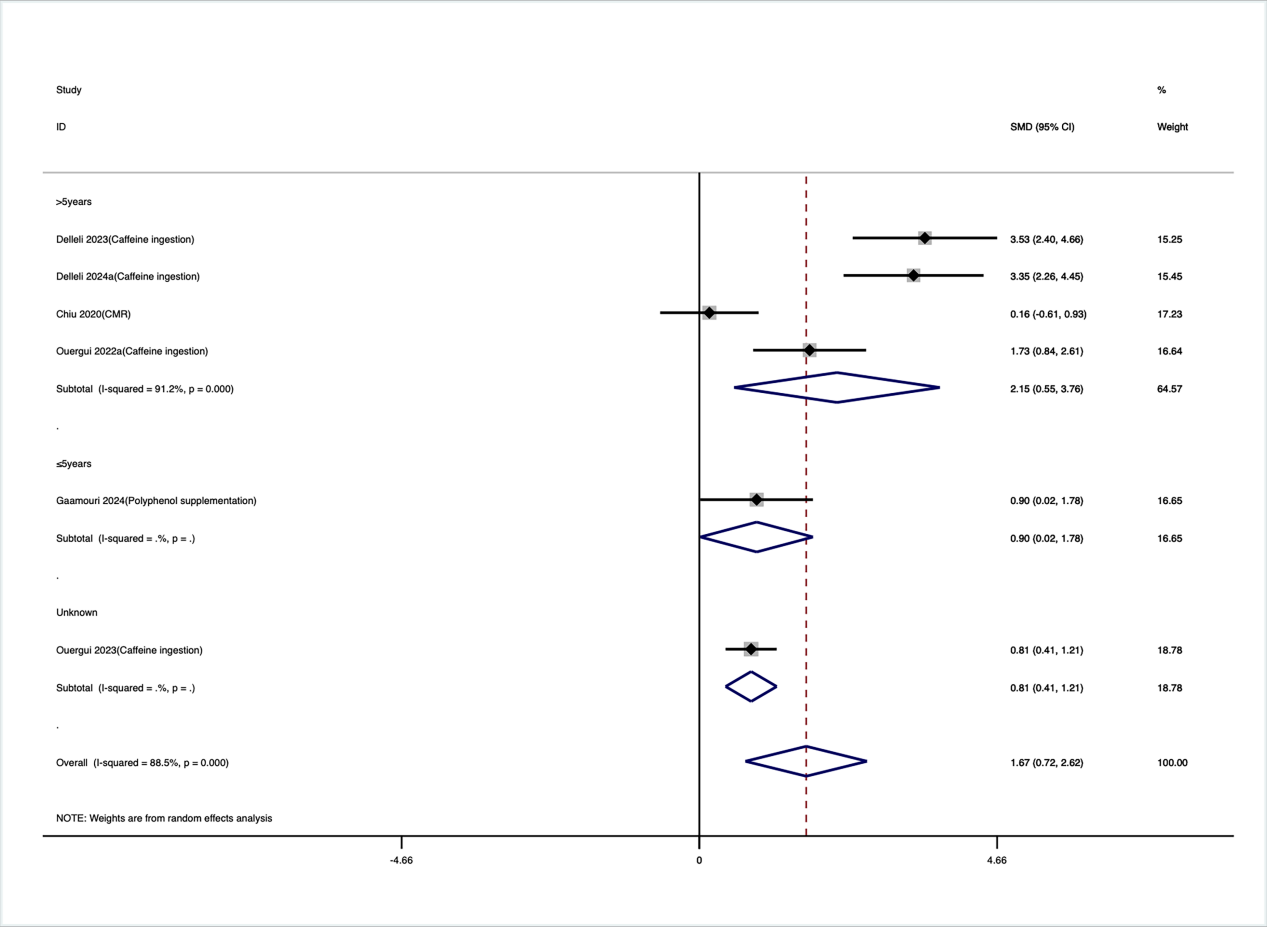
**

**Appendix 5: Begg’s Funnel Plots**

Exersice：
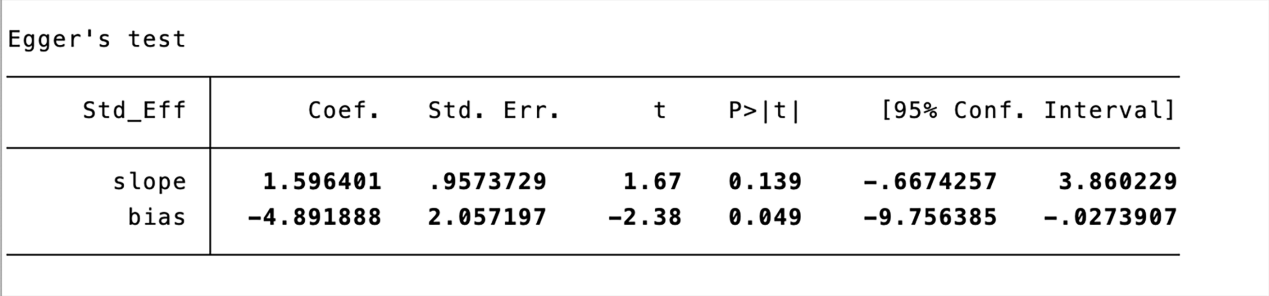


Figure1 :Egger’s test of TSAT.


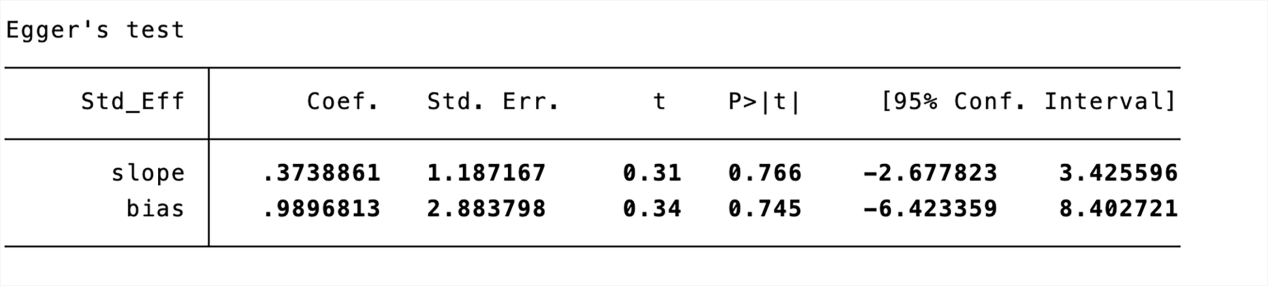


Figure2 :Egger’s test of FSKT-10s.


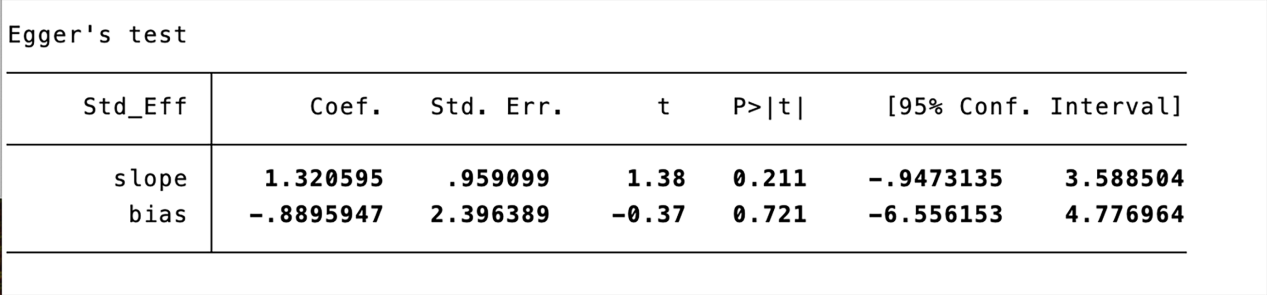


Figure3 :Egger’s test of FSKT-mult.


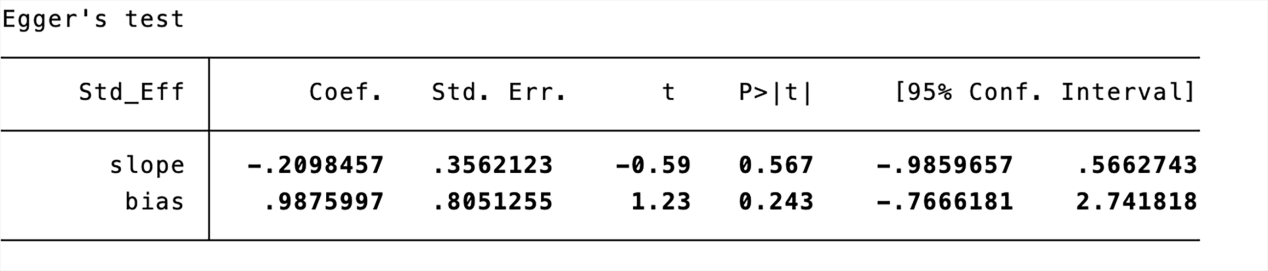


Figure4 :Egger’s test of CMJ.


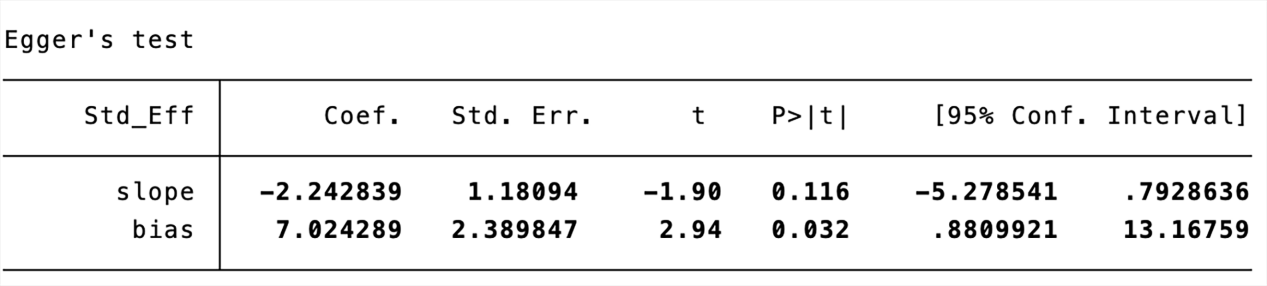


Figure5 :Egger’s test of VO_2_max.


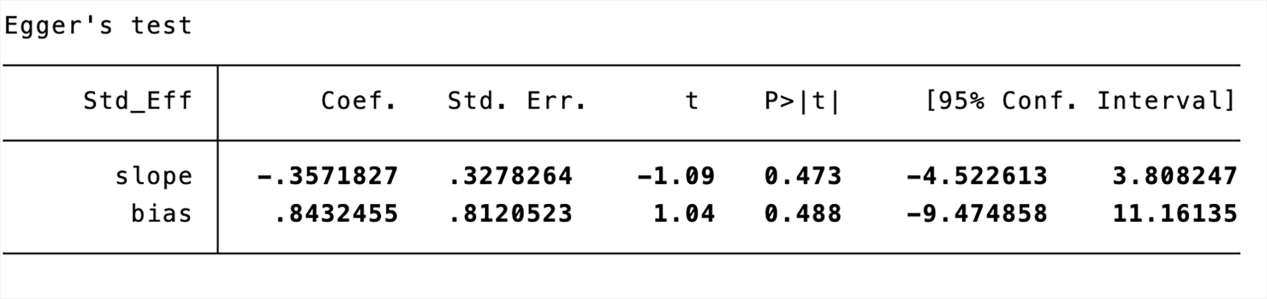


Figure6 :Egger’s test of HRmax.

Nutrition：
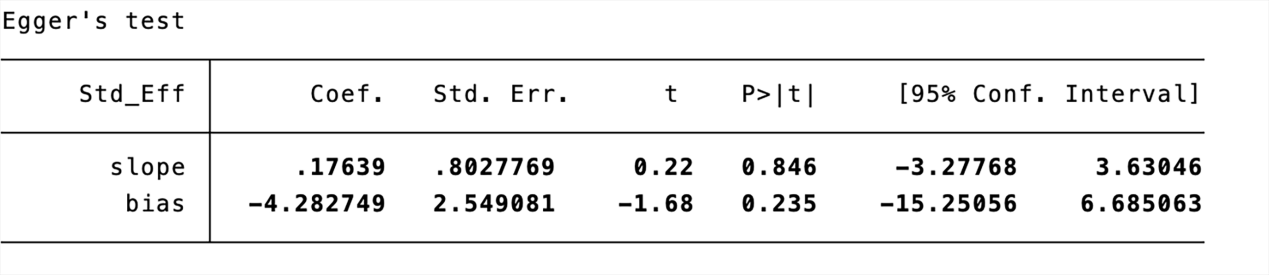


Figure7 :Egger's test of TSAT.


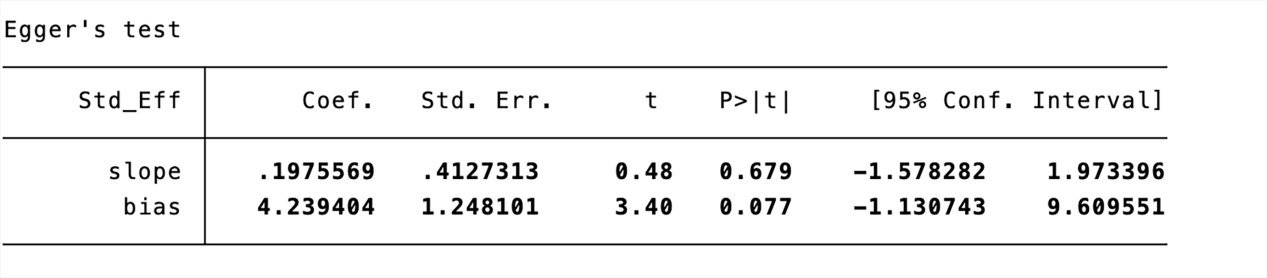


Figure8 :Egger's test of FSKT-10s.


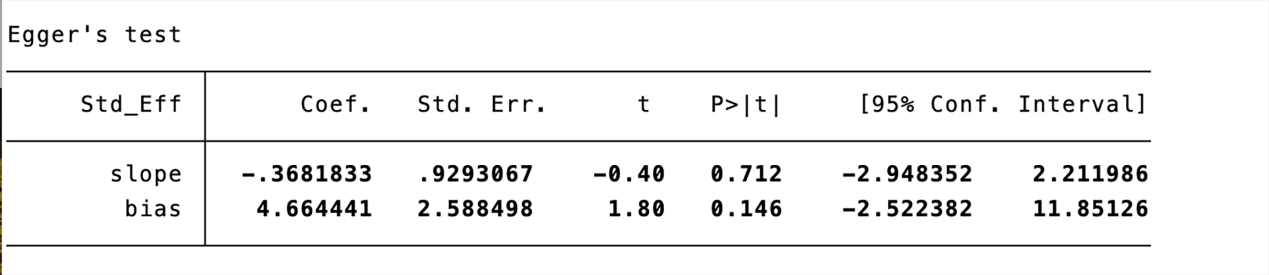


Figure9 :Egger's test of FSKT-mult.


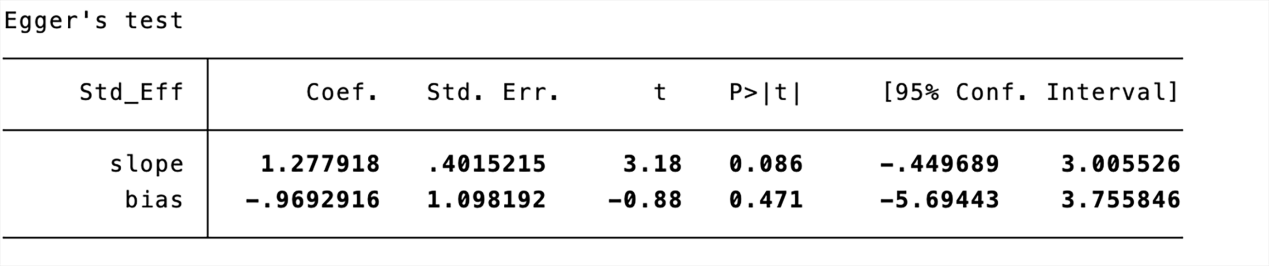


Figure10 :Egger's test of VO_2_max.


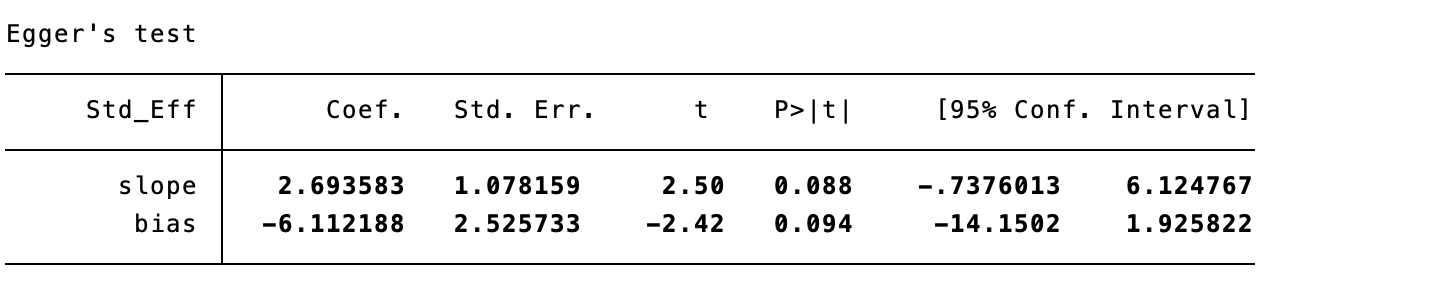


Figure11 :Egger's test of HRmean.


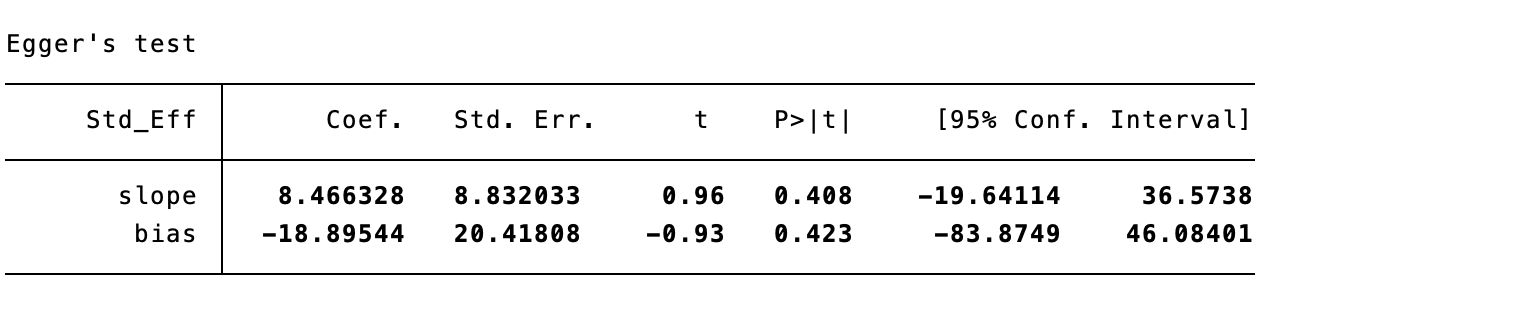


Figure12 :Egger's test of HRpeak.

(1)Exercise Intervention


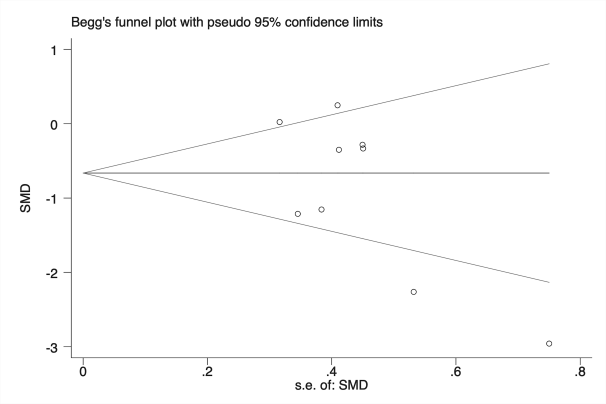

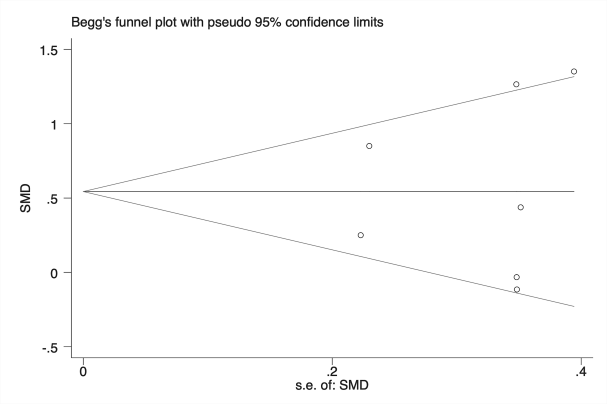


Figure1 :Begg test of TSAT. Figure2 :Begg test of FSKT-10s.


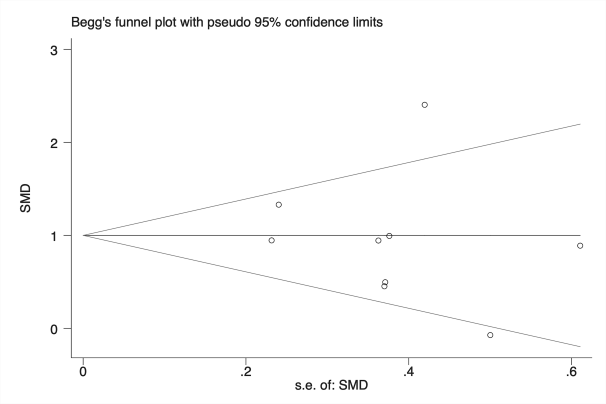

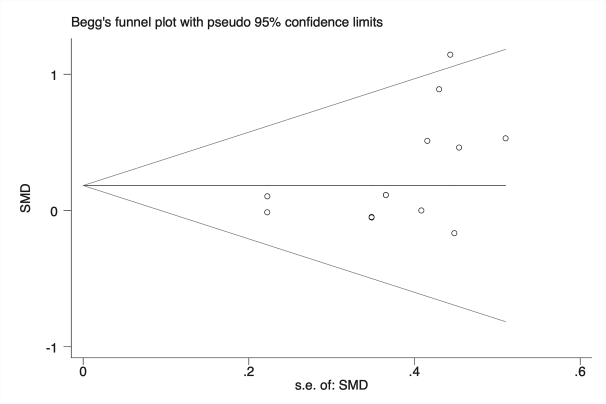


Figure3 :Begg test of FSKT-mult. Figure4 :Begg test of CMJ.


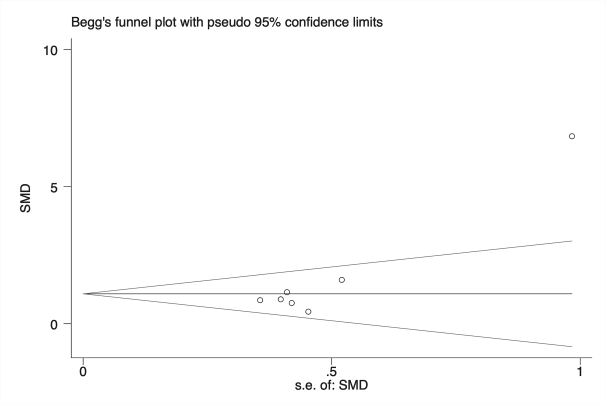

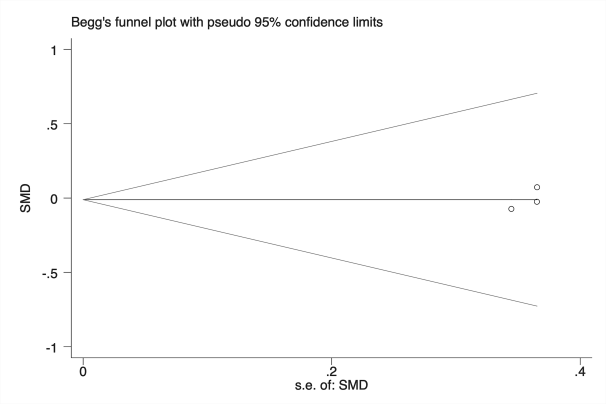


Figure5 :Begg test of VO_2_max. Figure6 :Begg test of HRmax.

(2)Nutritional Intervention


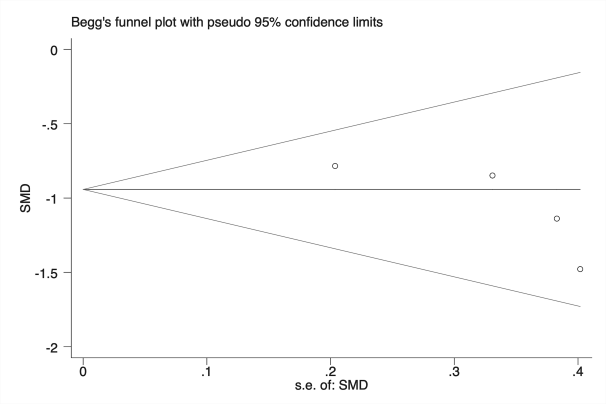

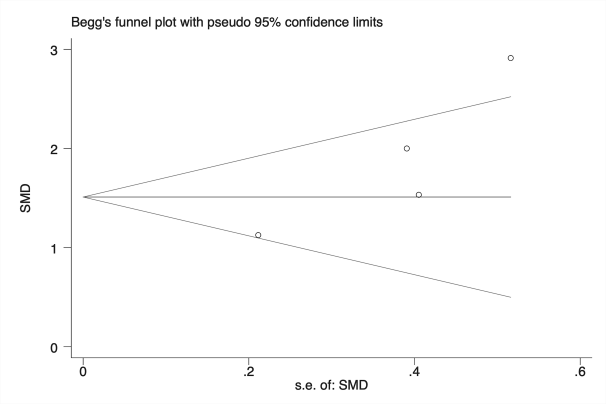


Figure7 :Begg test of TSAT. Figure8 :Begg test of FSKT-10s.


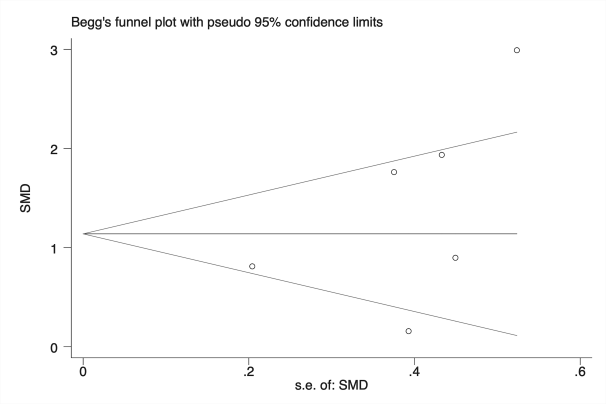

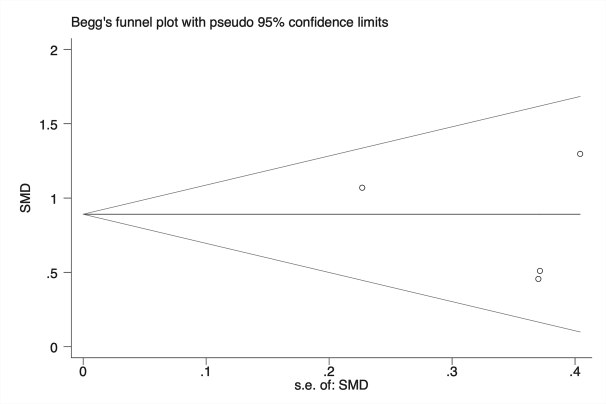


Figure9 :Begg test of FSKT-mult. Figure10 :Begg test of VO_2_max.


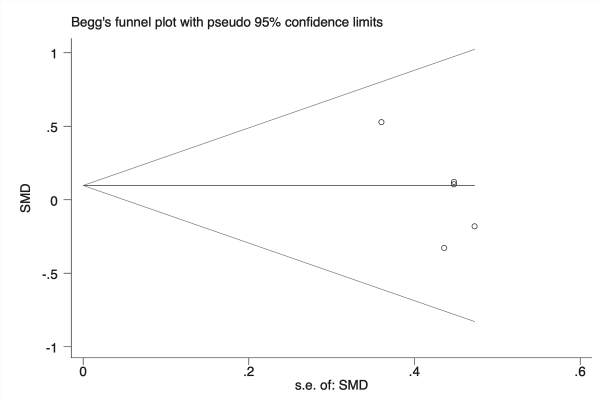

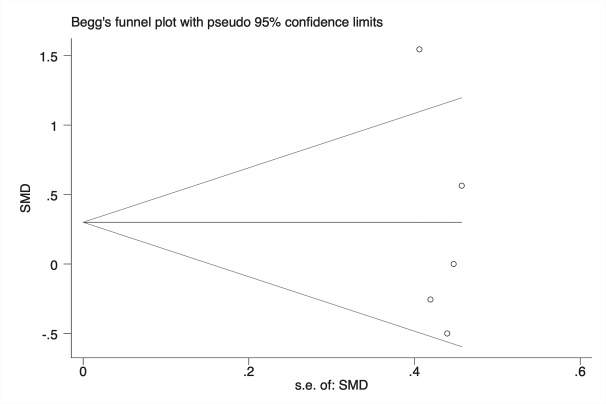


Figure11 :Begg test of HRmean. Figure12 :Begg test of HRpeak.

1. **Nutritional Int**
